# An Italian dinosaur Lagerstätte reveals the tempo and mode of hadrosauriform body size evolution

**DOI:** 10.1038/s41598-021-02490-x

**Published:** 2021-12-02

**Authors:** Alfio Alessandro Chiarenza, Matteo Fabbri, Lorenzo Consorti, Marco Muscioni, David C. Evans, Juan L. Cantalapiedra, Federico Fanti

**Affiliations:** 1 Grupo de Ecología Animal, Centro de Investigacion Mariña, Universidade de Vigo, 36310 Vigo, Spain; 2 Field Museum of Natural History, Chicago, IL 60605 USA; 3 Department of Mathematics and Geosciences, University of Trieste, 34128 Trieste, Italy; 4 Geological Survey of Italy (ISPRA), 00144 Rome, Italy; 5 Dipartimento di Scienze Biologiche, Geologiche e Ambientali, Alma Mater Studiorum, Università Di Bologna, 40126 Bologna, Italy; 6 Department of Ecology and Evolutionary Biology, University of Toronto, Toronto, ON M5S 3B2 Canada; 7 Department of Natural History, Royal Ontario Museum, Toronto, ON M5S 2C6 Canada; 8 GloCEE—Global Change Ecology and Evolution Research Group, Departamento de Ciencias de la Vida, Universidad de Alcalá, 28801 Alcalá de Henares, Spain

**Keywords:** Palaeontology, Biogeography

## Abstract

During the latest Cretaceous, the European Archipelago was characterized by highly fragmented landmasses hosting putative dwarfed, insular dinosaurs, claimed as fossil evidence of the “island rule”. The Villaggio del Pescatore quarry (north-eastern Italy) stands as the most informative locality within the palaeo-Mediterranean region and represents the first, multi-individual Konservat-Lagerstätte type dinosaur-bearing locality in Italy. The site is here critically re-evaluated as early Campanian in age, thus preceding the final fragmentation stages of the European Archipelago, including all other European localities preserving hypothesized dwarfed taxa. New skeletal remains allowed osteohistological analyses on the hadrosauroid *Tethyshadros insularis* indicating subadult features in the type specimen whereas a second, herein newly described, larger individual is likely somatically mature. A phylogenetic comparative framework places the body-size of *T. insularis* in range with other non-hadrosaurid Eurasian hadrosauroids, rejecting any significant evolutionary trend towards miniaturisation in this clade, confuting its ‘pygmy’ status, and providing unmatched data to infer environmentally-driven body-size trends in Mesozoic dinosaurs.

## Introduction

**Figure 1:**
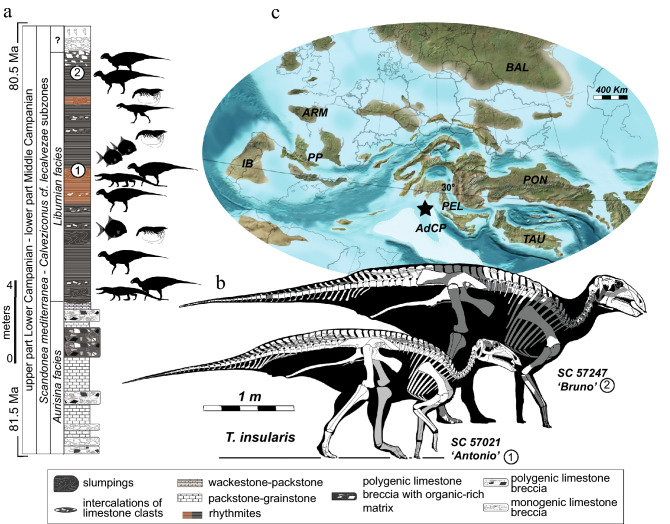
Geology and geographic context of Villaggio del Pescatore (VdP): chronostratigraphic context (**a**) defining the temporal setting where *Tethyshadros insularis* (**b**) and other fossil animals were found. Relative position (star) in the palaeogeography of the Tethys (**c**) where VdP most likely originated. Palaeogeographic abbreviations: IB, Iberian Landmass; ARM, Armorican Massif; PP, Pyrenean-Provencal Landmass; AdCP, Adriatic-Dinaric Carbonate Platform; PON, Pontides Orogen; TAU, Taurus Block; BAL, Baltic Landmass. White coloured bones in SC 57247 (b) are those recovered in the fossil and which we were able to base the reconstruction on (e.g. the outline of the sacral neural spines is based on SC 57021). Source map^©^ 2020 Colorado Plateau Geosystems Inc. Silhouette credits: *Tethyshadros*—Marco Muscioni (CC-BY 4.0); *Aucasaurus garridoi *and *Brachychampsa*—Scott Hartman/Phylopic (CC-BY-NC-SA 3.0); *Proscinetes elegans* Dean Schnabel/Phylopic; *Penaeus*—Christoph Schomburg/Phylopic (CC0 1.0).

The latest Cretaceous Mediterranean archipelago, a complex set of carbonate platforms, peninsulas, and islands in the western margin of the Tethys Ocean, bracketed by Laurasian and Gondwanan continental remains of Pangea, represents a long-lasting challenge for palaeogeographers and palaeontologists focused on non-marine vertebrates, their evolution and biogeography^1^. Most of vertebrate remains documenting the evolution of this unique context are confined to the Adriatic Carbonatic Platforms (AdCP), a vast domain characterized by carbonate platforms severed by deeper marine areas. The AdCP was ecologically set apart from other neighbouring larger European landmasses (i.e. Iberian, Pyrenean-Provencal, Pontid, and Pelagonian domains), where more continental environments and faunas developed, playing a pivotal role in biogeographic reconstructions for the latest Cretaceous^2,3^. The gradual reduction of the AdCP through compressive tectonic events from the late Campanian arguably limited dispersal events across the palaeo-Mediterranean and may have fostered the sustained miniaturisation^1^ in response to adaptive changes in several lineages, including non-avian dinosaurs^2^. To this date, the geodynamic evolution, ecological diversity, connections with adjacent landmasses, and faunal composition of the AdCP stand as one of the most complex and debated topics related to the Tethyan evolution. Fundamental limitations in our understanding of evolutionary and biogeographic patterns are represented by (1) limited palaeogeographic data documenting the real geographic extent of carbonate platforms and their connection with other landmasses (e.g. Iberian-Provencal, Pelagonian and Balkan landmasses) and (2) rare fossil material commonly represented by ichnosites or poorly preserved skeletal remains^4^. Relevant to this study, available palaeogeographic maps of the AdCP during the Cretaceous suggest that purported ‘islands’ were actually vast—although intermittently emerged—areas that throughout the Late Cretaceous allowed subaerial connection between the central Mediterranean and Laurasia^4,5^. Therefore, terms as ‘isolated landmasses’ or ‘island’ should be used cautiously in the case of the Villaggio del Pescatore locality (VdP herein; Fig. 1) and other AdCP sites to avoid a potential misinterpretation of the regional biogeographic relevance of its fossil fauna^6^. In this context, Italy plays a key role due to its geographical position and by preserving the sole latest Cretaceous dinosaur-dominated site in the AdCP system, namely the VdP (Fig. 1). This site was discovered about thirty years ago in the Upper Cretaceous–Palaeogene beds exposed near Duino Aurisina, Trieste, north-eastern Italy and produced, amongst fish, crustaceans, and plant remains, an exquisitely preserved articulated skeleton of the hadrosauroid *Tethyshadros insularis*^7^. Its complex geological setting has been addressed in several works^8–12^ but a contrasting variety of claims regarding the age of these deposits (late Santonian^8,13–17^; Santonian–Campanian^11,12^; late Campanian–early Maastrichtian^7,18^) has created controversy that directly impacts biogeographic contextualization and evolutionary interpretations^19^ (e.g. VdP as part of a Maastrichtian archipelago fostering insular adaptations^2^). Additionally, from a faunal perspective, the combination of purportedly ‘derived’ and ‘primitive’ characters occurring in the holotype of *T*. *insularis* (SC 57021)^7,20^, its diminutive size (~ 338 kg according to body-size regression estimates presented by Benson et al.^21^), and its suggested maturity, were used as evidence of insular dwarfism in this taxon. An early Maastrichtian age for the VdP site combined with phylogenetic analysis that placed *Tethyshadros* closely related to the supposedly coeval and island-dwarf *Telmatosaurus* from the ‘Transylvanian island’ of Romania^2^, set the ground for hypotheses regarding the evolution of a clade of dinosaurian insular dwarves in the latest Cretaceous^7,22^. Novel geological and fossil data from the VdP presented here finally represent the first set of evidence to test such hypotheses and provide insights into claimed insular adaptations and body size evolution in the latest Cretaceous dinosaur fauna of Europe.

**Figure 2:**
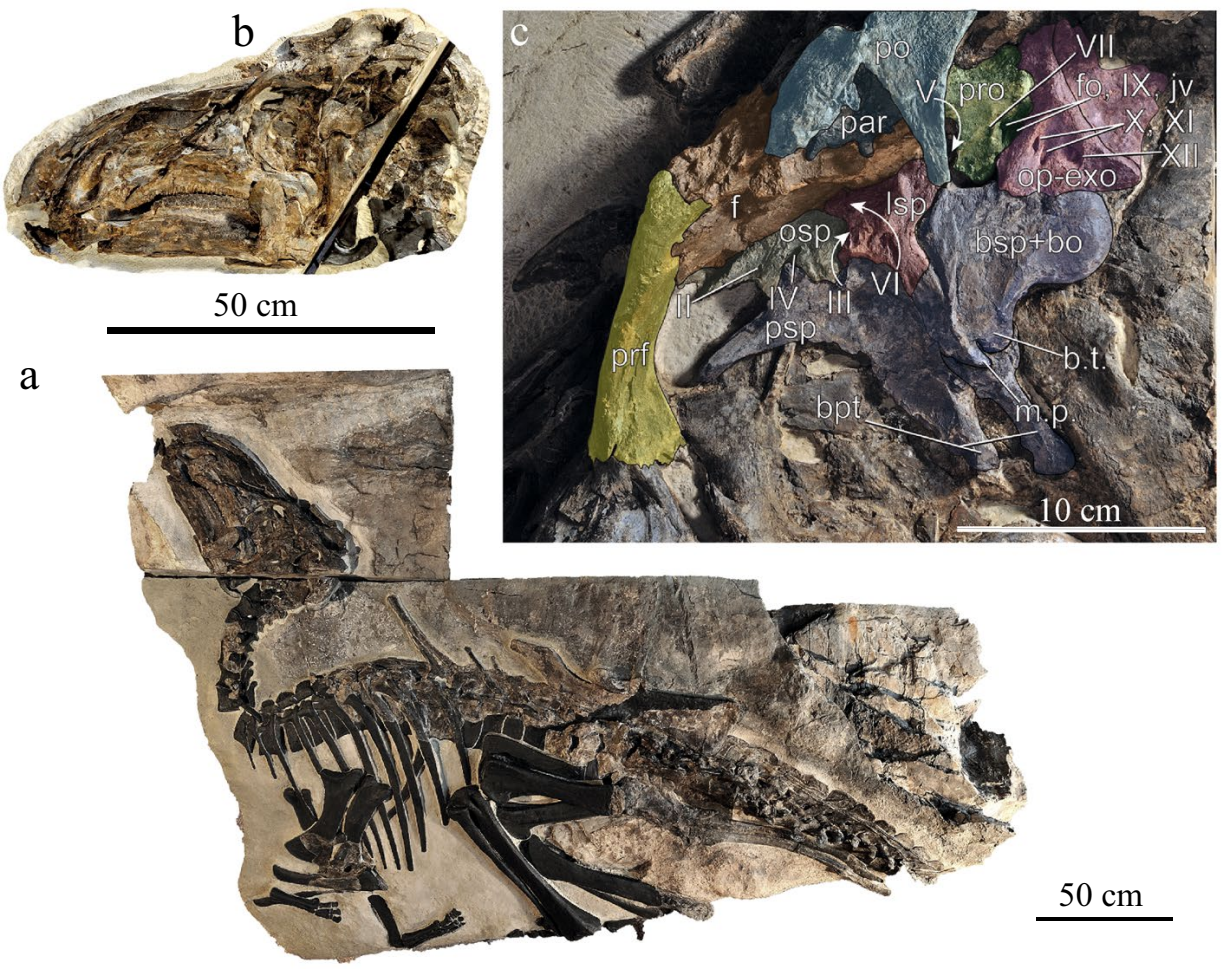
The new skeleton of *Tethyshadros insularis* (**a**) preserving details of its cranial anatomy like the nearly complete skull (**b**) exposing its braincase (**c**) adding important information for the anatomy and systematic of this taxon. Elements in black are reconstructed. Anatomical abbreviations, pro, prootic; po, postorbital; f, frontal; bo, basioccipital; bsp, basipshenoid; prf, prefrontal; par, parietal; bpt, basipterygoid processes of the basisphenoid; m.p. median process of the basisphenoid; b.t., basitubera; lsp, laterosphenoid; jv, exit of the jugular vein; fo, fenestra ovalis; op-exo, opistothic-exoccipital complex; cranial nerve numbers follow roman enumeration.

In this study we reevaluate the age of the VdP site and describe new material referred to *T*. *insularis* (Fig. 1; Fig. S1; Supplementary information S1) challenging current interpretations on its pygmy and insular status, and discussing the geographic role of the site and its fauna in the latest Cretaceous. New biostratigraphic data indicate that the VdP site (see details in Supplementary Information S1) is latest early Campanian–earliest middle Campanian in age, and represents a time interval of roughly 1 My, comprised between 81.5 and 80.5 Ma (Supplementary Information S1). Relevant to taxonomic interpretations presented here, the finely laminated, fossil-bearing rhythmites represent a much shorter interval estimated in a few thousand years (see details in Supplementary Information S1). We describe a new, remarkably well-preserved, and articulated individual (SC 57247; Fig. 2) and introduce the material of six additional skeletons of *T*. *insularis*: given the significantly larger size of one of these individuals compared to the holotype, we inferred the ontogenetic stages of these specimens using osteohistology. Consequently, we revise the former description of the holotype by documenting morphological variation in this taxon and highlighting the ontogenetically variable characters in our sample. Finally, we evaluate the phylogenetic position of *Tethyshadros* and use a comparative phylogenetic framework^23,24^ combining ancestral state reconstruction and Ornstein–Uhlenbeck (Hansen) models to test whether the evolution of body-size was following a significant, anomalous, and accelerated trend of body-size reduction in the clade which includes *Tethyshadros*. Our results challenge previous hypotheses supporting events of dwarfism among ornithischians during the Late Cretaceous and support the presence of plesiomorphically average-sized hadrosauroids invading the Tethyan domain from Eurasia and distinct from the later and more fragmentated, insular environment of the Maastrichtian European archipelago.

## Results

### Geology and revised age of the VdP fossil assemblage

Fossil beds composing the VdP site pertain to the lower part of the Liburnian limestone facies and are represented by a ~ 10 m thick interval of slumped, black to beige, organic rich carbonate rhythmites (see Supplementary Information S1). These marginal-marine finely laminated layers (Fig. 1a), unconformably overlying the limestone-dominated *Aurisina* facies, are responsible for the exquisite preservation of dinosaurs, small crocodyliforms, a single pterosaur bone, partial fishes, several crustacean taxa, rare coprolites, pollen, and algae^7,10,20^. The outstanding example of a new individual of *T*. *insularis*, SC 57247, shows how the mesoscale folding has interested the fossil body harmoniously but maintaining most of the anatomical connections exquisitely preserved (Fig. 2; Figs. S15, S16). Deposits preserved as underwater slumps also provide critical data concerning the taphonomy (preservation of large land vertebrates into dysoxic to anoxic bottom waters in marginal-marine settings) as well as the interaction between different depositional settings (terrestrial–shallow marine) as they have no equivalent in any Mesozoic carbonate platforms so far. The age of the site is here revaluated based on new, biostratigraphic constraints (Supplementary Information S1). The succession referred to the *Aurisina* facies is here assigned to the *Accordiella conica* and *Rotalispira scarsellai* biozone of Chiocchini et al.^25,26^ and Frijia et al.^27^ based on biostratigraphically correlated Strontium Isotope Stratigraphy (SIS^27–30^), thus narrowing its chronostratigraphic position to the lower Campanian. Furthermore, the foraminifera *Accordiella conica*, *Murciella* gr. *cuvillieri*, and *Rotalispira* cf. *maxima* have been recovered within and in the uppermost, fossil-bearing, rhythmites interval. Given the low-complexity shells observed in our specimens, foraminifera morphotypes assigned to the genus *Murciella* are less inclusively assigned to *M*. gr. *cuvillieri*, a taxon that includes all the possible morphological variability observed into the *M. cuvillieri* type-population of the Campanian of Murcia Province in Spain (see Fourcade^31^). *M. gr. cuvillieri* has a chronostratigraphic range comparable to the occurrences found within the AdCP^28,32^ and referred to the lower Campanian or to the basal part of the middle Campanian. This time constraint (between 81.5 and 80.5 Ma; Supplementary Information S1) is also biostratigraphically and SIS-justified by the co-occurrence of specimens referable to *A. conica* and *R. scarsellai* (Fig. 1; Figs. S1–S3).

### Palaeogeography

The lower Campanian deposits of the VdP locality originated at the north-western margin of the Adriatic Carbonate Platforms system (AdCP) which relics extend today from north-eastern Italy to Turkey^6,33,34^. In the latest Cretaceous, these platforms reached a remarkable geographic extent, although detailed reconstructions of their margins are uncertain given a lack of informative exposures^4,35–37^. Several fossil localities preserving dinosaur bones and footprints throughout the Upper Cretaceous^38^ document long-lasting continental environments along the northeastern margin of the AdCP and connections with Eurasian landmasses. The fragmentation of the AdCP and consequent formation of more insular conditions occurred during the post-Campanian, as final stages of collisional processes led to the progressive dismounting of carbonate platforms^6^. As data presented in this study reassign the fossil bearing beds of the VdP site to the early to mid-Campanian age (Supplementary Information S1), the VdP assemblage is the only proxy for inferring dinosaur biogeography within the AdCP prior to the onset of the progressive drowning of the marginal parts of the platforms throughout the Maastrichtian^39^. The VdP site also largely anticipates the development of postulated smaller islands for the Maastrichtian. Geological and palaeobiological constraints supporting insular settings have been applied to the Hațeg Basin of Romania, where a diverse, Maastrichtian fauna has been reported^15,18,40–48^. Multiple proxies, including the unbalanced composition of the fauna, phylogenetic, ecological, and bone histological evidence, have been applied to document insular characters of taxa, including the hadrosauroid *Telmatosaurus transsylvanicus*^2,49^. Regarding the VdP site, the unique environmental and taphonomic conditions combined with taxa unreported elsewhere, limit inferences on the purported insular conditions of the local dinosaur fauna. Here, we integrate the new biostratigraphic data with analyses of the newly recovered and prepared skeletons of multiple individuals of this taxon to address these historical biogeographic and evolutionary hypotheses.

### Systematic palaeontology

Dinosauria Owen (1842)^50^.

Ornithischia Seeley (1887)^51^.

Ornithopoda Marsh^52^ (1881)^52^.

Hadrosauriformes Sereno (1997)^53^.

Hadrosauroidea Cope (1870)^54^ sensu Madzia et al.^55^.

Hadrosauromorpha Norman (2014)^56^.

*T. insularis* Dalla Vecchia 2009^7^.

### Locality and horizon

Liburnian *facies* (Fig. 1a; Fig. S1) of the Villaggio del Pescatore site (45.8° N, 13.6° E), referred by means of the associated foraminifera and lithostratigraphy to an interval comprised between the lower Campanian and the lowermost middle Campanian (see Geology and revised age of the VdP fossil assemblage section above and Supplementary information S1).

### Revised diagnosis

Here we reformulate the diagnosis for this taxon based on the seven recently discovered articulated skeletons attributed to *Tethyshadros* collected at the type locality. Newly identified unique characters are highlighted with an asterisk (*). *Tethyshadros insularis* is a non-hadrosaurid hadrosauroid dinosaur characterised by the following autapomorphies: proximalmost caudal centra (Fig. S11) are anteroposteriorly longer than dorsoventrally tall, apart from the 3rd and 4th centra*; distal caudal centra transition to becoming more elongated and cylindrical (Figs. S12, S13) in shape halfway through the caudal series (between caudal 23rd–33rd)*; apically broad neural spines in lateral view: haemal arch shape in lateral view vary from rod-like to boot-like to bilobate along the caudal series; flat distal articular end of metacarpals; only two phalanges in manual digit IV, distal one very reduced (lost phalanx 2 of other hadrosauriforms).

In addition to these apomorphic traits**,** *T. insularis* can be further differentiated from other closely related hadrosauroids in the following cranial characters noted here for the first time: thickening of the anterior process of the postorbital less marked than in saurolophine hadrosaurs^57^ and slightly more pronounced than in *Levnesovia*^58^ and *Sirindhorna*^59^. Basitubera round and prominent, like in *Eolambia*^60^ and *Levnesovia*^58^ rather than the more diminutive processes in later diverging hadrosauroids like *Acristavus*^61^, *Brachylophosaurus*^62^, *Edmontosaurus*^57^ and *Parasaurolophus*^63^. Robust exoccipital processes arched caudolaterally, reaching a ventral depth in their distal ends that terminates above the foramen magnum, closer to the condition in *Eotrachodon*^63,64^ and *Edmontosaurus*^57^ rather than to the lower, more ventral extent reached by the exoccipital processes in taxa like *Jintasaurus*^65^, *Levnesovia*^58^, *Eolambia*^60^ and *Parasaurolophus*^63^. Basipterygoid processes dorsoventrally long, anteroposteriorly short and slender with slightly expanded and round articular ends, as in the medially deep basisphenoid recess of *Levnesovia*^58^ but differently from *Edmontosaurus*^57^, *Probrachylophosaurus*^66^ and other hadrosaurids. Two slit-like, ovoidal in shape and approximately equal in size exits for cranial nerves X and XI, comparable to those in earlier diverging hadrosauriforms like *Levnesovia*^58^, but different from the larger and more circular in shape foramina of hadrosaurids^57^. Shallow anteroposteriorly directed ridge running parsagitattally through the dorsal half of the prootic and extending to the exoccipital process, similarly to *Levnesovia*^58^ and *Lophorhothon*^63^ but different from the deeper, thicker process in *Edmontosaurus*^57^.

### Morphological description and ontogenetic variability in *Tethyshadros*

The new articulated skeleton of *T. insularis* SC 57247 (Fig. 2; Figs. SS15, S16) represents a relatively less complete skeleton than the holotype, but it preserves the skull, pelvis, and most of the tail, in addition to fragmentary and isolated material of the appendicular skeleton. Nonetheless, SC 57247 (Fig. 2a; Figs. S5–S7) preserves a complete and articulated neurocranium, laterally exposed on the left side due to the erosion of the jugal and quadratojugal (Fig. 2b, c; see Supplementary Information S2 for a more extensive description).

### Skull

The postorbital in both SC 57021 and SC 57247 (Fig. 2b; Figs. S2, S3) is a tetraradiate bone with a long and slender ventral process, which is concave anteromedially and convex posteromedially. The lateral surface of the postorbital is flat in both specimens, but with an anteroposteriorly wider margin in SC 57247 than in SC 57021. The anterior process of the postorbital is more dorsoventrally thick in SC 57247 (Fig. 2b; Fig. S5) than in SC 57021. The textural surface in the postorbital of SC 57247 is smoother and less pitted than the more rugose dorsolateral portion of SC 57021, and some lateroventral rugosity persists mostly in the ventrolateral portion of the anterior process of the bone (Figs. S5, S7). While the extent of thickening of the anterior process of the postorbital is by no means comparable of the inflated condition seen in some derived hadrosaurids, like *Edmontosaurus*^57^, a similar trend is observable in the ontogenetic series of this taxon. The condition in *Tethyshadros* on the other hand, resembles most directly the slender morphology in *Levnesovia*^58^ and *Sirindhorna*^28^. The basioccipital is rounded caudo-ventrally with a sinuous ventral margin from the occipital condyle caudally to the basitubera anteriorly (Fig. 2b; Fig. S8). Basitubera are round and prominent, like in *Eolambia*^60^ and *Levnesovia*^58^ rather than the more diminutive processes in derived hadrosaurids like *Acristavus*^67^, *Brachylophosaurus*^62^, *Edmontosaurus*^57^, and *Parasaurolophus*^68^. The rather robust exoccipital processes arch caudolaterally, reaching a ventral depth in their distal ends that terminates above the foramen magnum. This condition is closer to those found in taxa like *Eotrachodon*^64^, *Lophorhothon*^63^, and *Edmontosaurus*^57^, and dissimilar to the lower, more ventral extent reached by the exoccipital processes in *Jintasaurus*^65^, *Levnesovia*^58^, *Eolambia*^60^, and *Parasaurolophus*^68^. The basipterygoid processes are dorsoventrally long (Fig. 2b; Fig. S8), anteroposteriorly short and slender until their slightly expanded and round articular ends: this relates to a medially deep basisphenoid recess (like in *Levnesovia* but differently from *Edmontosaurus* and *Probrachylophosaurus*^57,66^). Foramina of the cranial nerves X–XII are placed on the exoccipital-opisthotic complex in a sub-horizontal arrangement, slightly anterodorsally inclined (Fig. 2b; Fig. S8). Two slit-like, ovoidal in shape and approximately equal in size exits for cranial nerves X and XI are presents, with shapes comparable to those in basal hadrosauromorph^55^ like *Levnesovia*^57^, while differ from the more circular shape of later diverging hadrosaurids (like *Edmontosaurus regalis*^57^). There is a shallow anteroposteriorly directed ridge running parasagittaly through the dorsal half of the prootic and extending to the exoccipital process, similarly to *Levnesovia*, *Lophorhothon*^58,63^, and differently from the deeper, thicker process in *Edmontosaurus*. The orbitosphenoid and presphenoid appear fully ossified in SC 57247, housing the cranial nerves I–VI (Fig. 2b; Fig. S8).

### Axial skeleton

Based on the identification of 5 distinct diapophyses in SC 57021, 5 sacral vertebrae are identified in continuity with the posterior dorsal centra, although partially obscured in this anterior area by the preacetabular process of the ilium (see Supplementary Information S2 for a more extensive description). Posteriorly to a fault, two vertebrae are preserved with distinguishable neural spines. We interpret the block posterior to the fault as displaced from the remnant anterior part of the sacrum, making the two vertebrae on the posterior block sacrals 6th and 7th, respectively (contra Dalla Vecchia^7^; Fig. S14). Our interpretation is supported by what is preserved in SC 57247, where four well preserved distalmost sacral vertebrae are aligned and exposed in lateral view in SC 57247 (Fig. 2a; Figs. S9, S15), of which the two distal centra only bear clearly defined and well-preserved neural spines. This re-evaluation brings the total number of sacral vertebrae to 7 (see details in Data S2). The caudal series in SC 57247 (Fig. 2a; Figs. S12, S16) is relatively well preserved (although modified by heavy diagenetic deformation in some intervals) and 43–44 preserved centra are distinguishable (see below and Supplementary Information S2). Based on the absolute size and relative proportions, mainly compared on the axial skeleton (Supplementary Information S2), SC 57247 appears larger than the holotype. Considering the linear dimensions of the skull and overlapping tail elements, and the potential serial discrepancies between the relative position of the centra in the two individuals, we estimate that in SC 57247 is 15–20% larger than SC 57021 (see further details in Supplementary Information S2 and Data S2).

### Ontogenetic stages of *Tethyshadros* specimens

**Figure 3:**
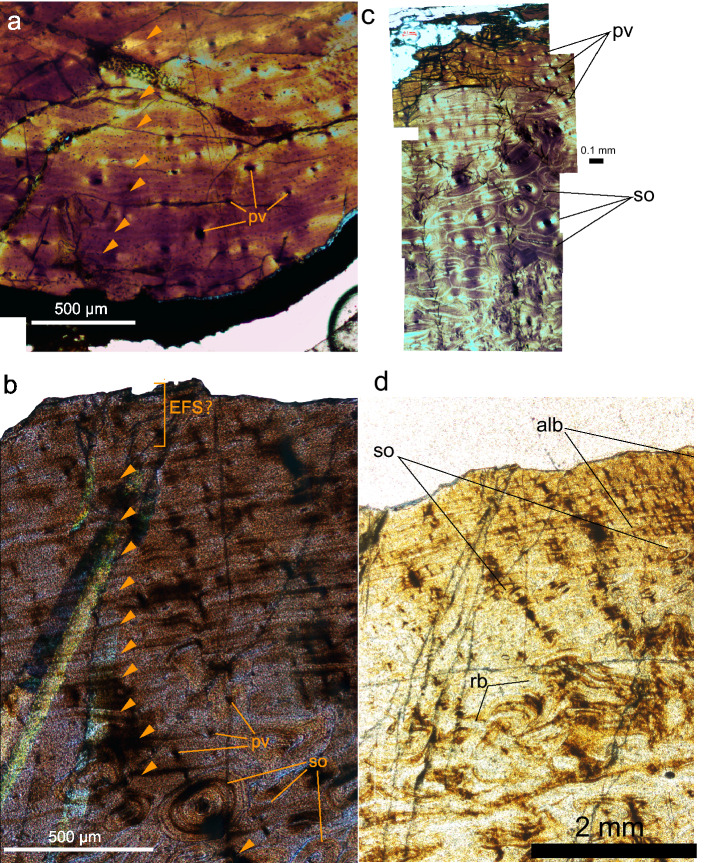
Osteohistology of *Tethyshadros insularis* revealing the somatically immature status of the holotype (**a**) and the older, potentially somatically mature stage attained by SC 57247 (**b**). Count of lines of arrested growth (LAGs^71^) is shown in (**a**) and (**b**), while differences in histological texture organisation between the inner cortex and the outer-peripheral margin of the bone are shown in (**c**) for SC 57021 and in (**d**) for SC 57247. Abbreviations: alb, avascular-laminar to sub-parallel-fibered bone tissue; pv, primary vascularity; rb, remodelled bone tissue; so, secondary osteons; EFS?, tentatively identified External Fundamental System.

The holotype of *Tethyshadros* (SC 57021) was inferred to be a somatically mature individual, based on the fused sutures between vertebral centra and neural spines^7^. However, these morphological proxies were previously suggested to be poorly informative to non-informative^69,70^. We therefore tested this hypothesis of maturity via osteohistological analyses performed on dorsal ribs of SC 57021 and SC 57247 (see methods for reasoning behind our sampling strategy for osteohistological analyses; Fig. 3; Figs. S21–S23; Data S4). The primary cortical bone tissue consists mainly of laminar to sub-parallel–fibered bone tissue. Primary vascular canals are longitudinally oriented and decrease in density towards the outer cortex in both individuals (Fig. 3), becoming absent peripherally only in SC 57247 (Fig. 3b, d). Well-developed secondary osteons forming a Haversian system are abundant in the inner cortex, with at least three and four generations of secondary osteons counted in SC 57021 (Fig. 3a, c) and SC 57247 (Fig. 3b, d), respectively. Eleven Lines of Arrested Growth (LAGs^71^) are found in SC 57021 (Fig. 3a), while at least fourteen can be counted in SC 57247 (Fig. 3b). An External Fundamental System (EFS^72^), here defined on the base of absence of primary vascularization, presence of lamellar zonal bone tissue, and closely spaced LAGs in the outer cortex) is tentatively inferred in SC 57247 (Fig. 3b, d), although the incompleteness of the outer surface opens the possibility that bone lamellae might have been still deposited and subsequently eroded during preparation in SC 57247 (but not in SC 57021, see Fig. S23) or that this histological structure represent, in an asymmetrical bone such as a rib, a case of cortical drift (higher bone apposition on one side compared to the other). While these features still confirm a relatively older age of SC 57247 compared to SC 57021, it is still possible that SC 57247 had not yet completely stopped to grow somatically at the time of its death. Heavy secondary osteon remodelling is also present only in SC 57247, particularly in the inner cortex (Fig. 3b, d). The same area is characterised by more prominent primary osteons in the holotype, SC 57021. Based on decrease in zonation between LAGs observed in the preserved primary cortex (Fig. 3c, d), sexual maturity might have been reached between LAGs 5–7 in the holotype and 4–6 in SC 57427. This does not refer to the absolute age number, but to the earliest visible LAGs in the thin sections: the earliest LAGs were eroded due to remodelling and extrapolation of the number of missing LAGs remains ambiguous due to compression of the bones. Decrease in zonation was previously suggested to correlate in sauropsids with the transition from exponential growth to asymptotic one, and to coincide with achievement of sexual maturity^69,73,74^. These observations show that the holotypic individual and SC 57427 reached sexual maturity 6–4 years and 10–8 years before death, respectively. Based on these observations, we refer SC 57021 and SC 57247 as somatically immature and potentially mature individuals^75^, respectively. The proportional and anatomical differences between these two individuals can therefore best be explained as the result of ontogenetic variation.

### Phylogenetic systematics and body-size evolutionary modelling

**Figure 4:**
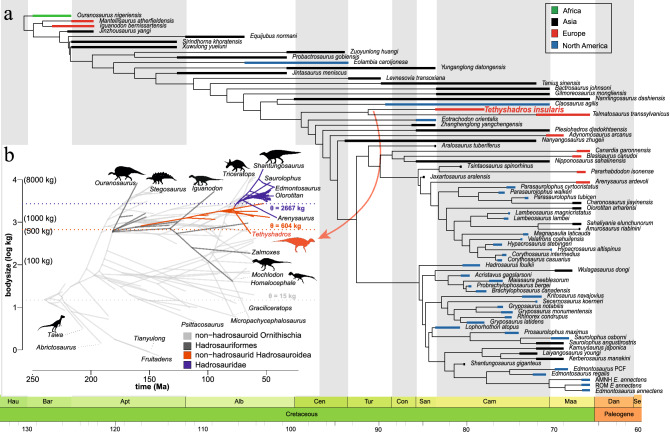
Phylogenetic systematics of *Tethysahdros insularis* showing the biogeographic position of each taxon in the Phylogeny of Hadrosauriformes (**a**). An evolutionary phenogram (**b**) fitting the best OU model (see “Materials and methods”) for the evolution of body size in Ornithischia is also included, highlighting the body-size optima characterizing non-hadrosaurid hadrosauroids (orange), including *Tethyshadros* (red silhouette) and Hadrosauridae (blue). Symbols and abbreviations: θ, optimal body-size; Ma, Mega-annum (million years ago). While data on the y-axis are log scaled, bracketed values represent their equivalent in kg. Silhouette credits: *Tethyshadros*—Marco Muscioni (CC-BY 4.0); *Iguanodon*, *Ouranosaurus*, *Stegosaurus*, *Triceratops*—Scott Hartman/Phylopic (CC-BY-NC-SA 3.0); *Staurikosaurus*—Bruno Navarro/Phylopic (CC-BY-NC 3.0); *Homalocephale*—Michael B. H. (vectorized by T. Michael Keesey)/Phylopic (CC BY 3.0); *Zalmoxes*—Scott Hartman/Phylopic (CC-BY-NC-SA 3.0); *Edmontosaurus*—Matt Dempsey/Phylopic (CC-BY 3.0).

Because previous phylogenetic analyses were based on the immature individual SC 57021, a re-evaluation of the evolutionary relationships and body mass estimation of *Tethyshadros* is needed^76,77^. Our phylogenetic analysis (Fig. 4a; Data S3) recovered 8 most parsimonious trees with best score in step-length of 1177. *Tethyshadros* is nested within Hadrosauroidea (sensu Madzia et al.^55^), as part of that early diverging grade of relatively smaller bodied (body mass > 2700 kg) hadrosauriforms radiating before the more derived, hadrosaurid node characterised by larger sizes (body mass between 1500 and 9000 kg with exceptional outliers, like the Asian *Shantungosaurus* well over 10,000 kg). The strict consensus (Fig. S24) of the taxon-based phylogenetic analyses results in *Tethyshadros* and its sister taxon *Telmatosaurus* bracketed by Asian taxa like *Levnesovia*, *Nanningosaurus*, *Bactrosaurus*, *Zhanghenglong*, *Plesiohadros*, and North American taxa (*Claosaurus* and *Eotrachodon*; Fig. S24). The resulting phylobiogeographical nestedness of *Tethyshadros* between a grade of Asian, early diverging hadrosauromorph taxa, has also been recently recovered in the biogeographic analysis by Kobayashi et al.^78^, McDonald et al.^79^ and partially by Prieto-Marquez and Carrera Farias^80^. When the two most complete individuals (SC 57021 and SC 57247) are scored as different operational taxonomic units (OTUs), they form a clade nested in a grade of non-hadrosaurid Hadrosauroidea (Fig. S25), strongly supporting that both these specimens belong to the same species-level taxon.

Modelling the evolution of body-size in Ornithischia with a focus on hadrosauroids (Fig. 4b) suggests that no forced miniaturisation is required to explain the size of *Tethyshadros* nor of its closely related taxa previously suggested to be dwarfed. The range of body sizes in Hadrosauriformes (hadrosauroids plus iguanodontids) is large, ranging between 40 and 17,000 kg. *Tethyshadros* (514.33–584.37 kg) falls in the middle of this coarse size range (based on the more mature individual SC 57247). Given the large size heterogeneity, we investigated whether smaller, earlier diverging hadrosauroids evolved under different selective regimes than more derived, large ones (Hadrosauridae). Based on AIC values (Table 1), two best performing models (comparable AIC scores; Table 1) are a OUMVA (multi‐peak OU model with both rate of divergence σ and strength of attraction α as free parameters) using 3 partitions (Hadrosauridae, non-hadrosaurid Hadrosauroidea, and non-hadrosauroid ornithischians) or 2 partitions (Hadrosauridae vs all non-hadrosaurid dinosaurs). In the latter model, there is a slowdown in evolutionary rate (σ^2^ = 1 × 10^–9^) towards a stronger attraction (α = 0.05 compared to α = 1 × 10^–9^ outside Hadrosauridae) for a body-size optimum of θ = 1528 kg. In the other (tripartite) suboptimal model (Fig. 4b), we see an incremental slowdown of evolutionary rates in Hadrosauroidea. This relative slowdown in evolutionary rates in non-hadrosaurid hadrosauroids (σ^2^ = 0.1 × 10^–3^ contra σ^2^ = 0.01 outside Hadrosauroidea) is driven by a stronger attraction (α = 0.033 contra α = 0.62 × 10^–4^ outside of this clade) to a body size optimum (θ) of 604 kg. According to this model, Hadrosauridae evolves towards a higher optimal body-size (θ) of 2667 kg with a comparably strong attraction (α = 0.013) than earlier diverging hadrosauroids, but with a noteworthy slowdown in evolutionary rates (σ^2^ = 0.26 × 10^–6^). Testing whether a more inclusive partition with all hadrosauroids instead of only Hadrosauridae fails in finding an equivalently supported OUMVA model (Table 1), with a third suboptimal solution as a BMS model (Brownian motion models with different rate parameters for each state on the tree), which confirms the previously found slowdown in evolutionary rates (σ^2^ = 0.35 × 10^–2^ in Hadrosauroidea contra σ ^2^ = 0.1 × 10^–1^ outside of this clade).

**Table 1:** AIC evaluation of model performance on the different evolutionary models run for best fit on the Phylogeny of Ornithischia updated to the topology recovered in this study and the new body size data reported herein for *Tethyshadros*.

OU model	AICc	AICc weights
OUMVA clade 2pDae	202.10121026452	0.5365
OUMVA clade 3p	203.316849995322	0.2922
BMS clade 2pOidea	206.480134133426	0.0601
BMS clade 2pDae	207.27392334102	0.0404
BMS clade 3p	208.690134835209	0.0199
OUMV clade 2pOidea	209.476798399784	0.0134
OUMV clade 3p	209.592599479123	0.0127
OUMVA clade 2pOidea	210.558649385763	0.0078
OUMV clade 2pDae	210.885190749501	0.0066
OUMA clade 2pDae	214.44485372578	0.0011
EB 2pDae	215.0879216843	0.0008
EB 2pOidea	215.087921684731	0.0008
EB 3p	215.087921685969	0.0008
BM 3p	215.782901747963	0.0006
BM 2pOidea	215.782901747963	0.0006
BM 2pDae	215.782901747963	0.0006
BM clade 3p	215.782903511791	0.0006
BM clade 2pOidea	215.782903511791	0.0006
BM clade 2pDae	215.782903511791	0.0006
OUM slice 3p	217.690892667324	0.0002
OUM slice 2pOidea	217.690892667324	0.0002
OUM slice 2pDae	217.690892667324	0.0002
BM slice 3p	217.701629604779	0.0002
BM slice 2pOidea	217.701629604779	0.0002
BM slice 2pDae	217.701629604779	0.0002
OUMA clade 3p	217.811129235353	0.0002
Stasis 2pOidea	217.88206501181	0.0002
Stasis 2pDae	217.88206501181	0.0002
OUM clade 2pDae	218.212527872988	0.0002
OUMA clade 2pOidea	219.102630618848	0.0001
OUMA slice 3p	219.219294813277	0.0001
OUMA slice 2pOidea	219.219294813277	0.0001
OUMA slice 2pDae	219.219294813277	0.0001
OUMV slice 3p	219.315502770849	0.0001
OUMV slice 2pOidea	219.315502770849	0.0001
OUMV slice 2pDae	219.315502770849	0.0001
OUM clade 2pOidea	219.454474618386	0.0001
Stasis 3p	219.926245193174	0.0001
Trend 3p	219.926245193174	0.0001
Trend 2pOidea	219.926245193174	0.0001
Trend 2pDae	219.926245193174	0.0001
OUM clade 3p	220.367158236624	0.0001
OUMVA slice 3p	221.419942633016	0
OUMVA slice 2pOidea	221.419942633016	0
OUMVA slice 2pDae	221.419942633016	0

Symbols and abbreviations: BM, Brownian motion; clade, models with different parameters for each clade; EB, early boost (high σ at the base of the tree); slice, a model with a significant shift at a given time slice, here selected at 93.9 million years ago (Cenomanian/Turonian boundary); Stasis, a model with strong α towards a given θ; OU, Ornstein–Uhlenbeck; OUM, multi‐peak OU model (multiple regimes within individual θ values) with fixed values of α and σ; OUMV, multi‐peak OU model with fixed values of α and σ as a free parameter; OUMA, multi‐peak OU model with fixed values of σ and α as a free parameter; OUMVA, multi‐peak OU model with both σ and α as free parameters; Trend, a model following a given linear trend (given α constant). Partitioned models are abbreviated as: 3p, three portioned model with Hadrosauridae, Hadrosauroidea and non-hadrosauroid Ornithischia; 2pDae, two partitioned model with Hadrosauridae and non-hadrosaurid Ornithischia; 2pOidea, two partitioned model with Hadrosauroidea and non-hadrosauroid Ornithischia.

## Discussion

### Re-evaluation of *Tethyshadros* palaeobiology

*T. insularis*, previously known only from an ontogenetically immature individual, is here revised as more closely fitting the (more average) standard size of non-hadrosaurid hadrosauroids. Although diminutive in absolute mature size, *Tethyshadros* is within the range of a widely diverse grade of hadrosauroids clustering near the base of Hadrosauridae. Furthermore, the development of several maturity-related characters such as more robust cranial traits and stouter proportions, strengthens a relationship of this taxon with other hadrosauroids of Asian origins. While our analysis strongly supports this classic paraphyletic assemblage of non-hadrosaurid hadrosauroids (Fig. 4), some recent analyses have reconstructed a clade of Eurasian taxa comprising *Tethyshadros* and *Telmatosaurus*^79^. Although the differences in taxon-inclusion and procedural settings may explain some minor discrepancies in the results of these phylogenetic analyses, it is possible that a better sampling of non-hadrosaurid hadrosauroids from the Cretaceous of Eurasia may reveal an earlier, more diverse, and geographically widespread radiation of hadrosauroids than previously appreciated. The differential survival of larger-sized hadrosauroids up to the end of the Cretaceous in Asiamerica might hint to a selective extinction of diminutive hadrosauroids, or, most likely, be driven by undersampling of smaller sized-range hadrosauriforms. Members of this group might have reached European landmasses through several biogeographic connections, like highlighted in this study by a purportedly more extensive AdCP than currently inferred (Fig. 1c). Our results support a multiphase biogeographic dispersion of hadrosauroids from Asia into Europe (Cenomanian–Turonian) involving the descendants of many hadrosauroids outside of Hadrosauridae^58^, followed by a post-Santonian radiation restricted to more derived Lambeosaurinae reaching the Iberian plateau^62,81^ and Northern Africa^82^.

Regarding the AdCP, several authors recognize consecutive biogeographic scenarios for the Late Cretaceous characterized by the loss of Gondwana-AdCP connections in favour of AdCP-Eurasia links during the latest Cretaceous^1,6,42^. Given its carbonate-dominated, marginal marine settings, the AdCP also differs enormously in terms of palaeobiogeographical settings from coeval landmasses in the palaeo-Mediterranean areas, in particular the Ibero-Provencal areas. The body-size reconstructed for SC 57247 suggests it represents a 20% larger individual than the holotype based on skull and tail length (Data S1). The skull of SC 57247 with its remarkably shorter and brachyrostrine proportions, more closely resemble non-hadrosaurid grade hadrosauroids rather than hadrosaurids. The ostohistological analyses indicate different ontogenetic stages, with a smaller, immature individual and the stouter, larger one as an older individual, and potentially somatically mature. Based on these results, our study brings a novel apomorphic diagnosis defining *Tethyshadros* based on the only known adult individual of this taxon, SC 57247. After the ontogenetic reinterpretation of the holotype as pertaining to an immature individual through means of osteohistology, many of the anatomical features previously considered aberrant, are now reconsidered as related to ontogeny. The lack of peculiar morphological adaptations, from a tail fully reflective of a ‘basal hadrosauroid’ condition to a body size in range with that of such taxa erode the set of peculiar features justifying unique adaptations to an insular, isolated setting (contra Dalla Vecchia^7^). Our phylogenetic comparative approach shows that there is no signal of overall miniaturization in any of the considered partitions of Hadrosauriformes. If a multimodal shift in rates and pursuits of adaptive landscapes is considered (OUMVA models), successive optimal body-sizes rise, rather than decreasing, such as specifically shown in Hadrosauridae (Fig. 4b). This general body-size increase in hadrosauriforms coincides also with a slowdown in evolutionary rates, which might be explained by the denser adaptive landscape filling by Hadrosauridae around 2000 kg, but might also be due to size-selective extinction (depending on the time terminal branches of non-hadrosaurid hadrosauroids end) or size-dependant sampling bias^83^. In addition, size disparity appears greater in earlier diverging hadrosauroids rather than in Hadrosauridae, and that might explain our perceived predisposition to consider everything not fitting the multi-ton size of latest Cretaceous hadrosaurids as a “dwarfed animal”. Together, this information implies a slower evolution towards specific body-size adaptive landscapes, possibly since their primary evolutionary niche might have been following other phenotypic dimensions (like for example trophic ecology^84,85^) or might have depended by specific biomechanical constraints^21,86^.

The morphological and systematic affinities of *Tethyshadros* with earlier diverging hadrosauroids from Asia is strengthened by the chronostratigraphic data presented here. Given the higher terrigenous influence and larger area of the AdCP inferred here, a higher faunal flux in this area between Asia and Gondwana becomes an increasingly more relevant phenomenon than previously considered. *Tethyshadros* and its assemblage from VdP with its unique chronostratigraphic setting would represent the evidence of one of these dispersal events, where at least the northern margin of the proto-Italian microplate assemblages hosted a diverse fauna of Asian origins. With the new evidence provided herein, we confute the presence of the co-set of geographic and environmental constraints necessary to trigger evolutionary modes towards insular dwarfism^2,87^, analogous to those reconstructed for the Maastrichtian Hațeg domain. The earlier dating coincided with a time of less extensive fragmentation of this region, and the higher connectivity with the wider, continental Asian domain is indicative of potentially higher biotic interchange, if not vicariance with the Asian biogeographic province.

With approximately 400 still undescribed specimens, including vertebrates, invertebrates, and plant remains, the VdP site represents a unique opportunity to understand peri-Tethyan biogeographic dynamics, the sole for the Campanian. However, without the stronger, multi-disciplinary set of evidence presented here, inference of unique and peculiar palaeobiological patterns promptly dissolves into a more nuanced, nevertheless interesting picture of the biogeographic history of this area. The multi-individual sample and partial ontogenetic series for *Tethyshadros* presented herein provide new information that cast new light on its evolutionary history. The VdP site will continue to provide a valuable record of the complex history of exchanging biota from the two main Mesozoic landmasses in the Eastern hemisphere, revealing fundamental insights into palaeobiological history of this poorly sampled region from the predominantly North-American dominated record of the Late Cretaceous.

## Materials and methods

### Osteohistology

The multiple individuals of *Tethyshadros* show proportional and anatomical differences associated to different body size, which are most evident between the two articulated skeletons SC 57021 (“Antonio”) and SC 57247 (“Bruno”). We performed osteohistological investigation on these two specimens to assess their ontogenetic stage, explain the morphological differences between them, and to contribute revising the body mass estimates for this taxon. Although invasive, it was recently suggested that osteohistology is the most reliable approach to assess the ontogenetic stage and growth strategies in extinct taxa^69,70,88^. We sampled the 7th and 13th dorsal ribs of SC 57021 and SC 57247, respectively (Fig. S21). The discrepancy between the serial identity of the sampled ribs is due to taphonomic reasons: the 7th and 13th ribs were already fractured, facilitating invasive sampling, and minimizing harm to the specimens. Because of serial homology, the different dorsal ribs sampled for this study are still comparable for the aim of ontogenetic assessment of the two individuals. Although not extensively validated for studies of vertebrate skeletochronology, dorsal ribs were chosen above other skeletal elements, as they were previously recognized to be remarkably informative for assessment of ontogenetic stages^73,74,88^ and because preserved in both specimens, contrary to the appendicular skeleton that is mostly missing in SC 57247; moreover, dorsal ribs are phylogenetically less informative than other postcranial elements, such as long bone, allowing to lose the lowest amount of anatomical information. The dorsal ribs were sampled proximally (Fig. 3; Figs. S21–S23; Data S4), the region of the bone where the most complete growth signal is recorded^74^. Thin sections were prepared following the protocol described in Chinsamy and Raath^89^ and brought to a thickness of 90 microns. Thin sections were investigated with a Leica DM 2500 P petrographic microscope and pictures were taken with a ProgRes CFscan camera attachment. Images used in Fig. 3 were taken with crossed nicols (a, b) to enhance visibility of the bone cellular organisation system, while those in Fig. 3c, d were taken with parallel nicols to maximise contrast and highlight textural differences between the inner portion and peripheral cortex. We used presence or absence of an External Fundamental System (EFS, sensu Horner et al.^73^) as main criterion to determine somatic maturity (following Fabbri et al.^88,90^). When an EFS was absent, we used zonation between Lines of Arrested Growth (LAGs), density of primary vascularization, and progression of cortical bone remodelling as a relative measure of maturity (Figs. S21–S22). When annuli were encountered (two–three closely spaced LAGs), these were counted as a single year (Figs. S21–S22).

### Phylogenetic systematics and body-size evolutionary modelling

In order to test whether the phylogenetic affinities of *T. insularis* would be consistent with previous findings^7,91^ after ontogenetically dependant re-scoring based on SC 57247 (Data S3), we performed two different maximum parsimony analyses. In the first one (Fig. S24), we scored two separated Operational Taxonomic Units (OTUs) based on SC 57201 and 57247 in a recent phylogenetic analysis of iguanodontian dinosaurs^91^. We then used a single Operational Taxonomic Unit which includes scorings based on SC 57021, SC 57026, and SC 57247 (Data S2). All phylogenetic analyses were performed using a ‘New Technology’ search, with Sect Search, Ratchet, Drift, and Tree Fusing algorithms, and 10 random addition sequences. After this preliminary search, Traditional Tree Bisection-Reconstruction (TBR) Branch-Swapping was then performed on trees held in RAM, as this approach has been shown to provide a more complete exploration of tree space^92^. Following the original iteration of this analysis^91^, we performed a traditional search with 1000 replicates of Wagner trees (with random additional sequences followed by the TBR branch swapping holding 1000 trees per replicate). To test nodal support, we performed both bootstrap resampling using standard absolute frequencies (for 1000 replicates) and calculation of Bremer decay index^91^. In the individuals-based phylogenetic analyses we recovered 11 trees of 1178 steps in length (Fig. S25). The taxon-based analysis recovered 8 trees with best score in step-length of 1177. The strict consensus of 36 trees (Fig. S25) obtained after TBR branch swapping was time calibrated following stratigraphic ranges (Data S2). Finally, in order to compare the effect of weighting on the obtained tree topology, we used four different weighting schemes, an approach first developed by Goloboff^93^: equal weighting and extended implied weighting using k-values of 4, 8 and 12. This procedure is applied to downweigh characters in relation to their average homoplasy whilst minimizing the potential impact of missing data, with a more severe downweigh due to lower k-values^94–97^. The three k-values presented herein were selected following the procedural steps in other palaeontological studies^98–100^. We could observe no effect on tree topology of this weighting tests, confirming tree stability in these phylogenetic results. Time-calibrated tree shown in Fig. 4 was obtained by calibrating the stratigraphic ranges of the tips using the R package strap by Bell and Lloyd^101^ and changing the colours of the tips by continental location. Stratigraphic ranges were updated to the geological setting reported in this study and what is reported on the other taxa in the literature (Data S3).

In order to test whether any significant miniaturisation happened in the lineage leading to *Tethyshadros*, justifying at least solely on a phylogenetic ground insular dwarfism in this taxon, we compared evolutionary models of Ornithischia body-size evolution by means of Ornstein–Uhlenbeck (OU) dynamics^102–104^ integrating the novel information (this study) on the mature size of this taxon. This approach has the advantage of testing several constrained evolutionary scenarios (like the Brownian motion or the Early burst model of evolution) with multi‐peak OU models, which can fit multiple evolutionary changes (several shifts in rate and directionality towards optimal peaks of evolutionary landscapes with heterogenous attraction) for different lineages across a phylogeny. As stated in Benson et al.^21^, implementing this framework has the advantage of testing whether directionality in the filling of a given or multiple adaptive landscapes for a continuous character (like body mass) can reflect a general tendency of the whole clade or is reflecting a general evolutionary tendency across multiple and/or specific lineages. After updating the body size data for *Tethyshadros* according from the new information accessed via SC 57247 (Supplementary information S4), and adapting the original tree topology of Ornithischia to our findings (Figs. S26, S27) we re-run the macroevolutionary modelling of body size from Benson et al.^21^ which uses Ornstein–Uhlenbeck (Hansen) models to test whether the evolution of a trait value (body size, in this case) spread stochastically over time by diffusion of lineages (according to Brownian Motion^24^) or whether it follows a macroevolutionary trait optimum (θ) with a given strength of attraction (α) at a given node (OU model) or at multiple nodes (OUV multiple regimes^102,104,105^. We performed 3 different experiments, running OU modelling with different partitions at each iteration: one in which 3 partitions in the Ornithischian tree were incorporated, with Hadrosauridae (*Maiasaura* + *Corythosaurus*), Hadrosauroidea (*Jinzhousaurus* + *Corythosaurus*) and the non-hadrosauroid taxa treated as different partitioning points in the tree. A second iteration included Hadrosauridae (*Brachylophosaurus* + *Corythosaurus*) and non-hadrosaurid taxa and a third experiment included two partitions represented by Hadrosauroidea (*Jinzhousaurus* + *Corythosaurus*) and all the other taxa. Additionally, we tested whether a significant shift might have happened at the Cenomanian/Turonian boundary (93.9 Ma) affecting any change in evolutionary mode for Ornithischia, since this time interval coincides with strong and documented eustatic and atmospheric changes which might have affected faunal biogeography, particularly in the Tethyan domain^106^ and is also followed by the evolutionary appearance of Hadrosauridae^58,107^ (Fig. 4). Model performance was compared (Table 1) by using the Akaike Information Criterion with finite correction^108^ and AIC weights^109^.

We performed ancestral state reconstruction of body size as a continuous character which was constructed in phytools v.0.4-60^110^ using a stochastic map of 10,000 generations and the ‘SYM’ (Symmetrical) model of evolution on the time-scaled consensus tree (following Gates et al.^111^; Fig. S28). We represented the ancestral state reconstruction as a density map on the phylogenetic tree^112^ of Ornithischia (Fig. S28). We further used GEIGER-fitted comparative model of continuous data^113^ to reconstruct the ancestral state z_0_ (root value) for the base of Ornithischia, Hadrosauriformes, Hadrosauroidea, and Hadrosauridae. We then followed the same parametrisation for OU-analysis reported in Benson et al.^21^ and updated the body-size data modified the tree topology of Ornithischia used in that study to our current results (Fig. 4; Figs. S26, S27). Our modified Ornstein–Uhlenbeck models analysis of continuous trait evolution under selective regimes^102^ was performed using R version 4.0.3 and the package OUwie v.3.5^104^.

## Supplementary Information

Supplementary Information 1.

Supplementary Information 2.

Supplementary Information 3.

Supplementary Information 4.

## Data Availability

The authors declare that all the data supporting the findings of this study are available within the paper and its Supplementary Information files.
