# Supplementary material for: An Italian dinosaur Lagerstätte reveals the tempo and mode of hadrosauriform body size evolution
Source: Sci Rep. 2021 Dec 2;11:23295. doi: 10.1038/s41598-021-02490-x (PMC8640049; doi:10.1038/s41598-021-02490-x)
Supplement: Supplementary file 1 — Supplementary Information 1. [file 41598_2021_2490_MOESM1_ESM.docx]

**An Italian dinosaur Lagerstätte reveals the tempo and mode of hadrosauriform body size evolution**

Alfio Alessandro Chiarenza, Matteo Fabbri, Lorenzo Consorti, Marco Muscioni, David C. Evans, Juan L. Cantalapiedra, Federico Fanti

SUPPLEMENTARY INFORMATION

**Contents**

**S.1.** **Stratigraphic context, chronostratigraphic and palaeoenvironmental revision**

The Upper Cretaceous succession (Fig. S1) of the Villaggio del Pescatore (VdP) site belonged to a Tethyan paleogeographic landmass referred to as Adriatic Carbonate Platform (AdCP) *sensu* Vlahović et al.^1^. This vast element, mostly composed of shallow-water marine carbonates, extended through the Mesozoic for hundreds of kilometres, embracing the areas nowadays included in most parts of north-eastern Italy, Slovenia, Croatia, Monte Negro, and further portions of Serbia and Albania, up to the Ionian Greece^1–3^. The northern limb of AdCP is also referred to as the Friuli Platform, which was surrounded during the Mesozoic by the Slovenian basin to the north-east and Belluno basin to the west^4^. Geographically, in the Trieste coastal limb and the adjacent area of Slovenian, these rocks are referred as belonging to the ‘Karst’.

The Upper Cretaceous to lowermost Paleocene succession of the Karst is comprised by two informal lithostratigraphic units: The *Aurisina* formation (Upper Cenomanian to Campanian pro parte [p.p.]) and the K/Pg bearing *Liburnian* formation (Campanian p.p.–Danian), or by their equivalent synonyms along the adjacent Slovenian units (*see* Jurkovšek et al.^5^). Although interested by subaerial expositions or erosive processes of different durations, the Cretaceous stratigraphy of the Karst area shows a comprehensive record in which most of the stratigraphically relevant bioevents, mainly recorded by benthic foraminifera and rudists, can be straightforwardly correlated with those observed elsewhere in the peri-Mediterranean area^6–10^. At the VdP site, the stratigraphic framework includes the upper part of the *Aurisina* (indicated as ‘*Aurisina* facies’ in Fig. S1) and the lower part of the *Liburnian* facies (indicated as ‘*Liburnian* facies’ in Fig. S1) which lies on the previous one through the interposition of a palaeokarstified surfaces^11,12^ mostly represented at VdP by breccia. The *Aurisina* facies is comprised of whitish limestones, characterised by a texture ranging from wackestone to grainstone, rich in rudists, benthic foraminifera and subordinate cyanobacterial remains densely packed in few layers, representing an open, peritidal, shallow-water marine environment. The *Liburnian* facies is mostly characterized by beige to dark wackestone facies with small rudists (Hippuritacea: Requienidae), a low-diverse benthic foraminiferal assemblage, ostracods, charophytes and green algae remains, representing a paralic, very shallow restricted environment, influenced by both marine and freshwater inputs. Locally, the *Liburnian* facies is represented by dark to beige organic-rich carbonate rhythmites (such as at the VdP) with variable amount of limestone breccia both coming from the erosion of the underlying *Aurisina* facies or from a cannibalized age-equivalent *Liburnian* succession.

**Revised biostratigraphy and age**

The biostratigraphic framework we applied in this study is mainly based on the vertical distribution of benthic foraminifera. Similar to what has been established in the Shallow Benthic Zones of the Paleogene (SBZ^13,14^), the Mesozoic biozonation of shallow-water carbonate platforms is made up of assemblage zones that integrates parallel index taxa^7,15–17^. The existing biozonation proposed for the peri-Mediterranean Upper Cretaceous platforms (*see* e.g.^6,10,18–21^) is indicative, being accurate in the relative position of the bioevents, but not anchored to the chronostratigraphic scale. However, this problem has been mostly solved as the vertical distribution of several Late Cretaceous index taxa is now better calibrated to the chronostratigraphic scale by means of Strontium Isotope Stratigraphy (SIS^7,8,22–24^). The resolution covers all the sediments deposited in the photic zone in tropical to subtropical waters under oligotrophic to mesotrophic conditions and is enhanced by the occurrence of composite zones based on successive assemblages of index fossils with approximately synchronous ranges (Oppelzones^14^). The biozones correspond to a total range of one or two index taxa and are defined using integrated evidence of multiple first appearances (FA) and last occurrences (LO) of taxa both referring to their generic or specific taxonomic ranking^17^.

By studying the records of the most important index taxa collected from the drilled core S3 described in Palci^25^ and currently housed at the University of Trieste (Fig. S2), the base of the *Aurisina* facies show the occurrence of *Pilatorotalia* sp., whereas in the middle part *Keramosphaerina tergestina* (Stache) occurs^26^. Throughout the entire recovered portion referred to the *Aurisina* facies we also report a relatively continuous occurrence of *Rotalispira maxima* Consorti, Caus & Frijia, *Accordiella conica* (Farinacci), *Scandonea mediterranea* De Castro and *Dicyclina schlumbergeri* Munier-Chalmas. The uppermost part of the *Aurisina* facies from the core shows additional occurrences such as *Calveziconus* cf. *lecalvezae* Caus, *Fleuryana adriatica* De Castro et al., *Reticulinella fleuryi* Cvetko, Gušić & Schroeder, *Goupillaudina* cf. *daguini* Marie, *Neorotalia*? *cretacea* Consorti Caus & Frijia, *Murciella* gr. *cuvillieri* and a form most likely referable to *Metacuvillierinella* Fleury & Özkan. These data are integrated with those recovered from our survey performed in the surrounding of the VdP in which the *Aurisina* facies crops out and where we have found *Murgella lata* Luperto Sinni, *Rotalispira scarsellai* (Torre) and some specimens referable to *Neorotalia*? cf. *cretacea*. The rhythmites referred to the *Liburnian* facies lack important benthic foraminifera. However, in the upper portion of the succession at the VdP site we have recovered *Dicyclina schlumbergeri*, *Rotalispira scarsellai*, *Murciella* gr. *cuvillieri*, *Accordiella conica*, *Pseudocyclammina* sp., *Pilatorotalia pignattii* and *Rotalispira* cf. *maxima*, both occurring within a coarse carbonate clastic layer intercalated within the rhythmites (Fig. S2o, r, S3h) and inside a limestone clast (Fig. S3a–g). We interpret the *A. conica* and *Murciella* gr. *cuvillieri* as reworked into the rhythmites from a near shore environment in which they were deposited soon after *post*-*mortem*. Similarly, the foraminiferal-rich limestone clast is interpreted as coming from a cannibalized succession deposited sub-isochronously to the living foraminiferal assemblage and, in turn, to the deposition of rhythmites, thus making these occurrences a further useful constraint.

Based on SIS-anchored biostratigraphy, the entire portion referred to the *Aurisina* facies can be placed into the *Accordiella conica* and *Rotalispira scarsellai* biozone of Chiocchini et al.^6,19^ and Frijia et al.^7^. In particular, the occurrence of *Reticulinella fleuryi*, *Scandonea* *mediterranea* and *Keramosphaerina tergestina* would narrow the chronostratigraphic position into the lower Campanian^7,8,23^. This outcome is in agreement with data coming from the field survey in which *Murgella lata* and *Rotalispira scarsellai* were recovered, both species that can be referred to the lower Campanian^7,23^. The occurrence of *Calveziconus* cf. *lecalvezae* in the topmost *Aurisina* facies adds some clues indicating that the chronostratigraphic range could get close to the lower-middle Campanian boundary^27^, as suggested by Frijia et al.^7^ for the *C.* cf. *lecalvezae* subzone. However, in the AdCP domain, the SIS-calibrated stratigraphic range of *C. lecalvezae* is suggested to be restricted to the lower Campanian^8^. Based on these records, we refer the whole accompanying fauna composed by *Goupillaudina* cf. *daguini*, *Neorotalia*? cf. *cretacea*, *Murciella* gr. *cuvillieri*, *Fleuryana adriatica* and *Metacuvillierinella* sp. to the lower Campanian as well. Even if not precisely constrained in their lower and upper occurrences, the concomitant range of these species considers zonal markers and SIS-anchored records, placing the age into the lower Campanian, and possibly around the lower-mid Campanian boundary^7,8,18,20,21,28^.

The recovery of *Murciella* gr. *cuvillieri* in the upper portion of rhythmites deserves additional consideration as this rhapydioninid foraminifer was widely used to infer the age of VdP fossil fauna^29–31^. The *Murciella* populations that belong to the biogeographic region of the Upper Cretaceous AdCP show a quite wide morphological variability, leading authoritative authors^18,20,32^ to first group all the morphotypes referred to the genus ‘*Murciella*’ (also referred to as ‘*cosinas’* by Bignot^33^) into a single genus, and then to split all the forms recovered along the Campanian and Maastrichtian into different genera by relevant shell differences. The vertical trend in shell morphology seems showing an increase in complexity throughout the Campanian–Maastrichtian interval, mostly related to the growth in number of medullar and cortical chamberlets, thickness of the basal layer, chambers arrangement, presence/absence of relevant skeletal elements as well as general dimension and proportions among the shell compartments. Other larger and more complex rhapydioninids with marked dimorphism finally arose during the Maastrichtian, and are represented by well-established genera, which are chronostratigraphically anchored by SIS^24^. By shell features, our morphotypes can be assigned to the genus *Murciella*, and less inclusively assigned to *M*. gr. *cuvillieri*, an artificial taxon including all the possible morphological variabilities observed into the *M. cuvillieri* type-population of the Campanian of Murcia Province in Spain^34,35^. Furthermore, the SIS-calibrated Upper Cretaceous stratigraphy of the Apulian platform^22,36^, adjacent to the AdCP, reports a foraminiferal assemblage with *Murciella cuvillieri* into the early-?mid Campanian. On the basis of these insights and taking into account the quite low shell complexity of the specimens that we have recovered (Fig. S2i, m, o, r), we believe that all our taxa, and those figured by Dalla Vecchia^37^ and Palci^25^, represent an ancestral *M.* gr. *cuvillieri* population. Based on the AdCP records^8,18^, and particularly into the VdP, the chronostratigraphic range of these individuals would fall between the *Scandonea mediterranea* and *Calveziconus* cf. *lecalvezae* subzones of Frijia et al.^7^, or between the time-equivalent CsB5 and CsB6a biozones established by Fleury^18^. This constraint is supported by the synchronous occurrence in the uppermost portion of rhythmites (Fig. S2p) of specimens referable to *Rotalispira* cf. *maxima* together with *Murciella* gr. *cuvillieri*. The further retrieval of reworked isolated *Accordiella conica* in that stratigraphic position (Fig. S3h) strengthens such age correlation. Similarly, the finding of *Pilatorotalia pignattii* incorporated in a clast together with *Dicyclina schlumbergeri*, *Rotalispira maxima*, *Scandonea mediterranea*, *Pseudocyclammina* sp. and *Accordiella conica* is in accordance with the proposed age^23^ supporting a stratigraphic range through the Campanian as well, into the upper part of the *Accordiella conica* and *Rotalispira scarsellai* biozone.

The biostratigraphy applied here is supported by a new dataset, which comprises taxa either never recorded at VdP or not discussed in detail in previous works. These occurrences help to further constrain the chronostratigraphy of the site, in particular revising the age previously given to the fossil-bearing strata on the sole basis of the foraminifer *Murciella*. Former authors^29,37,38^ have applied a bio-microfacies methodology to study the site, that not necessarily takes into consideration the chronostratigraphic range of age-controlled shallow-water biozones but also a quite pure regional lithostratigraphic correlation in which the distribution of the benthic fauna may be environmentally-biased. Based on our approach that considers both SIS-anchored records and multiple occurrences of synchronous zonal markers, the entire VdP succession can be thus protracted into the upper part of the lower Campanian and the lower part of the middle Campanian, likely within a time interval of roughly 1 My, comprised between 81.5 Ma and 80.5 Ma *sensu* Frijia et al.^7^. Although we agree with former authors that the magnitude of subaerial exposures affecting the top of *Aurisina* facies are difficulty to estimate in detail and to correlate regionally^37^, our data at VdP suggest that duration of non-deposition prior to the rhythmite accumulation was relatively short in contrast to other AdCP areas (*see* Vlahović et al.^1^). This gap, commonly linked to paleotectonic movements (Otoničar, 2007), is roughly comparable to the Campanian hiatus recorded by Steuber et al.^8^ in Dalmatia, and suggests a narrower duration than previously established for the Karst^5,39,40^. Finally, the rhythmites deposition unquestionably took place in a geologically instantaneous time span (few thousands of years, *see* Arbulla et al.^41^) that, even comprised within the proposed time range, goes beyond the available resolution.

**The Villaggio del Pescatore site – quarry map, lithostratigraphy and stratigraphic distribution of fossil remains.**

The Villaggio del Pescatore site (VdP) was discovered about thirty years ago in the Campanian–Paleocene beds exposed west of Trieste, north-eastern Italy^41,42^. Ever since, both the geology of the area and collected fossil remains have been discussed in a series of scientific reports, but surprisingly a common ground on the real importance of the site in its entirety has not been achieved. The overall geological setting of the site and neighbouring areas has been described in several works^12,25,26,29,37,41,43,44^ and enhanced in recent years by unpublished GIS and photogrammetric campaigns. We emphasize that data presented in the literature describing the VdP site refers exclusively to a very restricted area of approximately 300 m^2^ where extractive processes related to the collection of fossil remains resulted in a polished, ground level where most of geologic observations were made. Therefore, only 40% of the site has been properly documented, and available data support extension of fossil beds outside the main, currently protected area.

From a sedimentologic perspective, the uniqueness of the site is represented by sharp facies variations from open marine, shallow-water limestones to organic-rich rhythmites, which interbed with breccias that accumulated as underwater bodies of breccia by underwater, density-driven, sedimentary flows (Fig. 1; Fig S1). These slumped, organic-rich rhythmites not only preserve all fossil remains but are responsible of the plastic folding of the large fossil vertebrates, a setting with no equivalent in the literature nor in any Mediterranean Mesozoic carbonate platforms^1^. Slumps also raise questions concerning the taphonomy (preservation of land vertebrates into dysoxic to anoxic bottom waters in marginal-marine settings) as well as the interaction between different depositional settings (terrestrial–shallow marine).

Overall, six lithofacies have been distinguished based on lithologic, sedimentologic and paleontological characters, although their complex, reciprocal architecture remains poorly documented^41^. Fossils are confined within a ~10 meters thick, slumped, organic-rich rhythmites arguably deposited within a restricted basin. Each identifiable lamina consists in a micro-couplet made by a mm-thick dark, organic-rich lamina superimposed to a lighter lamina made of carbonate mud thicker than the previous one. Given an overall thickness of ~10 m and a thickness of each couplet ranging from 1 to 2.5 cm, the ‘*laminites*’ lens was estimated to represent a time interval of 4000-10000 years^41^. However, this estimate did not consider multiple slumps occurring within the rhythmites interval that locally duplicate (or even triplicate) the occurrence of discrete intervals. The laminites have yielded remains of *Tethyshadros insularis*, a partial theropod metatarsal, small crocodyliforms (*Acynodon*^45^), a single pterosaur bone, partial fishes, several crustaceans (large shrimps and many small shrimp-like crustaceans), and rare coprolites (*see* Dalla Vecchia^46^) and references therein). Fish are the most abundant vertebrates representing more than 50% of all identifiable fossils. Plant remains are rare, although palynological investigations have revealed the presence of abundant organic matter consisting of unstructured sheets, palynomorphs, multicellular filaments and spores^41^. A pilot project related to the organization of historic, neglected, and new data into a unified framework carried out between 2019 and 2020 resulted in important and unforeseen outcomes related to the Villaggio del Pescatore area. In fact, with more than 450 collected fossils, more than 100 still unprepared specimens and others still in situ cropping out at the main site, and complete high-quality, continuous cores which also resulted in about 200 thin sections, the VdP offers a unique opportunity for high-resolution, qualitative and quantitative analyses of the site.

Data collected in more than 30 years of excavation at the main site (including scientific publications, reports, photos, geological sketches, personal information) were gathered using GIS software to produce the most accurate and up-to-date quarry map for the VdP site. This process was essential in re-locating specimens lacking detailed quarry information as well as newly collected and still *in situ* specimens into a comprehensive framework. The complete inventory of the VdP site is deposited at the Museo di Storia Naturale in Trieste. Quarry map shown in Fig. S4 includes 1) location of cores collected in 2002, described in Palci^25^ and currently stored at the University of Trieste; 2) orthophoto of the polished surface of the main quarry area with superimposed the original progressive numeration of limestone block removed from the quarry in the 1990’s (*see* also Dalla Vecchia^47^); 3) precise location of specimens currently housed at the Museo di Storia Naturale di Trieste (MCSNTs); 4) precise location of *in situ* specimens (*see* also Muscioni^48^).

A quantitative survey of the stratigraphic distribution of fossil remains within the rhythmites indicate alternation of fossil-rich and fossil-barren beds, with larger elements confined into slumped intervals. However, most of specimens collected in the 1990’s resulted from massive quarry excavation and consequent mobilization of rocks.

Relevant to this study, a survey performed in early 2021 included the following individuals and associated elements referred to *T. insularis*:

1. SC 57021, type specimen (nicknamed ‘*Antonio*’^29^), currently on display at the MCSNTs. Both premaxillae, distal end of the right dentary, predentary have been fully prepared and removed from the main block;
2. SC 57022, partial, articulated forelimbs exposed at the MCSNTs^29^, nicknamed ‘*Primus’*, currently on display at the MCSNTs. The tail and potentially other skeletal elements are still *in situ*;
3. SC 57023, isolated left pubis^29^, currently on display at the MCSNTs;
4. SC 57024, partial scapula^42^;
5. SC 57025, cervical vertebra^30,42^, currently on display at the MCSNTs. Site relocated in 2020 showing large bones still *in situ.*
6. SC 57026, complete but heavily crushed skull currently on display at the MCSNTs, a large unprepared block containing the proximal vertebral column deposited at the MCSNTs, additional bones still *in situ* (nicknamed ‘*Secundus*’);
7. SC 57027, sacral vertebrae, MCSNTs;
8. SC 57028, a series of ten, fully prepared, articulated distal caudal vertebrae (*see* Muscioni, 2021);
9. SC 57247, articulated skeleton^46^ (this study) currently on display at the MCSNTs (nicknamed ‘*Bruno*’);
10. SC 57256, isolated rib, MCSNTs;
11. SC 57257, proximal end of a femur, MCSNTs
12. A series of thirteen, articulated, distal caudal vertebrae. Collected in September 2020, described in Muscioni^48^, no inventory number at the time of writing;
13. Large articulated elements still *in situ*, no inventory number at the time of writing (nicknamed ‘*Tertius*’);
14. Large articulated elements, still *in situ*, no inventory number at the time of writing (nicknamed ‘*Zdravko*’).

Based on inventory and field surveys, we therefore estimate seven articulated skeletons preserved at the VdP site and possibly up to 11 individuals of *T. insularis*.

**S.2.** **Extended anatomical description**

**Cranium**

*Tethyshadros insularis* was originally diagnosed on a mosaic of primitive and derived characters, the latter particularly present in the skull, such as a long antorbital region due to elongated maxillae and nasal bones^29^. Most of these features were based on the holotype, SC 57021 and described on the extraordinarily preserved dermatocranium (Fig. S5, S6) and postcranial material, except from some highly, taphonomically deformed areas in the sacro-caudal region, some details of the appendicular skeleton and the end of the tail, the latter missing in the type. SC 57247 (Fig. 2; Fig. S15, S16) represents a proportionally less complete skeleton, as it preserves the skull, pelvis, and entire tail, with some fragments and isolated material of the appendicular skeleton. Nonetheless, SC 57247 (Fig. 2a) includes a complete and articulated neurocranium, exposed on the left, lateral surface of the fossil due to the superficial erosion of some of the elements of the dermatocranium (e.g., the jugal and quadratojugal). Additional information can be retrieved from a third skull (SC 57026; Fig. S29) providing important new elements on the anatomy and systematics of this taxon. While a detailed description will be reported in a future monographic work which will deal with the description of the ten additional individuals, with at least an additional complete skeleton still *in situ* (‘*Zdravko*’, see inventory list above), we here reported the main anatomical features characterising the variation in this taxon, focusing in particular with those ontogenetically variable features and those anatomical units previously unknown, like the novel description here of the braincase.

The postorbital in SC 57021 (Fig. S5, S6) was originally described^29^ as inflated, thickened and with a rough surface. However, this morphological description does not entirely apply to the specimens after checking both complete and articulated postorbitals in SC 57021 (the right element) and in SC 57247 (the left one; Fig. 2b, Fig S5–S7). This tetraradiate bone has a long and slender ventral process, which is concave anteromedially and convex posteromedially. This ventral (jugal) process is anteroventrally inclined, contacting the dorsal process of the jugal with an anteroposteriorly thin contact. The lateral surface of postorbitals is flat in both specimens, but with an anteroposteriorly wider margin in SC 57247 than in SC 57021. The posterior process of the postorbital slides smoothly onto the anterior process of the squamosal, a contact more clearly seen in SC 57021 due to the partial preservation of the anterior process of the squamosal in SC 57247. In SC 57021 the postorbital contacts anteromedially the frontal and posteromedially the parietal (Fig. S5, S6). This anterior process is more dorsoventrally thick in SC 57247 (Fig. S5–S7). It is interesting to point out that the textural surface in the postorbital of SC 57247 is smoother and less pitted than the slightly more rugose dorsal-lateral portion of SC 57021, and some lateroventral rugosity persists mostly in the ventrolateral portion of the anterior process of the bone (Fig. S5). While the extent of thickening of the anterior process of the postorbital is by no means comparable of those seen in some derived hadrosaurids, like *Edmontosaurus*^49^ a similar trend is observable in the ontogenetic series of this taxon. It is noteworthy to mention though that while the inflated condition in saurolophine hadrosaurs, where the main thickening is focused on the dorsolateral rim on a central position, above the ventral process, it also affects, although to a lesser degree, the anterior process of the postorbital. The condition in *Tethyshadros* on the other hand, resembles most directly the morphology in *Levnesovia*^50^ and *Sirindhorna*^51^. The basioccipital is rounded caudo-ventrally with a sinuous ventral margin from the occipital condyle caudally to the basitubera anteriorly (Fig. S8). Basitubera are round and prominent, like in *Eolambia*^52^ and *Lesnesovia*^50^ rather than the more diminutive processes in derived hadrosaurids like *Acristavus*^53^, *Brachylophosaurus*^54^, *Edmontosaurus*^49^ and *Parasaurolophus*^55^. The paraoccipital processes of exoccipitals are eroded laterally, although only the general shape and dorsoventral extent in SC 57247 can be observed, while these elements are but completely preserved in SC 57021^29^ (Fig. S5). The rather robust exoccipital processes arch caudolaterally, reaching a ventral depth in their distal ends that terminates above the foramen magnum. This condition is closer to those in taxa like *Eotrachodon*^55,56^, and *Edmontosaurus*^49^ and dissimilar to the lower, more ventral extent reached by the exoccipital processes in taxa like *Jintasaurus*^57^, *Lesnesovia*^50^, *Eolambia*^52^ and *Parasaurolophus*^55^. The basipterygoid processes are dorsoventrally long (Fig. S8), anteroposteriorly short and slender until their slightly expanded and round articular ends: this relates to a medially deep basisphenoid recess (like in *Levnesovia* but differently from *Edmontosaurus*^49^ and *Probrachylophosaurus*^58^). Anterodorsally, the basipterygoid processes are continuous with an elongated cultriform process of the parabasisphenoid, reaching an anteriormost extent than the anterior surface of the presphenoid-orbitosphenoid. There are preserved foramina for the c.n. X–XII, set on the exoccipital-opisthotic complex in a sub-horizontal arrangement, slightly anterodorsally inclined, with c.n. X and XI at a relatively more anteroventral position than c.n. XII (Fig. S8). Two slit-like, ovoidal in shape and approximately equal in size exits for cranial nerves X and XI are presents, with shapes comparable to those in earlier diverging hadrosauriforms like *Levnesovia*^50^, while different from the more circular shape of hadrosaurids (like *Edmontosaurus regalis*^49^). A robust metotic strut separates the posterolateral exits of c.n. X and XI with the ones for the fenestra ovalis and metotic foramen. The metotic foramen, contains a confluent exit for both the c.n. IX and the internal jugular vein. A well-defined crista interfenestralis separates the fenestra ovalis anteriorly from the metotic foramen posteriorly (Fig. S8). There is a shallow anteroposteriorly directed ridge running parsagitattally through the dorsal half of the prootic and extending to the exoccipital process, similarly to *Levnesovia*^50^, *Lophorhothon*^55^ and differently from the deeper, thicker process in *Edmontosaurus*^49^. Anteriorly to the acoustic recess, a vertical ridge borders posteriorly the opening of the c.n. VII, but the broken up ventral margin of the prootic obscures the likely presence of a second, ventrally placed foramen for the internal carotid artery (Fig. S8). A posteroventrally directed ridge borders the ventral margin of a thickening of the prootic, which defines posteriorly a large opening for the trigeminal foramen (c.n. V; Fig. S8), which penetrates the rostroventral portion of that element, characterised by a shallow, lateral depression. The partial displacement of the prootic and the lateral coverage of the postorbital does not allow discerning whether the exit for c.n. V is composed by a single or split groove. The orbitosphenoid and presphenoid appear fully ossified, housing the exits for cranial nerves II–VI, apart from the already mentioned c.n. V (Fig. 2c, Fig. S8). Anteriorly to the laterosphenoid, in the caudal portion of the orbitosphenoid, an anterodorsally slit-like opening contains dorsally the exit for c.n. VI and ventrally of c.n. III. Anteriorly, a posteroventrally elliptical foramen contains the exit for c.n. IV, bordering the posterodorsal border of a ventrally directed V-shaped process, which is pierced anterodorsally by the circular exit of c.n. II. The hyoid in SC 57247 appears dorsoventrally thin and unexpanded, differently from the fan shaped distal ends present in SC 57201 (Fig. S5), but this is likely due to preservation, as the element is exposed in dorsoventral rather than lateral view (a condition exemplified by the hadrosauriform *Jinzhousaurus*^59^, where both hyoids are preserved and exhibit a different morphology analogous to both SC 57021 and 57247 combined because of their relative rotation along their parasagittal axis).

**Axial skeleton**

The poorly preserved sacral area in SC 57021 (Fig. S14) is affected by several lines of fractures and a wide fault line on its posterior side. Based on the identification of 5 distinct diapophyses, 5 sacral vertebrae are identified in continuity with the posterior dorsal centra, although partially obscured in this anterior area by the preacetabular process of the ilium. The fault line runs through past the 5^th^ sacral vertebra hampering a clear count of the exact number of elements in the sacrum. Posteriorly to the fault, two vertebrae are preserved with distinguishable neural spines. These are interpreted by Dalla Vecchia^29^ as sacrals with numbers 7^th^ and 8^th^, suggesting that the fault runs though enough space to justify the obliteration of a whole sacral vertebra, sacral 6^th^ accordingly. We interpret the block posterior to the fault as displaced from the remnant anterior part of the sacrum, making the two vertebrae on the posterior block sacrals 6^th^ and 7^th^ respectively (Fig. S14). Our interpretation is supported by what is described in the second specimen (SC 57247; Fig. S31). Four well preserved distalmost sacral vertebrae are present and exposed in lateral view in SC 57247 (Fig. 2a; Fig. S9, S15), of which the two distal centra only bear clearly defined and well-preserved neural spines. Anteriorly in the series, three aligned diapophyses, of which the two more caudal ones overlap the two most anterior centra of those four preserved ones mentioned above. The most anterior one in the series is connected to a badly preserved centrum, anterior to the main block of four contiguous sacral vertebrae. Anteriorly to the latter, two heavily damaged additional centra are present, bringing the total number of sacrals to 7. The size of these vertebrae and the relative occupied extent on the axial series does not leave much room for additional elements in the sacrum. The ilium is overturned from its articular position and lies on its lateral side, hiding the presence of at least sacral 4^th^ and 5^th^. The vertebrae 5^th^–7^th^ are the best preserved in the series (Fig. S9, S10), with an approximately box shaped morphology in lateral view, with equivalent dorsoventral and anteroposterior lengths. The centra of these vertebrae are fused between each other, showing two low keels running longitudinally in parallel on the ventral side, particularly developed on the 6^th^ vertebra and almost disappearing on the 7^th^. At least three well developed sacral ribs with wide flat articular surfaces for the ilium are present. The neural spines are only well preserved in sacrals 6^th^ and 7^th^, although only the dorsal portion are clearly exposed, while some fragmentary material obscures the base of their neural arches. The profile of both neural spines in 6^th^ and 7^th^ are rectangular and inclined caudally. The spine of the 6^th^ vertebra has a fracture running vertically at one third of its anteroposterior length, and both appear slightly offset from their relative centra.

A preliminary description of the caudal region of SC 57247 is reported in Dalla Vecchia^46^, although based on photos available online as the specimen was not yet fully prepared or accessible for study. The caudal series in SC 57347 (Fig. 2a; Fig. S11–13, S17, S18), is relatively well preserved and 43 (or 44: see below) caudals are present. The entire caudal series of SC 57427 is interested by a heavy diagenetic deformation, with clear traces of torsion and extensional deformation, with some fractures running through multiple planes (Fig. S10, S15, S16). The caudal vertebrae are not lying on a single plane, exerting differential tractive forces on the caudal series. For this reason, the more proximal caudal vertebrae, closer to the sacrum acted as a different unit during post-mortem disarticulation compared to the more distal portions of the tail. The centrum of the sacral 7^th^ is partially obscured by the neural spine of the vertebra caudal 1^st^, followed by two badly preserved centra. The first caudal neural spine is associated to a badly preserved centrum for the 1^st^ caudal vertebra. Caudal vertebra 1^st^ (centrum and neural spine) lies posteriorly to the sacral 7^th^, and is affected by vertical breakage, artificially increasing the distance between the anterior and posterior surface of the centrum. The transverse process of caudal 1^st^ is wide and rectangular in overall shaped, but is plastically twisted dorsally on the axial plane, lying onto the lateral side of the neural spine. Caudal vertebrae 1^st^–9^th^ are articulated and exposed in lateral view. The centra are anteroposterorly longer than dorsoventrally high, apart from the centra 3^rd^ and 4^th^. This is likely due to some damaging of the dorsal surface of the centra and anteroposterior compression in that area due to tectonic deformation. Neural arches of the first two caudals are partially overlapped and hidden by some bony fragments and matrix that could not be removed during preparation. Transverse processes in caudal 2^nd^ – 4^th^ have been restored during preparation, and their relative posterior position on the centrum, sometimes leaning towards the interarticular space between centra (particularly between caudals 1­^st^ – 2^nd^ and 2^nd^ –3^rd^ is likely an artifact of preparation. Although transverse processes in caudals 5^th^ –8^th^ have been glued back to the centra as well, their relative morphology, progressively less elongated and anteroposteriorly less expanded at their distal ends show less artificial deformation than in the 1^st^ ­–3^rd^ interval. In caudal 9^th^–11^th^ the transverse processes are incomplete with only their base close to the centra being preserved as a low ridge dorsolaterally oriented and missing their distalmost ends. Caudal 11^th^ is the last one showing evidence of a transverse process, due to the progressive rotation of caudal vertebrae exposing only their ventral surface in most of the rest of the series. For the same reason, zygapopheses are visible only up to the 9^th^ vertebra in the caudal series (Fig. S12), with the only exception of vertebra 14^th^ of which the left prezygapophysis is visible due to the partial disarticulation of the neural arch to its relative centrum. The remaining prezygapophyses are well preserved and articulated in vertebrae 3^rd^ – 9^th^. The asymmetrical compression on both sides of the skeleton causes a partial deformation on both sides of centra 3^rd^–5^th^ and 8^th^–9^th^, hiding the articular surfaces of the centra. The prezigapophyses in many vertebral elements are mostly hidden by the postzygapohyses they articulate with. Neural spines are well preserved up to the caudal vertebra 10^th^ but less preserved at the base of their processes. The neural spines of caudals 3^rd^–7^th^ are partially deformed and slipped in their top half caudally, exhibiting an unnatural S-shape (Fig. S11). Posteriorly to caudal vertebra 8^th^, the neural spines become progressively less anteroposteriorly expanded, being also dorsally less squared-off compared to the first 8 caudals in the series and more rounded in anteroposterior views. Starting from caudal 10^th^ up to 20^th^, vertebrae are progressively bent around their own anteroposterior axis exposing only their lateroventral side, with caudal 20^th^ further rotated of 90° on its sagittal plane, placing its ventral surface (Fig. S12, S17) posteriorly to the posterior articular facet of caudal vertebra 19^th^, increasingly hiding their neural spines. Vertebral centra 9^th^ and 10^th^ are badly preserved, 19^th^ is incomplete on its cranial extremity and a series of fractures run through centra 13^th^, 14^th^, 17^th^, 18^th^ and 20^th^ splitting these centra in two sagitally subequal halves. These ventrally exposed vertebrae, together with the centra of caudals 21^st^ and 22^nd^ expose the haemal sulcus (or canal^60^; Fig. S12, S17). The last visible and preserved neural spine along the caudal series is that of the 24^th^ vertebra. These processes are present from the 1^st^ to the 36^th^ caudal vertebra in *Ouranosaurus*, and completely disappear beyond the 27^th^ caudal in *Iguanodon bernissartensis*^61^, making this last observation most likely preservationally biased. Caudal 23^th^ and 24^th^ are exposed in lateral views, while the series 25^th^ –37^th^ are rotated exposing their ventral views, and 38^th^–41^st^ are exposed again in lateral view. The distalmost elements preserved, caudal 42^nd^ and 43^rd^ are exposed in lateroventral views. The vertebrae 29^th^ –38^th^ when exposed in lateral view exhibit reduced and slender zygapopheses, mostly overlapped by their contiguous centra. In the same interval, the most proximal vertebrae exposed in ventral view present an hemal sulcus (Fig. S13, S18). This ventral furrow (Fig. S13, S17, S15) is also present in SC 7247 between centra 13^th^ and 22^nd^, a feature hidden in SC 57201 by the matrix. This character is common in ornithopods and has been reported in *Telmatosaurus transsylvanicus*^62^. Nopsca^63^ suggests the presence of a haemal arch in the caudal vertebrae of *Zalmoxes* and *Telmatosaurus transsylvanicus* to be a sexually dimorphic character (present only in male individuals), but Verdú et al.^64^ showed how this character is subject of individual variability in specimens of *Iguanodon bernissartensis*. Clearly visible articular facets for chevrons on the ventral surfaces of vertebrae are visible from caudal 7^th^ to 24^th^, with particularly wider surfaces in the interval 8^th^–12^th^ (Fig. S17). Vertebrae 22^nd^ –28^th^ are incomplete due to cutting of the slab for extraction. There are two main folds along the caudal segment, a 180° one around caudal 8^th^–11^th^ and a less extreme one folded towards the opposite direction along caudals 24^th^–27^th^. These two folds impose a Z-shaped overall morphology to the caudal series. There are wider spaces between the vertebrae 21^st^ –24^th^, 36^th^ – 37^th^ and 41^st^ –42^nd^ which may apparently suggest the original presence of additional centra in between them. On the other hand, given their measurements and relative proportions, these spaces are more likely the effect of sliding and slight disarticulation rather than lacunae from secondarily removed original elements. In the entire caudal series, 17^th^ chevrons are preserved, with a fragmentary one preserved ventrally to centrum 26^th^. Although slightly caudally displaced, haemal arch 7^th^ is the first preserved one, with a contiguous series complete up to caudal 19^th^. Caudal chevron 22^nd^ is preserved articulated with its relative centrum, while a floating one is disarticulated in between this and the most distally preserved one (Fig S12), associated with centra 25^th^ and 26^th^. Their overall shape is elongated and progressively shorter as the series progresses posteriorly, becoming shorter and fin shaped in the caudals 19^th^–26^th^. A pattern consistent with what observed in SC 57021.

A great deal of speculation has been made on the morphology and overall measurement of the tail in *Tethyshadros*^46^. A comparison between the two caudal series of SC 57201 and SC 57247 is probably the best approach to identify the relative position of the caudal elements and the most likely length of the tail in *Tethyshadros* (Fig. S19). Due to the lack of some elements, the relative rotation, disarticulation, or displacement of some of the centra, it was not possible to take measurements of some vertebral elements. On the other hand, the overlap between the tails of both individuals allowed a morphometric comparison (Data S1; Fig. S19), which helped identifying the relative position in the series of each vertebral centrum and inferring more accurately the purported end of the tail in the missing distal portion of the holotype. This information can be used to infer peculiar morphofunctional adaptation related to the tail^65–69^. Dalla Vecchia^46^ used his morphometric reconstruction of the tail length (inferring the presence of 77 elements^46^) to attribute a peculiar function, possibly related to aberrant insular adaptations. We here tested these morphometric observations, outlining the underlying data quality of the sample. In SC 57021 caudals 5^th^ and 17^th^ are damaged, while part of the distal ends in caudals 31^st^ and 33^rd^ were artificially restored during preparation. In addition, caudal 11^th^ and 13^th^ are anteroposteriorly compressed, so an error margin in measurements must be taken into account. Caudals 32^nd^ and 33^rd^ were excluded from our dataset (Data S1) because of the heavy amount of their restoration. The slight displacement, preservation and positions make the measurements of vertebrae 1^st^–3^rd^ and 7^th^ – 9^th^ too uncertain to be included in the dataset. The incompleteness of the tail in the holotype (SC 57021) does not allow to analyse the morphometric trends beyond the 33^rd^ caudal. On the other end, the articulation of a contiguous series of 33^rd^ vertebrae in SC 57021 allow a more reliable interpretation of these measurements in the most proximal portion of the tail. The relative fluctuation of any measurements in the tail might be increased by deformational processes affecting the vertebrae. This is particularly evident in caudal 1^st^, 9^th^ and 10^th^, which for these reasons have been not included in the dataset. On the other hand, the higher completeness of the vertebral series in SC 57247 allows to investigate and describe the morphology of the distal portion of the tail, missing in the holotype. The vertebral profile (measure of anteroposterior length of the centra; Fig. S19; Data S1) in SC 57247 is very similar to that of the holotype (SC 57021), with a series of peaks and throughs in the length of the centra matching closely the trend in the holotype but with some relative proportional differences due to the different absolute size (Fig. S19f) of the two individuals (see relevant section on osteohistological analyses). The morphology of the most distal median centra in SC 57021 is peculiar in starting to become more elongated and cylindrical in shape between caudal 23^rd^–33^rd^, progressively more dorsoventrally compressed, appearing mediolaterally wider than dorsoventrally high, and with a flattened dorsal surface between vertebrae 28^th^ – 33^rd^. These same proportions could not be observed in SC 57247 since the vertebrae along this series are preserved on a different plane and hard matrix surrounds the lateral surfaces of the centra. The proximal portion of the tail is likely the less comparable due to the bad preservation of this portion in the holotype and ad the heavy deformation in SC 57247. On the other hand, 10^th^–12^th^ and 15^th^ to 20^th^ follow the same trend, with an anomalous peak at the vertebra 10^th^ in the holotype due to the fragmentation of this element. The more extreme oscillation in the proximal vertebrae of SC 57247 is due to the bad preservation in this area, where different level of plastic compression and longitudinal deformation has hidden the real anatomical trend in this anatomical region. The parallel trend in both series in some portion of the caudal series show though that some of these trends are a real palaeobiological signal and not just an artifact of preservation. Regarding the neural spines, a few interesting variations in proportion happen throughout the series. In SC 57021 there is a shortening in the antero-posterior lengths of the neural spines happening between 6^th^–10^th^ and in 6^th^–7^th^ in SC 57247. A significant decreasing in dorsoventral length of the neural spines occurs in SC 25021 in 14^th^ –16^th^. In SC 57247 the same transition happens between 10^th^–12^th^, although this might be partially biased by the slight rotation on the longitudinal axis of the vertebrae in this interval, shortening the dorsoventral length of the spines in this proximal portion. In addition, these vertebrae appear slightly more caudally inclined in SC 57247, but this is clearly a preservational artifact due to a slight posteriorward slipping of the vertebrae in this area (clearly noticeable in caudals 4^th^–6^th^. Given the several diagenetic artifacts affecting both skeletons of *Tethyshadros*, the length of the centra is the most conservative and reliable proxy to infer the morphometrics of the tail. Dalla Vecchia^46^ interprets the extreme proximity of contiguous articular surfaces in SC 57247 as evidence of short to non-existent intervertebral disks, hence a relative lack of flexibility in this taxon’s tail with an extreme rigidity of this proximal portion of the tail. On the other hand, the relative lack of space between centra is most likely due to taphonomic processes, in particular the anterior-ward sliding of the tail and the post mortem ventrally arching of the tail beyond the *in vivo* potential mobility range. This taphonomic displacement is evidenced by the distancing of zygapophyseal facets in between contiguous vertebrae in the series. While the relative dorsal orientation of the zygapophyses in the proximal portion of the tail and the relative proportion of both centra and neural arches surely represents a relative rigidity to mediolateral motion in this taxon, this is probably nor due to a purported reduction of intervertebral spaces, nor beyond the likely range in other closely related dinosaurian taxa^70,71^.

Throughout the series, minor signs of restoration are also present in vertebrae 15^th^ and 18^th^. The amount of restoration of haemal arches in SC 57247 following the morphology in the holotype as a reference makes the morphology of the chevron a poorly reliable feature to make any inference on the tail length and the relative position of the centra associated to these chevrons. Dalla Vecchia^46^ reported that the haemal arches in the proximal caudals of the holotype are tongue shaped, while those in SC 57247 are spatulate, meaning that they are more expanded anteroposteriorly at their distal ends that at their proximal articulation with the centra. This has been proposed^46^ to bear some morphofunctional implications discussed by the author. As we mentioned above, given the poor preservation of these elements, which are largely restored at their distal ends, assessing any further morphological remark and functional implications is quite speculative in this taxon. Because of the lack of some preserved neural spines (beyond the 29^th^ centrum) in SC 57021, the most reliable elements to perform any comparison are the centra.

The total number of caudal vertebrae in *Tethyshadros* has been uncertain so far^46^ since the series is not complete in the holotype described by Dalla Vecchia^29^. SC 57247 includes 43 elements, and although some lacunae might be present in places of major preservational displacement throughout the series (like between caudals 23^rd^-24^th^, 37^th^-38^th^, and 41^st^-42^nd^) and possibly beyond the distalmost elements preserved. These potential lacunae between elements affect the interpretation of the tail length. The number of caudal vertebrae in hadrosauriform dinosaurs ranges from 50 in the most basal members of the clade^72^ to more than 70 in more derived members of the clade^46,70,73,74^. In Dalla Vecchia^46^ a different estimate on the tail vertebral count (77) is reported based on the assumption that the high number of vertebrae in the derived indeterminate hadrosaurid TMP 1998.058.0001 from Dinosaur Provincial Park would be highly reflective of the condition in *Tethyshadros insularis*. Considering several combinations of lacunae throughout the spaces left between block of vertebrae in articulated series in SC 57247, we estimate that no more than 6 vertebrae may be missing. We reconstructed the tail morphology (and its length) according to different patterns of gap filling (Fig. S20). We followed different alternative reconstructions where we added 6 caudal elements after the distalmost one preserved in the series (scenario A) and ignoring the lacunae throughout the series. In scenario B, one vertebra is added after vertebrae 23^rd^, 38^th^ and two vertebrae past the 38^th^ and 40^th^ vertebra, and one after the 42^nd^ caudal. In scenario C, 3 elements are placed after the 23^rd^ centrum, and other 3 after the 40^th^ vertebra. Finally, in scenario D, 5 vertebrae are added after the centrum 23^rd^ and one after the 42^nd^. Given the measurements and relative proportion of vertebrae 23^rd^ and 24^th^ (Fig. S19) we consider an additional vertebra between them unlikely. Although the exposition in different views of these elements, given the somewhat larger morphological dissimilarity between vertebrae 37^th^ and 38^th^, we consider the addition of elements in between these centra, as in scenarios B and D, the most likely. The distance between vertebrae 41^st^ and 42^nd^ is most likely related to a slight displacement of the more distal element in the caudal block. Although we cannot be 100% certain that the posteriormost vertebra preserved is the distalmost in the caudal series, since its external morphology is damaged and partially covered by matrix, its relative proportions are compatible with those of a terminal caudal vertebra. Here, following scenario B, we consider a total vertebral count for the tail in SC 57247, and then in *Tethyshadros insularis*, of no more of 49-50 vertebrae (Fig. S20).

In conclusion, while we cannot be confidently sure on the total length of the tail nor on the total number of caudal vertebrae, we can certainly rule out a vertebral count exceeding the 50 caudal vertebrae (and likely ranging around 44–51 caudals; Fig. S20) or even remotely approaching the 77 elemental count suggested by Dalla Vecchia^46^. Based on the absolute size and relative proportions, SC 57247 appears to be larger than the holotype although it is difficult to estimate exactly how larger than SC 57021 it is. Considering the linear dimensions of the skull and overlapping tail elements, taking into account the potential serial discrepancies between the relative position of the centra in the two individuals, an estimate of 15–20% of larger size in SC 57247 can be inferred compared to SC 57021.

**S.3.** **Body mass estimation for *Tethyshadros insularis***

Body-size for *Tethyshadros insularis* was originally performed by Benson et al.^75^ using equations on humeral and femoral measurements provided by Campione and Evans^76,76^ to estimate dinosaur body masses. This method has been shown to converge on the most accurate estimates for body-size estimates from multiple methods^77^. While the humerus is missing the older specimen, hampering a direct application of the body-mass equation from Campione and Evans^76^ and Campione et al.^78^, the relative proportions between linear measurements in SC 57021 and SC 57247 allowed to infer, with a certain margin of uncertainty a 15–20% larger size in the bigger, newly described specimen compared to the holotype. These values take also in account the different relative age of the two individuals based on the osteohistology of their ribs (Fig. S21-22). Given a body size reported in Benson et al.^75^ for the holotype of 338.1776 kg, we applied the formula for isometric scaling where the length of SC 57247 was divided for the length of the holotype elevated at its third power and multiplied for the body-mass of the holotype, accounting for a 5% uncertainty between the likely difference in estimated length between specimens we considered here. Applying this method, an estimate of 514.33–584.37 kg was obtained for SC 57247. We conservatively used the median value between these confidence intervals (548.60 kg) for the body-size evolutionary modelling implemented in this study. We would like to point out that given the uncertainty in the nature of the External Fundamental System in the larger, more mature individual (see “Osteohistology” section in “Materials and Methods” and “Ontogenetic stages of Tethyshadros specimens in the “Results”), the possibility this individual was still actively growing, although likely at a slower rate to justify large increase in body-size, is still potentially possible.

**S.4.** **Phylogenetic character scoring**

Notes on characters scoring are herein reported based on the revaluations of the specimens examined in this study, revising older scorings reported by several authors (e.g. McDonald^59,^ Takasaki et al. ^94^), and mostly based on the description reported by Dalla Vecchia^29^. Observations were based on SC 57021 (holotype, nicknamed “Antonio”), SC 57257 (second most complete skeleton, older individual nicknamed as “Bruno”), SC 57022 (isolated but articulated antebrachia, including autopodia) preliminary described in Dalla Vecchia^29^ and nicknamed “Primus” and SC 57026, an isolated and disarticulated skull here nicknamed “Secundus” (Fig. S29). Characters were previously reported as based solely (or mostly) from examination of the holotype, SC 57021. Many dentition-related characters were here scored from SC 57026 (Fig. S30), since the features of the fine details of enamel and tooth morphology is better preserved in this specimen. Here we reported only those revised scores, restating confirmation of character states where we agree with previous authors only for more controversial characters, whereas explicit information in previous literature was scant or absent. Phylogenetic scorings are here highlighted with character number in the list of Takasaki et al.^79^ and the selected character state separated respectively by a colon. Unknown characters are here discussed and scored using question marks “?”.

The shape of septa that separate the dentary alveoli was rescored as thin and sheet-like (13:1) rather than as with thick and stick-like septa (13:0). The number of teeth per cm in the dental battery is here confirmed to be less than 1.25 per cm (17:1), like previously stated in Takasaki et al. (2020). The presence of marginal denticles in teeth (character 23:0) could be confirmed in SC 57026 (Fig. S29-S30). The spacing of marginal denticles in the predentary, is scored differently in Antonio and Bruno, while the status is derived in Bruno (spacing shorter than width of each denticle) it is 28:0 in SC 57021. Since the character is defined for adult, the status represented by SC 57247 is used in the *Tethyshadros* OTU. The spacing between two adjacent denticles of the predentary, is here being rescored as 28:1 as observed in SC 57247 (differently than 28:0 as observed in the holotype), with a spacing shorter than the width of each denticle. Since this is an ontogenetically dependant character (formulated in the adult status), we kept the condition in SC 57247 as a more reliable proxy of the adult condition. Development of the dorsal median process of the predentary is rescored as 35:0, with the presence of a faint dorsal median process that is slightly protruding posterodorsally, rather than the previous scoring as 35: 1 (presence of a well-developed dorsal median process without a prominent ridge). This area is damaged in the holotype but can be scored in SC 57247. Characters 38 and 43, both dealing with ratios between measures of the dentary were previously either scored or left unscored, but these characters cannot generally be assessed in any specimen since the anterior portion is damaged with anteriormost teeth not preserved in SC 57247, while they are distorted in SC 57026 and are hidden by other elements details in the holotype. For the same reason, the angle between the medial surface of dentary symphysis and the lateral surface of the anterior region of the dentary in ventral view (character 44) which was not scored by Takasaki et al.^79^ could be observed in SC 57247 and SC 57026. Charater 49, related to the distal expansion of the coronoid, was previously scored in Takasaki et al. (2020) as only slightly expanded anteriorly, with less developed anterior and posterior margins, but in SC 57247 it is clear how this process gains a more robust morphology, with a markedly expanded anteroposteriorly long apex (Character 49:1). Characters 50, 52–53 related to several views (mostly posterior and medial) of the coronoid process of the dentary could not be accessed in the specimens and were left unscored. A sharp projection on the posterodorsal surface of the coronoid process of the dentary is scored as absent after observations in SC 57021, SC 57026 and SC 57247. The presence of a relatively inclined longitudinal axis of the dentary in occlusal view (54:0), forming an angle of about 20º with the lateral margin of the dentary could be scored after observations in all the specimens preserving a dentary, particularly in the specimen SC 57026 which has a disarticulated dentary that can be minorly manipulated more than the other specimens. A lingual curvature of the longitudinal axis of the dentary occlusal surface in dorsal view was previously scored as absent (55: 1) based on the holotype but it is present (55:0) in SC 57026. The presence of a surangular foramen, was scored following Dalla Vecchia^29^ initial description as absent (59:1). Although it might still be present since some matrix still covers and some distortion affect this area, we rely now on our multiple observations to keep the original scoring referring to this feature as absent in the coronoid process. Character 60, the presence of a surangular accessory foramen on the anterolateral surface of the surangular is scored as absent (60:1). While character 71 (shape of the premaxillary oral margin in dorsal view) was previously scored as broadly arcuate in oral margin, we could not assess this feature in any of the specimens because of damage. A premaxillary foramen in the anterior region of the external naris that opens onto the palate is scored as absent (76:0) after observation in SC 57247. An oval foramen on the anteromedial surface of the premaxillary lip, could not be assessed in any specimen due to preservation, so the original scoring was changed into unscored (80:?). We could confirm in SC 57247 the development of the premaxillary posterodorsal process in adults, as originally scored (based on the holotype) for being relatively short, forming the dorsal margin of the external naris with the nasal (83:0). A relatively short premaxillary posteroventral process (84:0), anteroventral to the prefrontal in adults was confirmed in SC 57247. Character 86, a vertical groove on the posteroventral process of the premaxilla, located anterior to the dorsal process of the maxilla (86:0) is confirmed as absent after checking on SC 57247 and 57026. A vertical groove on the posteroventral process of the premaxilla, located anterior to the dorsal process of the maxilla which was scored as absent in the holotype could not be confirmed in our observation of the same specimen, but appears to be the case in SC 57247. The location of the apex of the adult maxilla in lateral view is markedly posterior to the midline of the maxilla (101:0) rather than at or anterior to the midline of the maxilla (101:1), based on SC 57247. The shape of the dorsal ramus of the maxilla in lateral view is triangular, with the width greater than the height (102:0). The presence of a low, dorsolaterally facing subquadrangular jugal contact surface located anterodorsal to the ectopterygoid shelf (103:1) could be confirmed in SC 57021 but cannot be scored in both SC 57247 and SC 57026. Character 105, The number of the maxillary foramina anteroventral to the jugal contact facet was scored as more than six, but although some foramina seem present in SC 57247 and they were originally scored as “at most six” in the holotype (105:1), we could not accurately confirm this on specimens, so this feature was left unscored. The ratio between the vertical distance from the apex of the maxillary dorsal process to the maxillary ventral margin and the maximum length of the maxillary ventral margin was quantified as up to 0.35 (110:0). Characters 11­1–114 are palatal characters which unfortunately cannot be observed in specimens because of lack of preservation of this anatomical area, so were left unscored. Character 130, the position of the quadratojugal notch relative to the dorsoventral height of the quadrate, was scored by Takasaki et al.^79^, but the presence of such notch in the first place could not be observed in any of the specimens. Similar observations could not be tested because of the lack of preservation in these elements for characters 131 and 132, relative to the suspensorium (quadrate-quadratojugal) area. A squamosal buttress (posterodorsal protuberance) on the posterior side of the dorsal end of the quadrate was scored as absent or less developed (133:1) like in Takasaki et al.^79^. A medial condyle that is dorsally and markedly elevated in relation to the lateral condyle (135:1) is scored by Takasaki et al.^79^ but due to preservation, the morphology and relative position of the medial condyle cannot be evaluated in any specimens, so is updated as unknown (?). This is the same for other quadratojugal related features, like those encoded in character 136. The lateral profile of the lacrimal in articulated adult cranium was scored as trapeziform and anteroposteriorly elongate, bearing a dorsoventrally low, relatively long anterior process based on the holotype (170:1), but a bulkier, more dorsoventrally deep morphology which is subquadrangular or trapeziform, with a relatively short anteroventral corner (170:0) is clear in SC 57247 (although with a less deep morphology in the holotype). A dorsal promontorium on the dorsal surface of the postorbital anterior process in adults was scored as absent (178:0) in the holotype^79^, but such feature (178:1) clearly develops in older individual, as observed in SC 57247 (Fig S5–S7). A proper frontal doming (character 196) is not present (196:0) both in the holotype and in SC 57247. The length/height ratio of the skull is scored as 230:0 in SC 57247 (1.49) while it was considered SC 57021 in the holotype, expressing the variation in anteroposterior elongation between differently aged specimens. The ratio between the anteroposterior length of the interfrontal suture of the frontal ectocranial surface and the maximum mediolateral width of the frontal ectocranial surface in dorsal view could not be scored since the two specimens preserving this region are mediolaterally compressed, so the original scoring was turned into an unscored character (198:?). The lateral profile of the parietal sagittal crest was originally scored as (203:0), with a sagittal crest being nearly straight or slightly down-warped, level with the skull roof; but a derived condition, with a nearly straight (203:1) crest, gradually ascending posteriorly relative to the skull roof is the condition exhibited by SC 57247. Character 206, which quantifies the angle between the paired posterolateral secondary ridges of the parietal sagittal crest was originally scored but it cannot actually be assessed because of mediolateral compression of the specimen. Characters 209-210, related to the degree of expansion of the exoccipital processes cannot be assessed because of preservation, so the original scorings were turned into unknown (?) for these characters. The morphology of the basypterigoid processes of the basisphenoid as short (214:0) or long (214:1) have been scored as 214:1 based on SC 57247 (Fig S8). Character 215, the angle between the basypterygoid processes cannot be assessed since the braincase is mediolaterally compressed. The development of the paired alar processes of the basisphenoid was scored as moderately developed and relatively small (246:0) based on the newly described braincase in SC 57247. A complete closure of the forward sulcus as the passage of the ramus ophthalmicus of the trigeminal nerve (V1) on the lateral surface of the laterosphenoid is scored as absent (0) as observed in SC 57247. Character 221, a great reduction of the length of the postorbital process of the laterosphenoid, is here scored as being absent in SC 57247 and used as morphology for the taxon. The general shape of the orbit in lateral view is scored as almost circular (0). Character 229 (general shape of the supratemporal fenestra in dorsal view), cannot be properly assessed because of the mediolateral compression of all the specimens, the character is hence rescored as 229:? contra the original scoring in Takasaki et al.^79^. The length/height ratio of the skull is 2.47 (higher than 2, so condition 230:1) in the holotype while it is 1.51, so scored as 230:0 in SC 57247, so likely an ontogenetically variable character, deepening and shortening the skull in adult individuals. Shape of the occiput in posterior view is scored as trapezoidal but cannot be properly assessed because of the mediolateral flattening of all the specimens, the character is hence rescored as 233:? contra the original scoring in Takasaki et al.^79^. The basisphenoid participates (235:0) in the anteroventral margin of the foramen for the trigeminal nerve. The number of the sacral vertebrae was originally scored^79^ as 8 or more (244:1) but after the description herein reported of the better-preserved sacrum in the older individual (SC 57247), it is rescored as 244:0, with sacral vertebrae that are clearly no more than 7. For the same reason, a participation of the caudal vertebrae to a synsacrum is being checked and proven inconsistent with the anatomy of both the holotype and SC 57247, and so rescored from what reported in Takasaki et al.^79^ (246:1) to absent (246:0). While *Tethyshadros* was originally scored exhibiting caudal vertebrae participating to the sacrum (246: 1), this has been rescored as potentially including two states (0 and 1) since we cannot be 100% sure of the participation of the cranialmost caudal vertebrae to the sacral district (see description and comparison between the holotype and SC 57247). Character 252, the ratio between the length of the lateral margin of the scapular articular surface and that of the lateral margin of the glenoid was previously scored, but it cannot be properly assessed since the proximolateral surface of the scapula in the holotype is eroded, while most of the coracoid is covered by other elements (the same elements are poorly preserved in SC 57247). Similarly, the angle between the lateral margin of the scapular articular surface and the lateral margin of the glenoid of the coracoid (character 253), while characters 254 and 255, scoring the morphology and proportion of the hook-shaped ventral process of the coracoid, cannot be assessed since the coracoid is covered by the humerus in the holotype and only minorly preserved in SC 57247. Character 257 relates to the ventral morphology of the coracoid: compared to how this feature is represented in the reconstruction present in Dalla Vecchia^29^, the ventral margin of the scapula is way straighter and less curved than herein reported. The coding confirms the rescoring already adopted by Takasaki et al.^29^. Character 261 expresses the ratio between the dorsoventral depth of the scapular neck and the maximum dorsoventral depth of the scapular proximal end, which was scored as 261:0, meaning that it is up to 0.6, which is confirmed as the condition in holotype, since this measurement is 0.58 in the SC 57021. A presence of a poorly developed deltoid ridge (253:0) on the humerus which is dorsoventrally narrow and relatively faint has been scored for this taxon. Character 265 was scored by Takasaki et al. (2020), but not only posterior view of the humerus is half inaccessible (most of the medial side is encased in sediment from the matrix) but there is also strong mediolateral compression on the specimen, so the character was rescored as 265:?. Character 266 related to the development of the deltopectoral crest in adults, but since SC 57247 lacks a humerus, this character cannot be scored. A strong constriction of the distal half of the humerus below the deltopectoral crest (corresponding with Xing et al., 2012^80^: character 256) is present in the holotype (268:0). Character 269, 270 and 272 were scored in Takasaki et al.^79^ but relate to proportion with mediolateral measures of the humerus, which given the mediolateral compression of the holotype, or the lack altogether of the element in SC 57247, these characters cannot be assessed. Character 289-290 and 292 quantify ratios between measurements of the ilium, but this area is heavily deformed, so they are all scored as unknown (?). A lateroventral expansion of the iliac supraacetabular process was scored by Takasaki et al.^79^ as extremely expanded lateroventrally and nearly overlapping the whole central plate of the ilium in lateral view (293:3), but we rescored this character as being moderately expanded lateroventrally, with its lateroventral margin located at approximately half dorsoventral height of the iliac central plate (293:2). Character 299 relates to the ischial peduncle of the ilium, which is covered by the ischium in the holotype, and so cannot be scored (299:?). The general profile of the postacetabular process of the ilium in lateral view was scored as rectangular, with a straight or arched posterior margin (301:1) contra the original scoring by Takasaki et al.^79^, which was scored as gradually tapering posteriorly, forming a wedge-shaped postacetabular process (301:0). The brevis shelf at the base of the postacetabular process of the ilium was previously scored (Takasaki et al.^79^) as absent (302:1) and although preservation does not allow to check this feature in SC 57247, this is likely the case. Character 303 describes a mediolateral thickening of the posterior portion of the iliac postacetabular process: this character was originally scored as absent, with the posterior portion of the postacetabular process being mediolaterally compressed (303:2), but given the deformation in both SC 57021 and SC 57247 we cannot assess this character (scored as 303:?). The dorsoventral depth of the anterior blade of the pubic prepubic process relative to that of the pubic acetabular margin) as was scored as 310:0 (the dorsoventral depth of the anterior blade is greater than that of the pubic acetabular margin), rather than 303:1 (the dorsoventral depth of the anterior blade is equal to or less than that of the pubic acetabular margin), as in Takasaki et al.^79^. The anteroposterior length of the anterior blade of the prepubic process relative to that of the posterior neck (posterior constriction) of the prepubic process was scored as being less than that of the posterior neck (311:0), but this character cannot be properly assessed because of poor preservation, and so was rescored as unknown (311:?). A pubic obturator notch ventral to the ischial peduncle of the pubis for the passage of the obturator nerve was scored as absent or bearing a faint ridge character (314:1) by Takasaki et al.^79^ but the pubis is covered by other elements (mainly the femur) in this area, which is also badly preserved, so it cannot be scored (314:?). Length/width ratio of the ischial peduncle of the pubis (character 315) could not be assessed because of mediolateral compression of the specimen, so this character was rescored as unknown (315:?). Character 317 represents the ratio between the anteroposterior distance from the anterior margin of the prepubic process to the acetabular margin and the dorsoventral distance from the dorsal margin of the iliac peduncle to the ventral margin of the anterior end of the postpubic process. While originally scored by Takasaki et al.^79^ we think this measurement cannot be assessed (317:?) in the specimen due to preservational issues. A posterior curvature of the iliac peduncle of the ischium (corresponding to Prieto-Márquez and Wagner, 2009^81^ [2] character 275): is scored as present, with the ischial iliac peduncle is slightly curved posteriorly (318:0) rather than markedly curved (318:1) or absent (318:2). Characters 320 and 321 refer to measurements of the iliac peduncle of the ischium, hence these have been left unscored as this element is not accessible in the specimen. An anteroventrally oriented axis of the ischial pubic peduncle relative to the ischial shaft (322:0) was scored rather than anteriorly directed (322:1). The absence of a slightly curved posteromedially distal half of the femoral shaft was scored by Takasaki et al.^79^ but since most of these portions of the preserved femora in all specimens are either covered or restored because of their poor preservation (331:?). The lesser trochanter on the anterolateral surface of the proximal portion of the femur was scored as strongly developed lesser trochanter (332:0), rather than moderately developed, and possibly fused to the greater trochanter as in Takasaki et al.^79^. The cnemial crest of the tibia (character 335) was scored as expanded ventrally along the proximal half of the tibial shaft (335:1), as observed in the holotype and previously scored by Takasaki et al.^79^. The general shape of the distal end of the fibula in lateral view as seen in SC 57247 is best described as subtriangular, forming a moderately expanded anteriorly fibular distal end (336:0). We cannot really discern whether distal tarsals II and III are present or not (character 339:?) because of preservational conditions in the distal hindlimbs. Character 341 is the ratio between the proximodistal length of the metatarsal III and the mediolateral width of this element at the midshaft, which is lower than 4.5 (341:1) rather than higher (340:0) as reported by Takasaki et al. (2020). Character 343 is the ratio between the mediolateral width at the midshaft and the proximodistal length of pedal phalanges III-2 and III-3, which is scored by Takasaki et al.^79^ as being greater than 3, but that we measured as lower than that (343:0). The ratio between the mediolateral width at the midshaft and the proximodistal length of pedal phalanges IV-2, IV-3 and IV-4 is lower than 3 (344:0). Character 346 refers to the plantar ridge on the pedal unguals but this feature cannot be observed in the specimens available as all ungual phalanges are preserved exposing their dorsal surfaces, hiding their ventral views, so this character was rescored as 346:? contrary to Takasaki et al.^79^, where was scored as 346:0. The posteroventral margin of the quadratojugal was originally not scored for *Tethyshadros* by Takasaki et al.^79^*,* but we could describe it as gently angled or have a small projection (351: 0), rather than forming a large hook-like process (351:1) in the holotype. Similarly, the prenarial region anterior to the circumnarial ridge can be described as short (353:0) rather than elongated (353:1) based on SC 57247. Finally, a postorbital process of jugal is smooth in the holotype (354:0) rather than preserving a horizontal shelf (354:1).


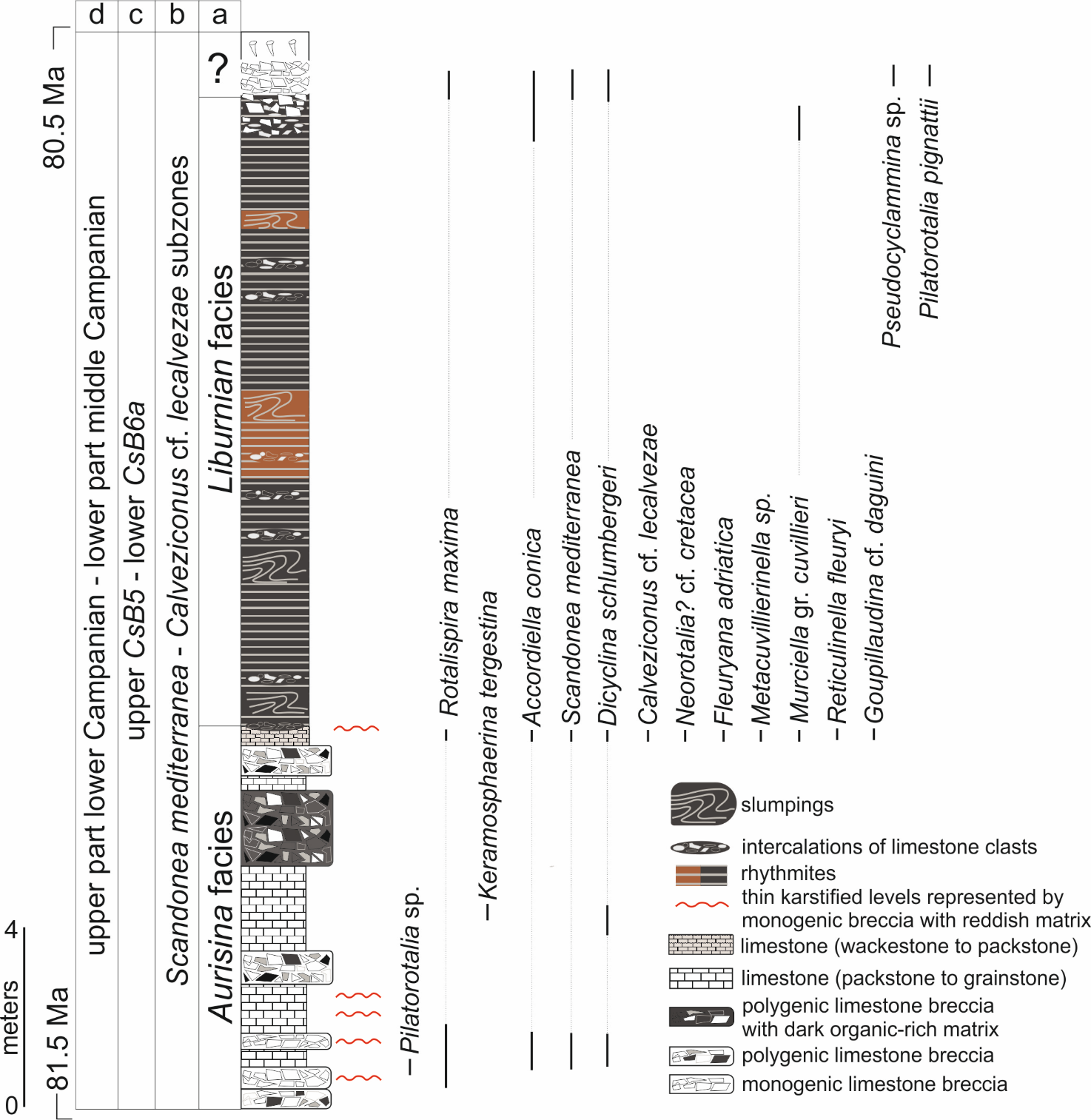
**Figure S1.** **Stratigraphic log.** **Lithostratigraphy, biostratigraphy and chronostratigraphy of the drilled core at the dinosaur site integrated with field data for the uppermost part of the lithologic column; reinterpreted from Palci (2003). a. Lithostratigraphy; b. subzones of Frijia et al., (2015); c. biozones of Fleury (2016); d. chronostratigraphic attribution referable to the entire lithostratigraphic succession.**


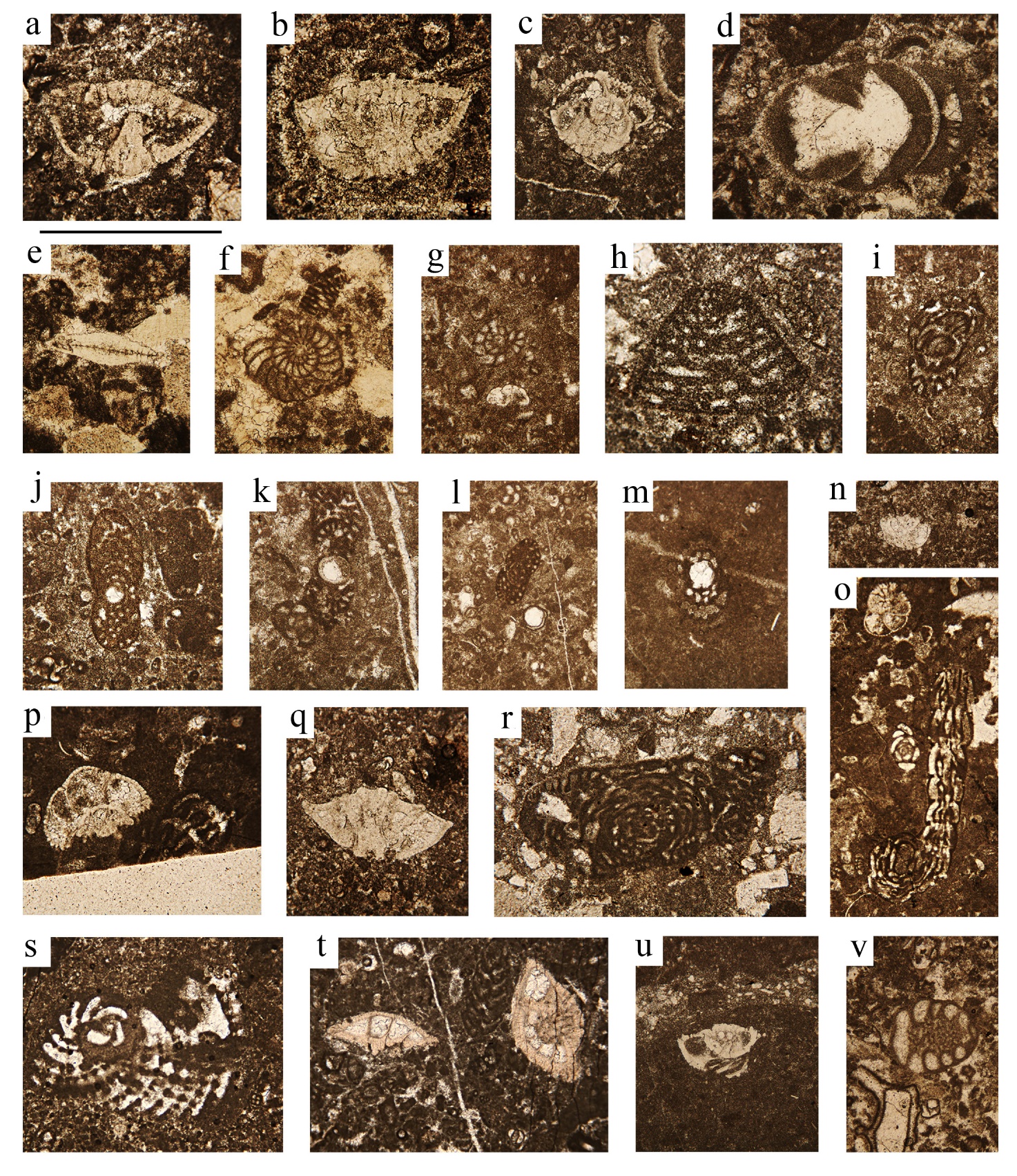
**Figure S2. Benthic foraminifera recovered from the studied core and field survey.** **Scale bar 1.6 mm for picture O, S, T, V; 1 mm for all the other pictures A. *Pilatorotalia* sp. B. *Neotoralia*? cf. *cretacea*. C. *Rotalispira* cf. *maxima*. D. *Scandonea mediterranea*. E, F. *Goupillaudina* cf. *daguini*. G. *Fleuryana adriatica*. H. *Calveziconus* cf. *lecalvezae*. I, M, O, R. *Murciella* gr. *cuvillieri*. The specimens in O and R have been recovered into a clastic-rich layer on top of the rhythmites succession. J-L. *Metacuvillierinella* sp. N. *Rotalispira scarsellai*. P. *Rotalispira* cf. *maxima* and *Murciella* gr. *cuvillieri*. Q, T. *Neotoralia*? cf. *cretacea.* S. *Murgella lata*. U. *Rotalispira* *maxima.***


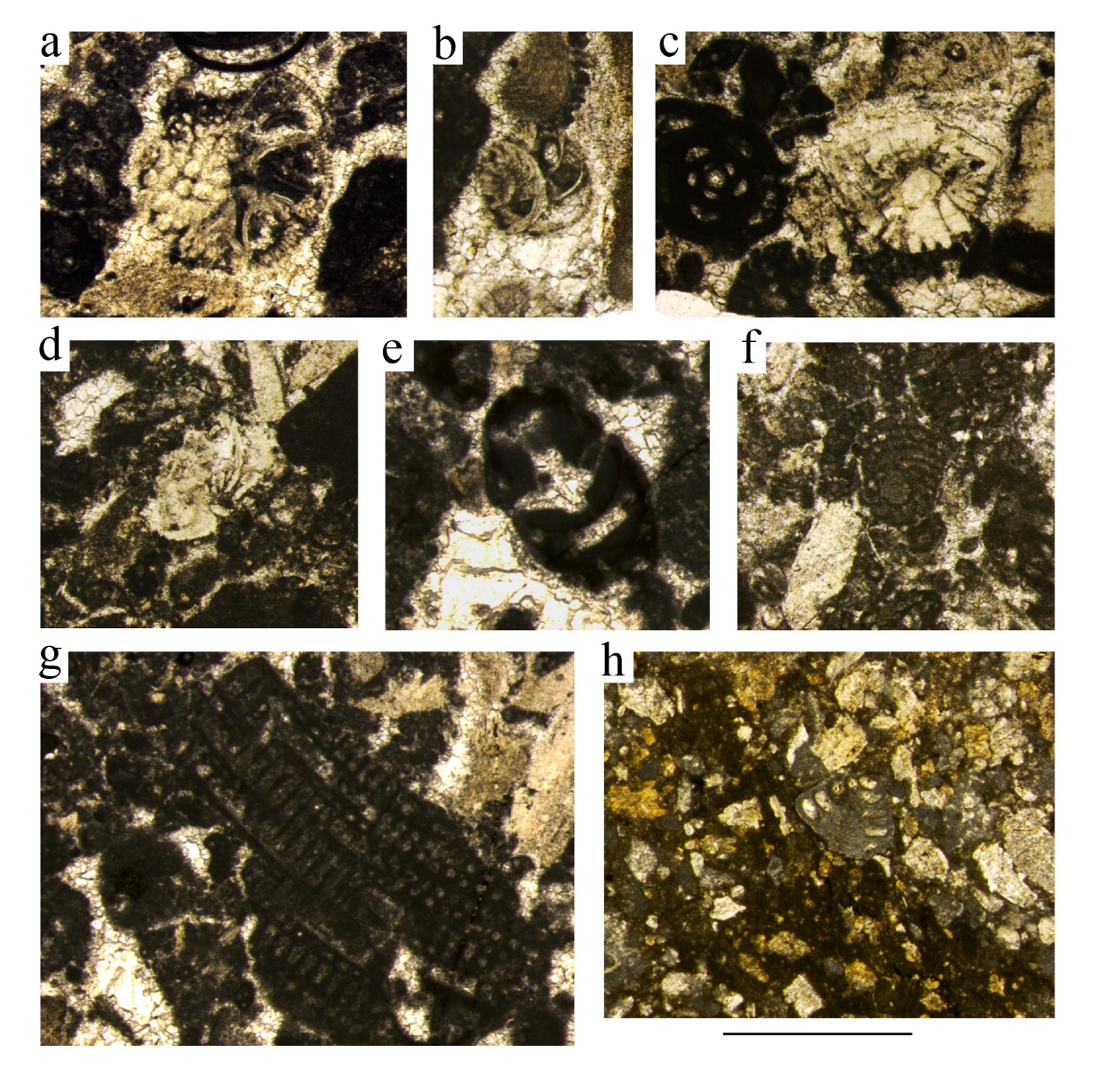


**Fig. S3. Benthic foraminifera recovered in a clastic layer at the top of rhythmites succession.** **Scale bar 1 mm for all the figured specimens. A, B. *Rotalispira maxima*. C. *Pilatorotalia pignattii*. D. *Rotalispira scarsellai*. E. *Scandonea mediterranea*. F. *Pseudocyclammina* sp. G. *Dicyclina schlumbergeri*. H. Isolated *Accordiella conica* floating into the rhythmites matrix along with other limestone clasts.**


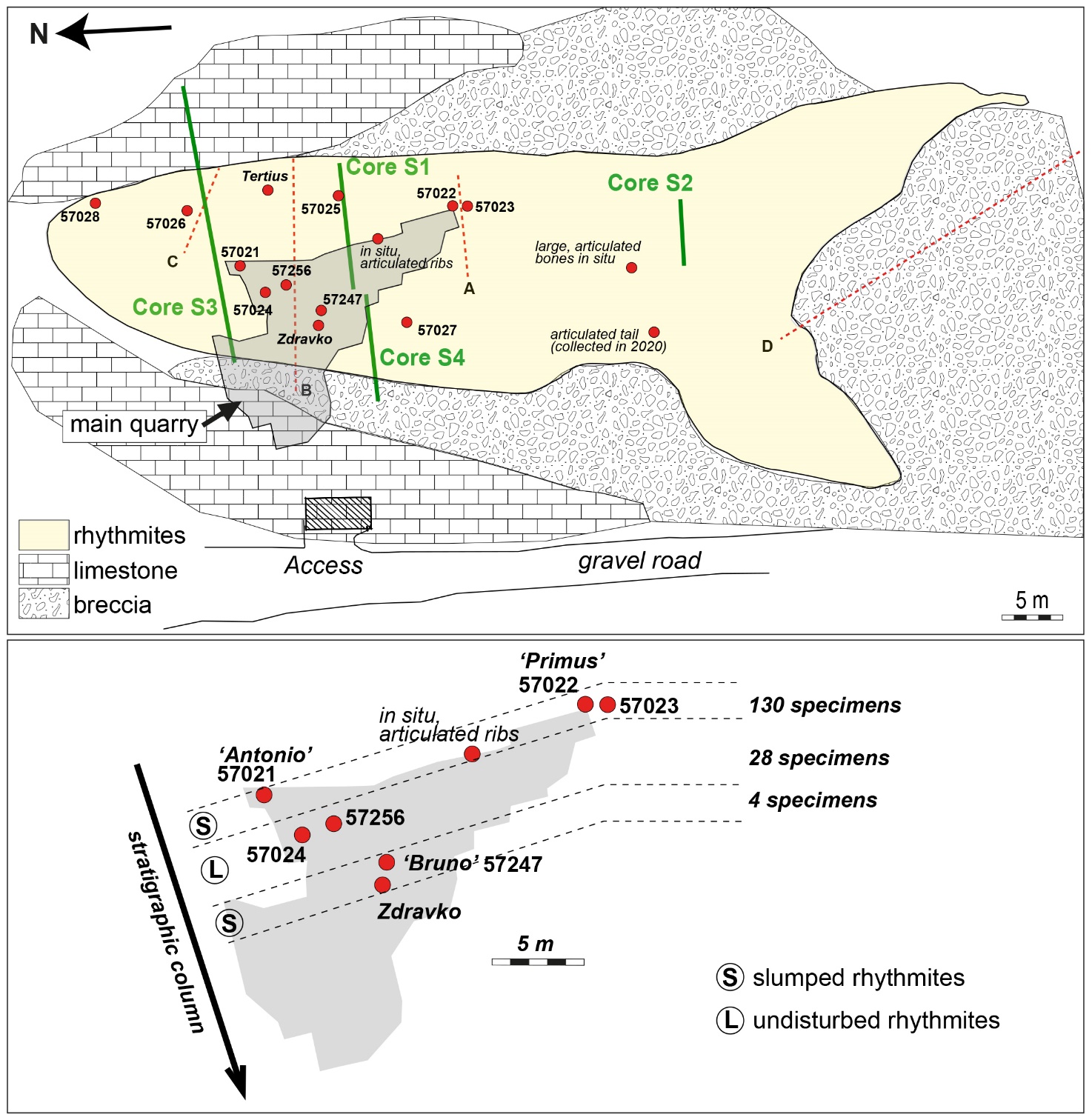
**Fig. S4. Simplified geological map of the VdP site with the exact location of hadrosauroid elements discussed in the main text assigned to *T*. *insularis*. The light-grey area delimits the main, polished quarry area. Repositioning of all fossils extracted from the 1990’s and deposited in Trieste allowed for precise definition of high- vs low-density fossil interval. Map produced by one of us (Federico Fanti) via Adobe Illustrator 2020 (24.0).**


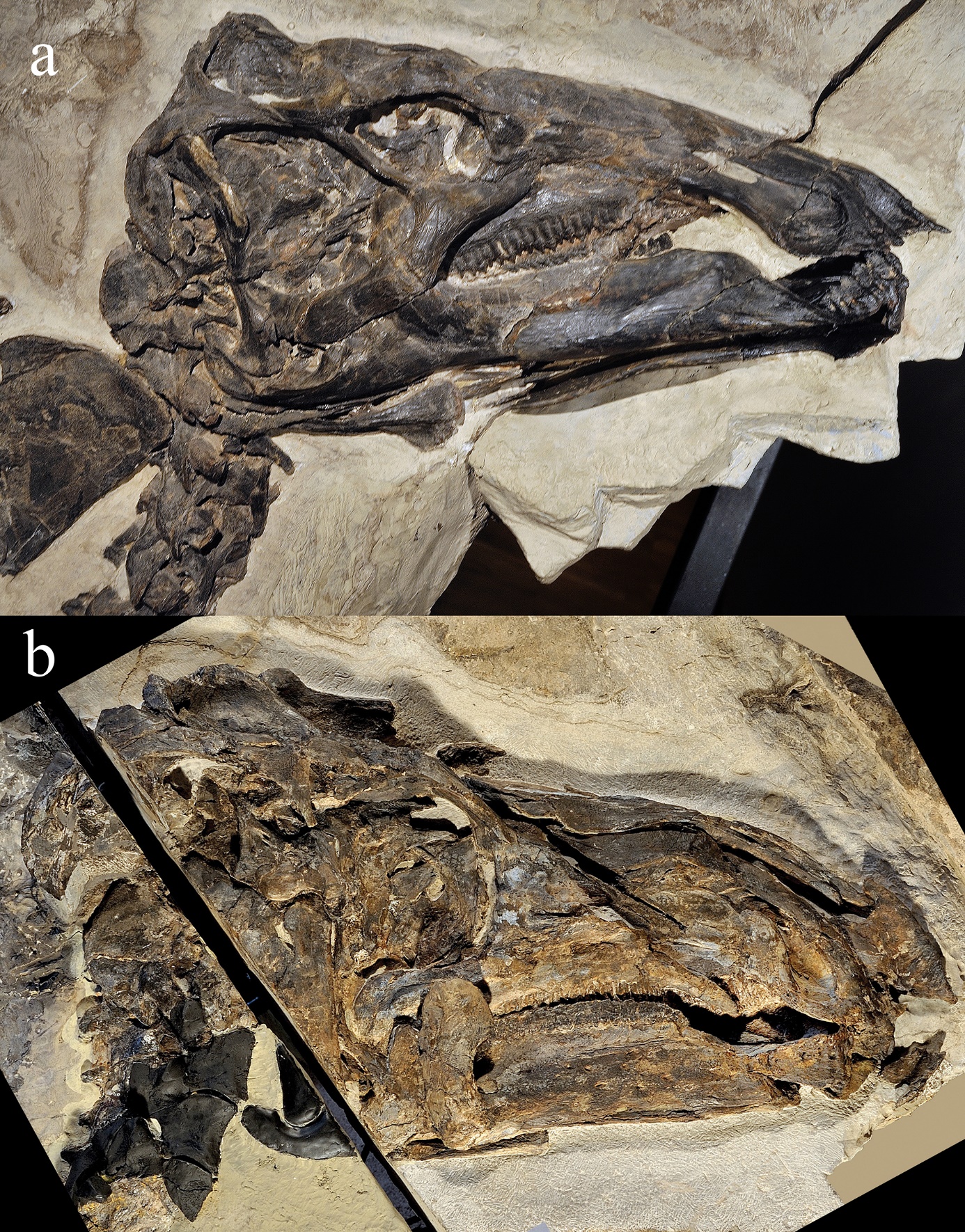
**Figure S5.** **Skulls from the holotype (SC 57201) of *Tethyshadros insularis* (a) and the herein newly described SC 57247 individual. Crania not to scale (check Fig. S6 for relative proportions between individual skulls).**


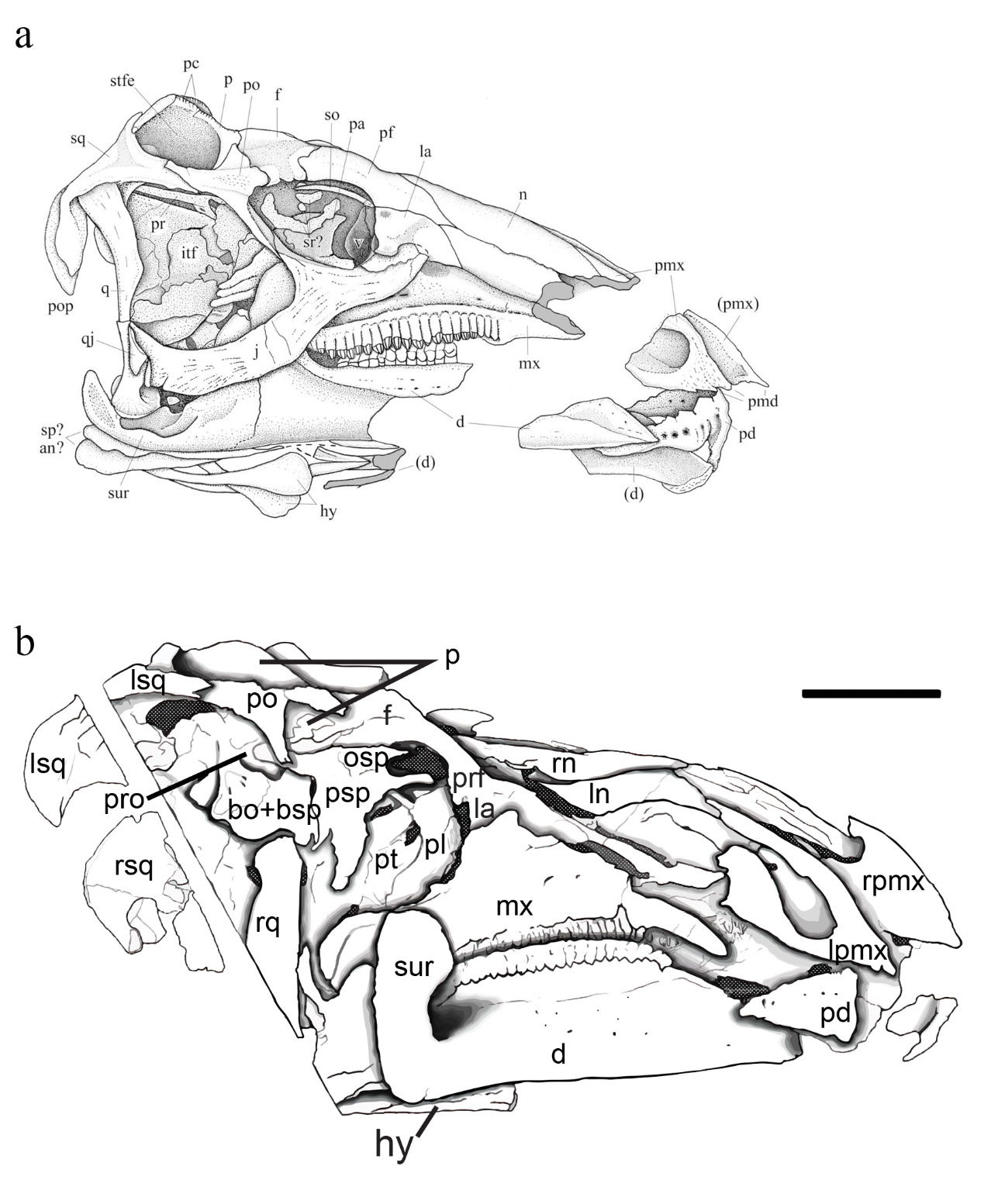


**Figure S6.** **Interpretative sketches from the skull of the holotype of *Tethyshadros* (modified from Dalla Vecchia**^29^**) and SC 57247. Scalebar is 10 cm. Anatomical abbreviations: l, left; r, right; sq, squamosal, pro, prootic; po, postorbital; f, frontal; q, quadrate; la, lacrimal; bo, basioccipital; bsp, basipshenoid; sur, surangular; hy, hyoid; d, dentary; mx, maxilla; pd, predentary; pmx, premaxilla; n, nasal; prf, prefrontal; f, frontal.**


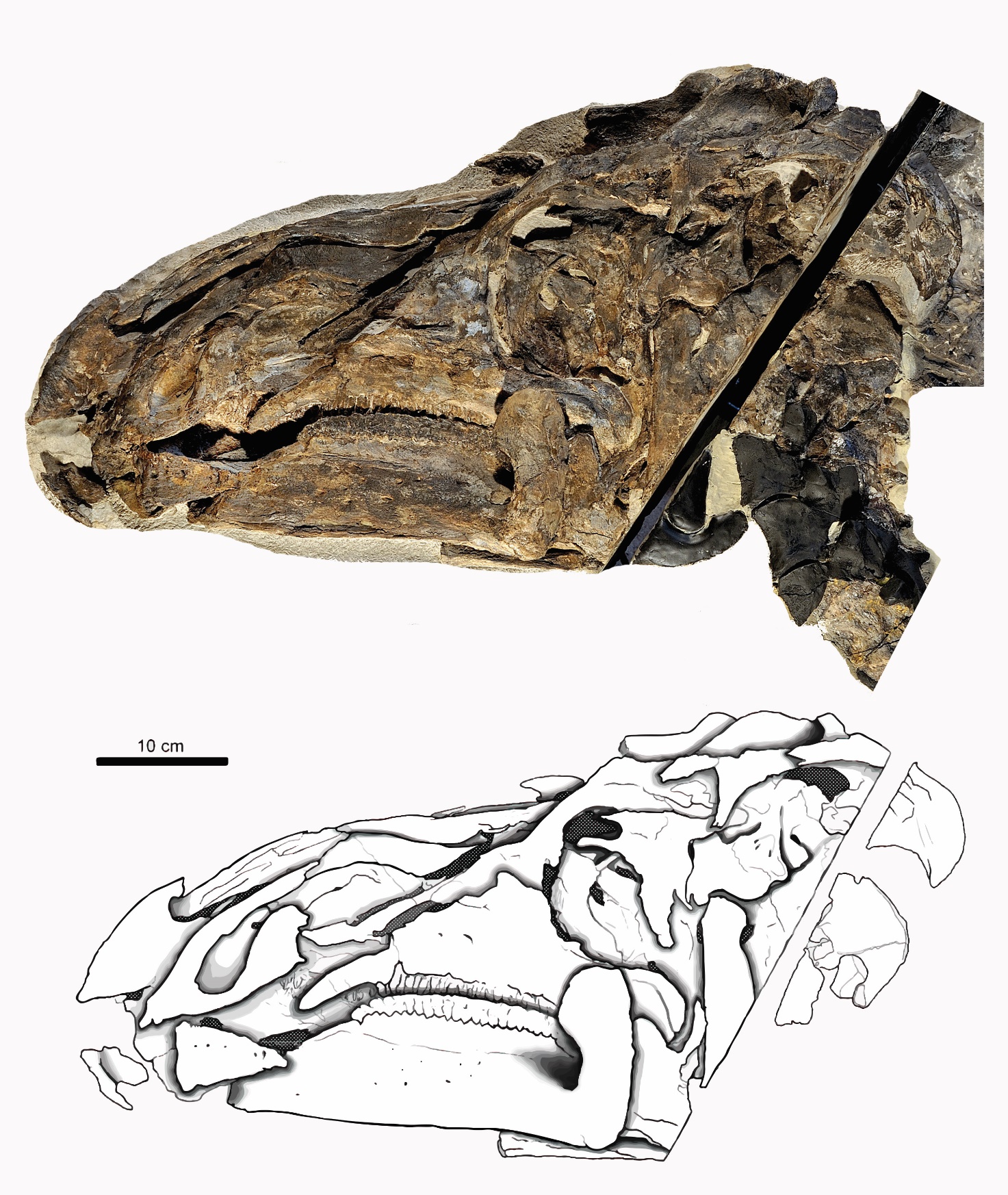
**Figure S7. Skull of SC 57247 and interpretative drawing as included in Fig. 2 of the main text.**


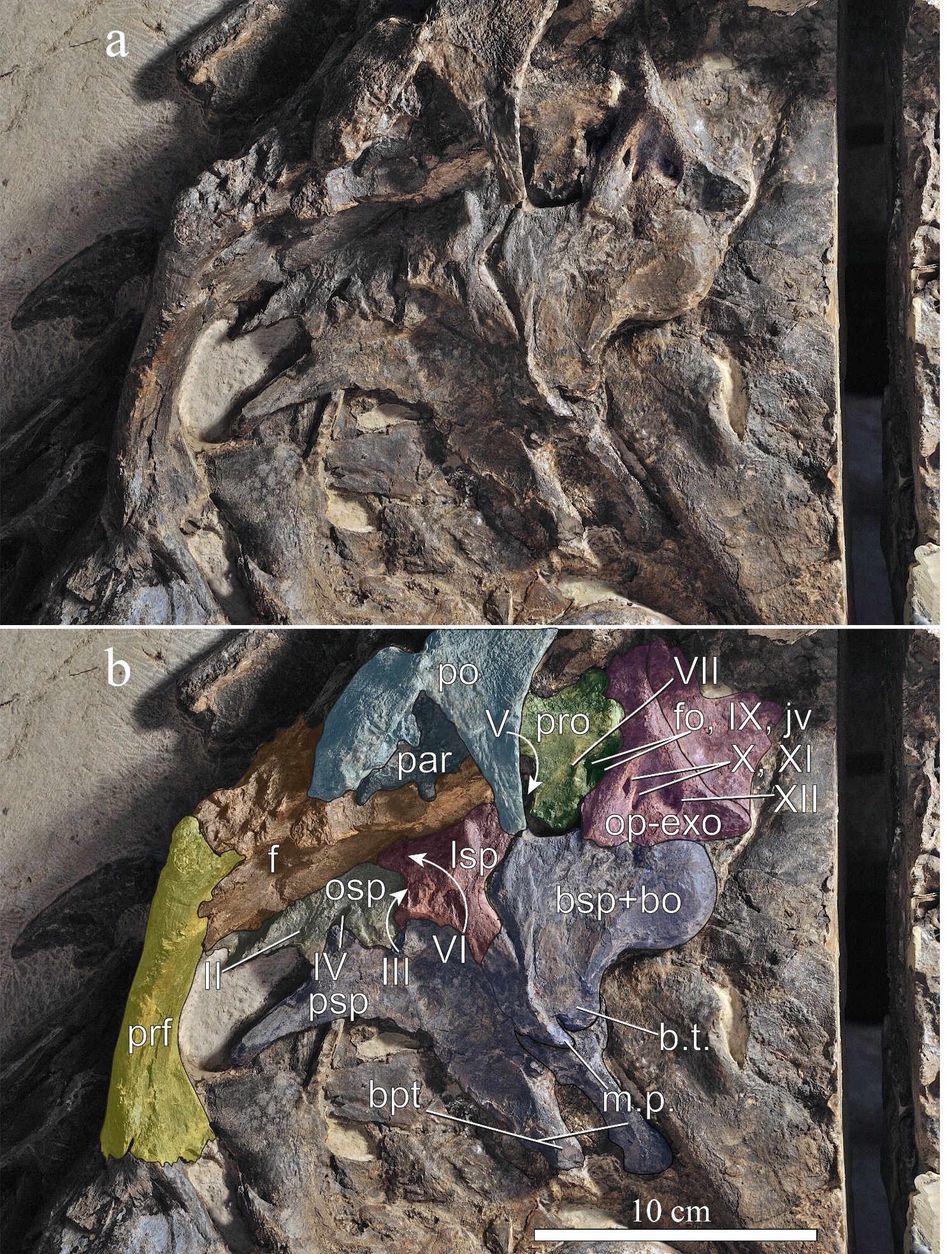


**Figure S8.** **Neurocranium of *Tethyshadros insularis* from SC 57247 (a) and labelled (b) with some elements of the dermatocranium. Anatomical abbreviations, sq, squamosal, pro, prootic; po, postorbital; f, frontal; bo, basioccipital; bsp, basipshenoid; prf, prefrontal; f, frontal; par, parietal; bpt, basipterygoid processes of the basisphenoid; m.p. median process of the basisphenoid; b.t., basitubera; lsp, laterosphenoid; jv, exit of the jugular vein; fo, fenestra ovalis; op-exo, opistothic-exoccipital complex; Cranial nerve numbers follow roman enumeration. Scale bar in Fig. 2 of the main text.**


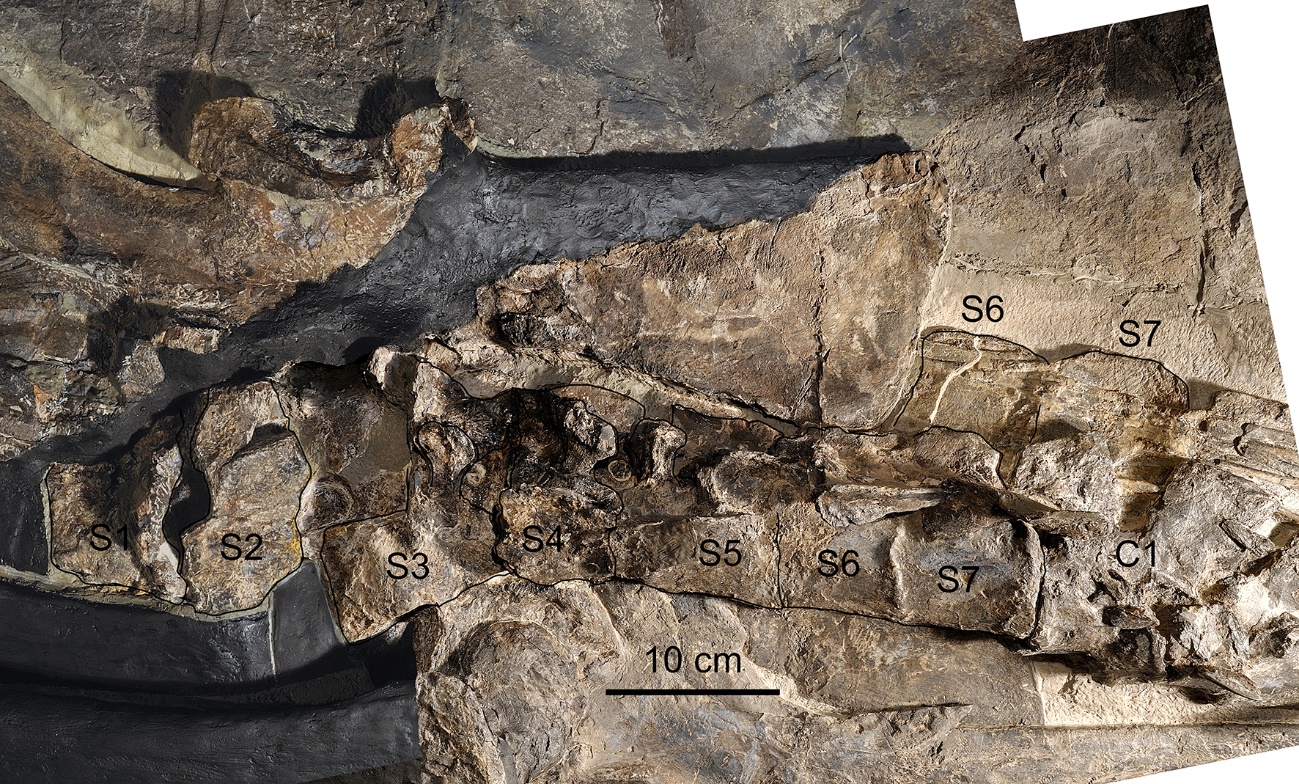


**Figure S9.** **Sacrum of SC 57247. Abbreviations: S, sacral vertebra; C, caudal vertebra.**


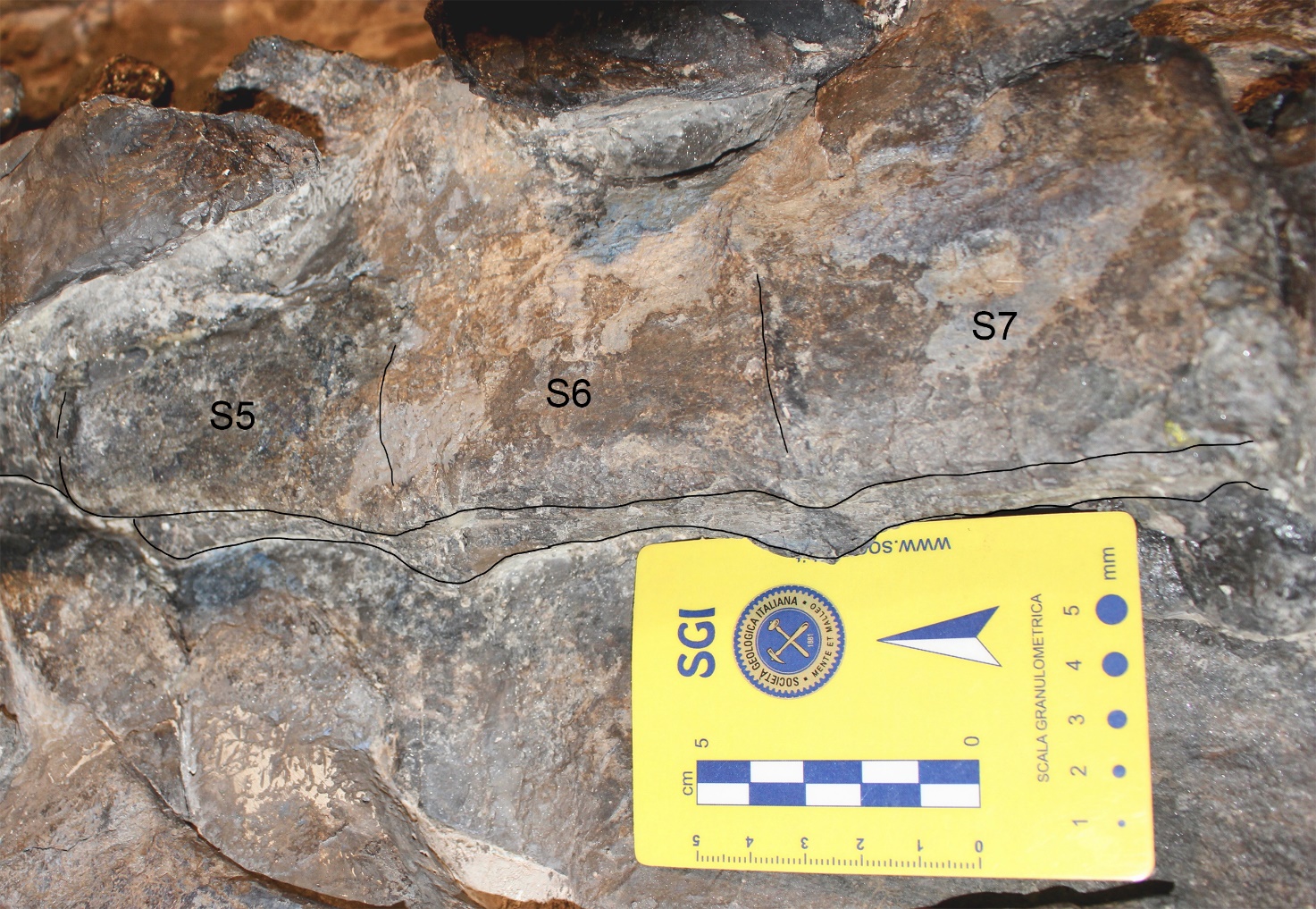
**Figure S10.** **Posterior portion of the sacrum in SC 57247 showing the posterior termination of this anatomical district and the proximal region of the tail. Abbreviation: S, sacral vertebra.**


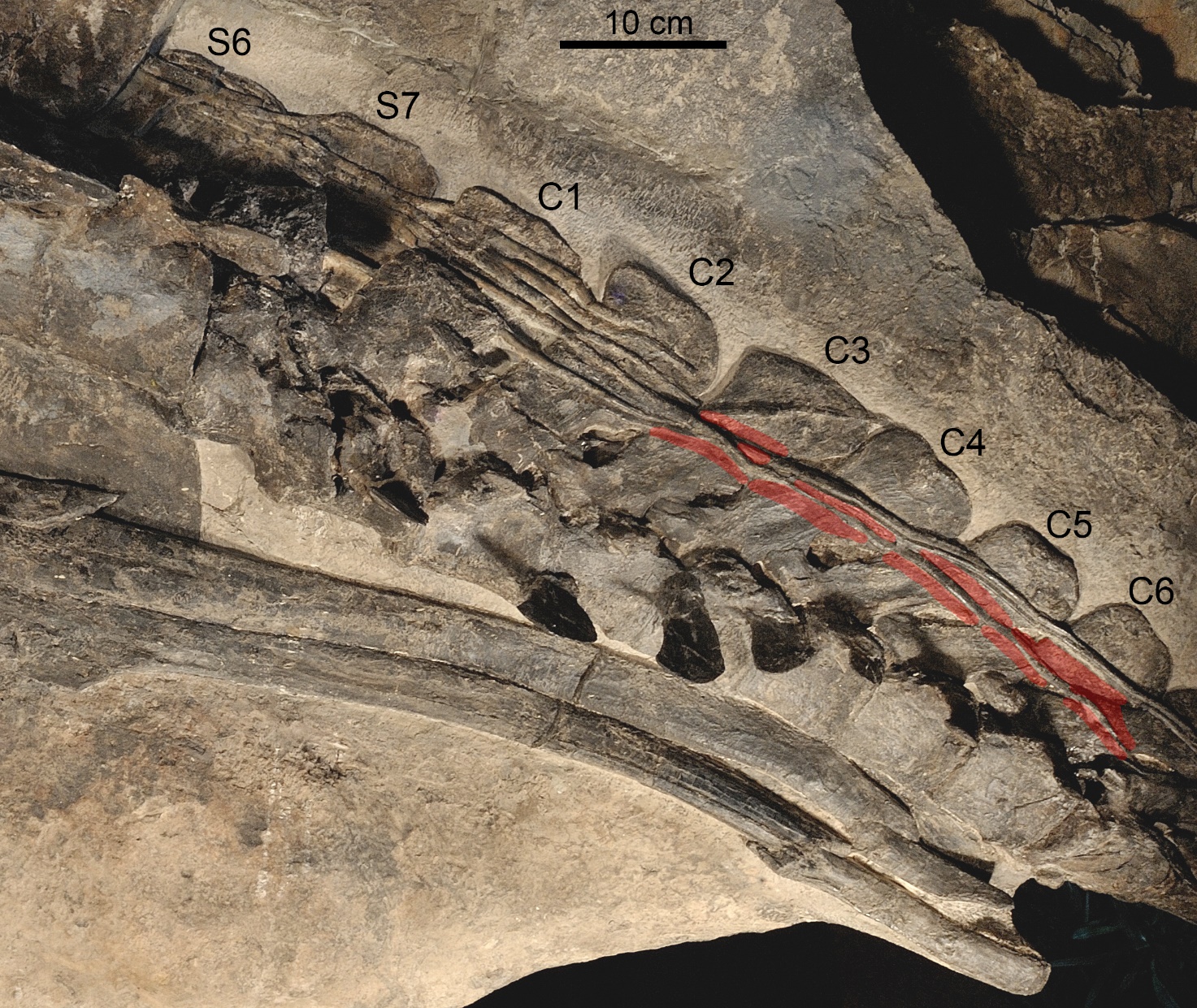
**Figure S11.** **Proximal caudal vertebrae in SC 57247, highlighting in red the ossified tendons. Abbreviations: S, sacral vertebra; C, caudal vertebra.**


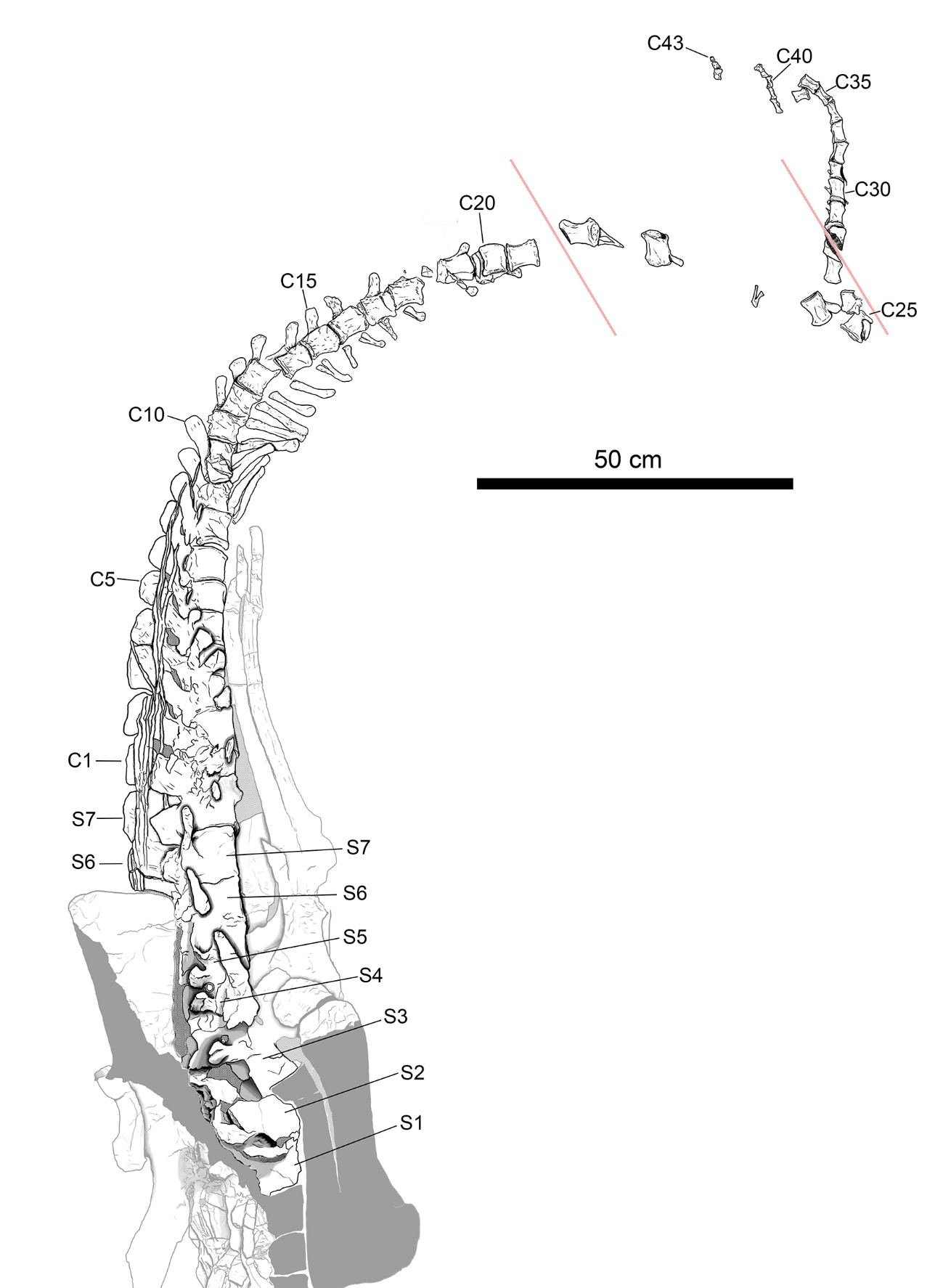


**Figure S12.** **Interpretative sketch of the sacro-caudal region in SC 57247. Abbreviations: S, sacral vertebra; C, caudal vertebra. Area shaded in grey represents elements not preserved or restored in the specimen. Red lines represent artificial cuts made during the extraction of the specimen from the field.**


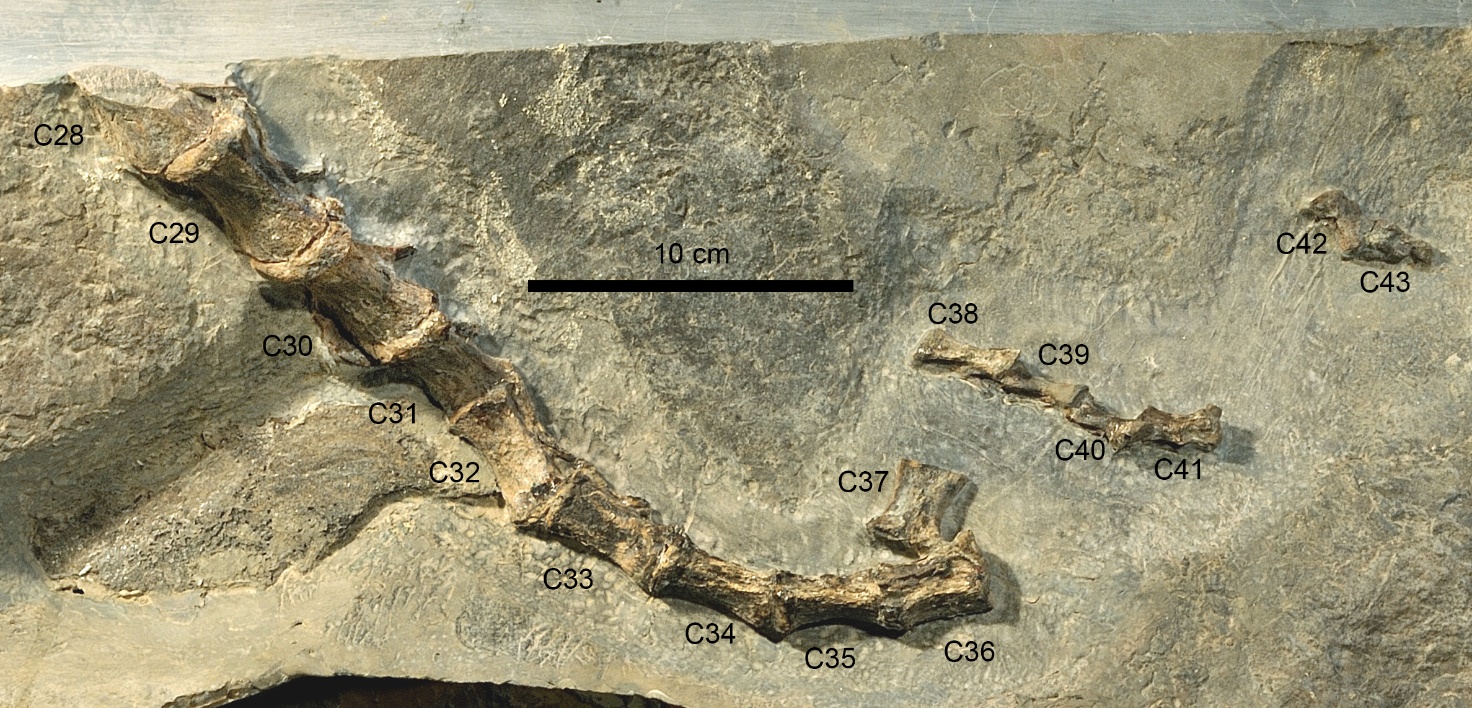
**Figure S13.** **Distal caudal vertebrae in SC 57247. Abbreviations: C, caudal vertebra.**


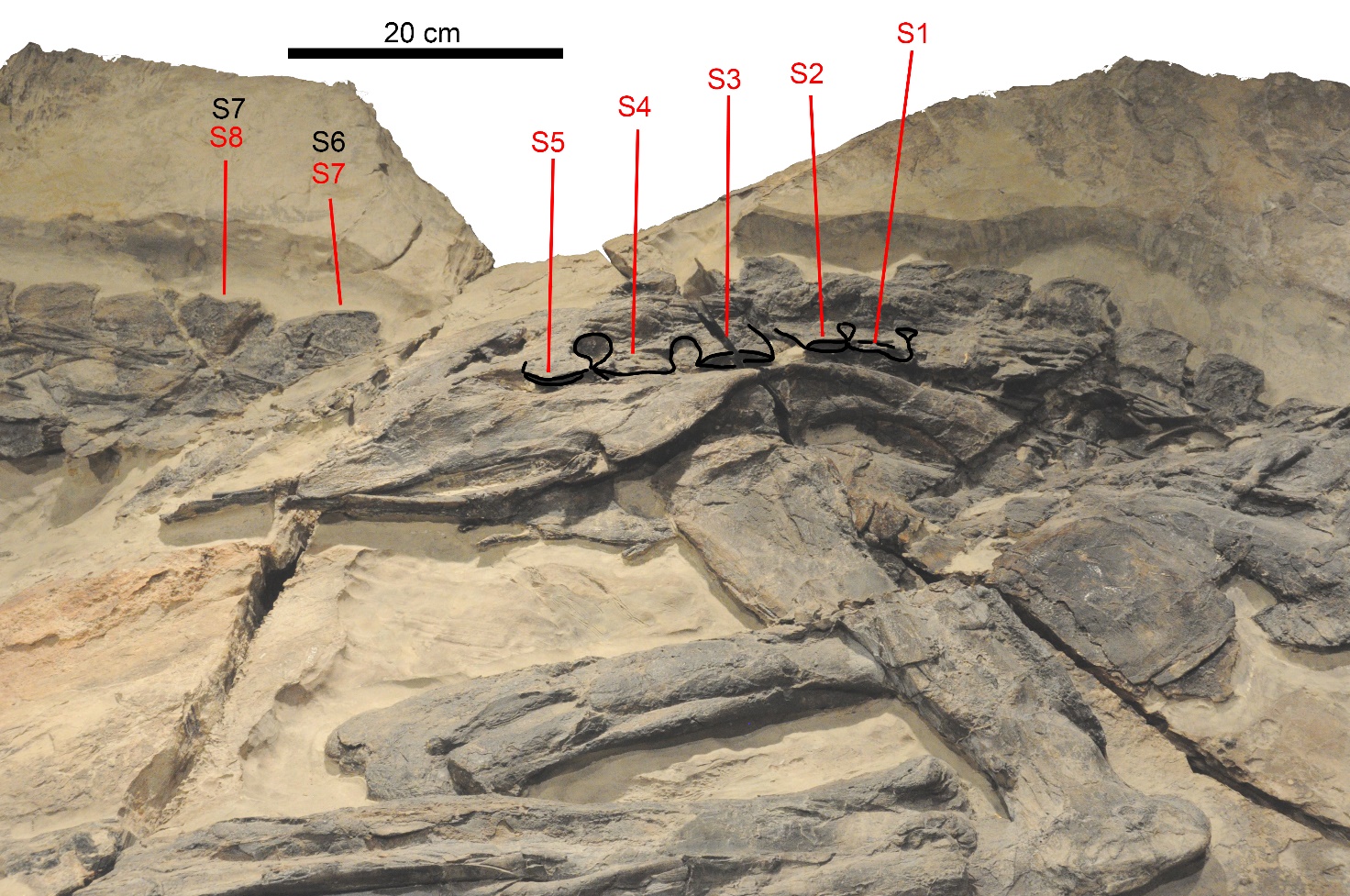
**Figure S14.** **Pelvic region and proximal caudal vertebrae in SC 47021. Labels in red represent older interpretations (Dalla Vecchia, 2008, 2009) on this caudal series, while in black is reported the one followed in this study. Abbreviations: S, sacral vertebra; C, caudal vertebra.**


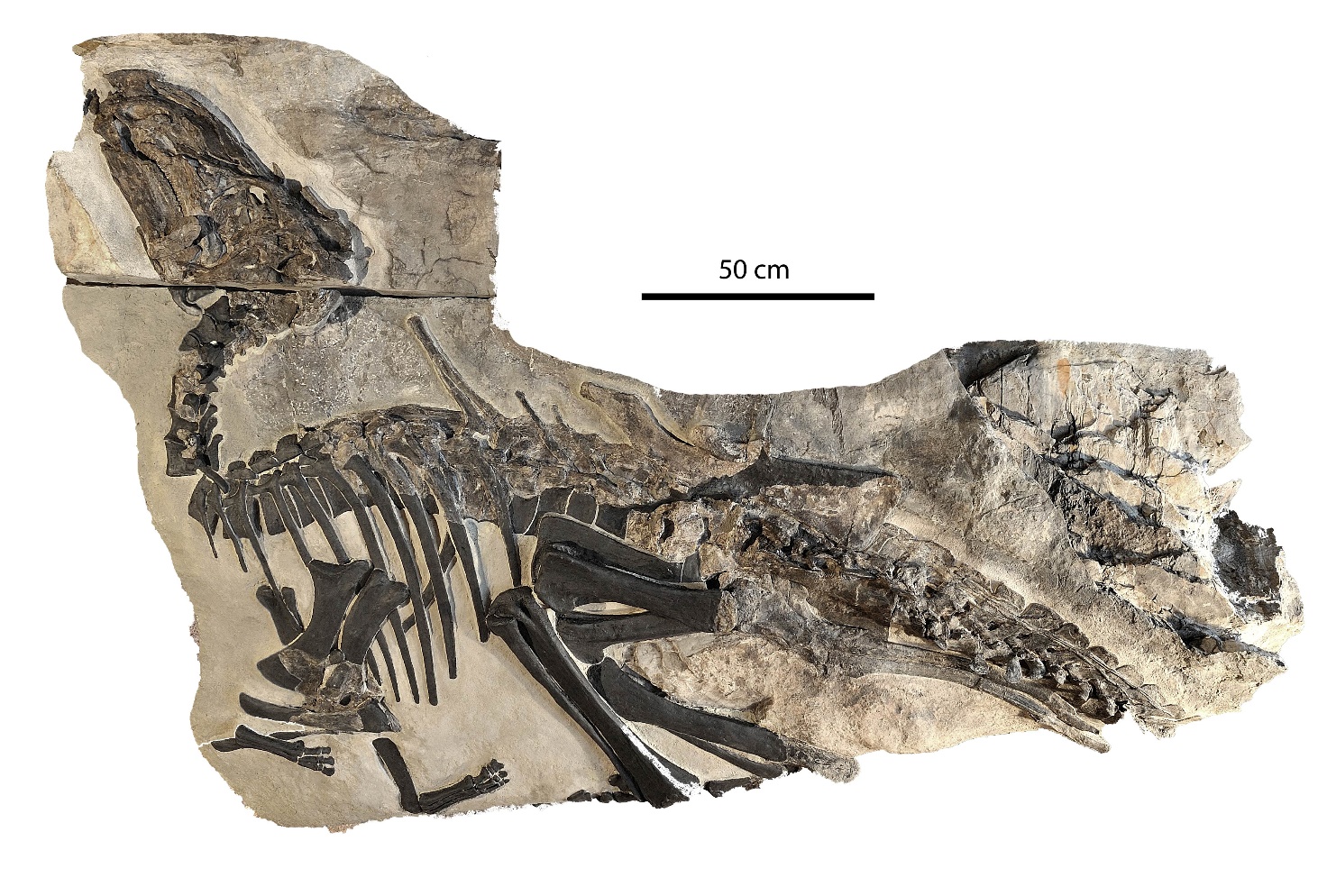
**Figure S15.** **Skeleton of SC 57247 exposed from the side showing the majority of the preserved elements (cranial and postcranial).**


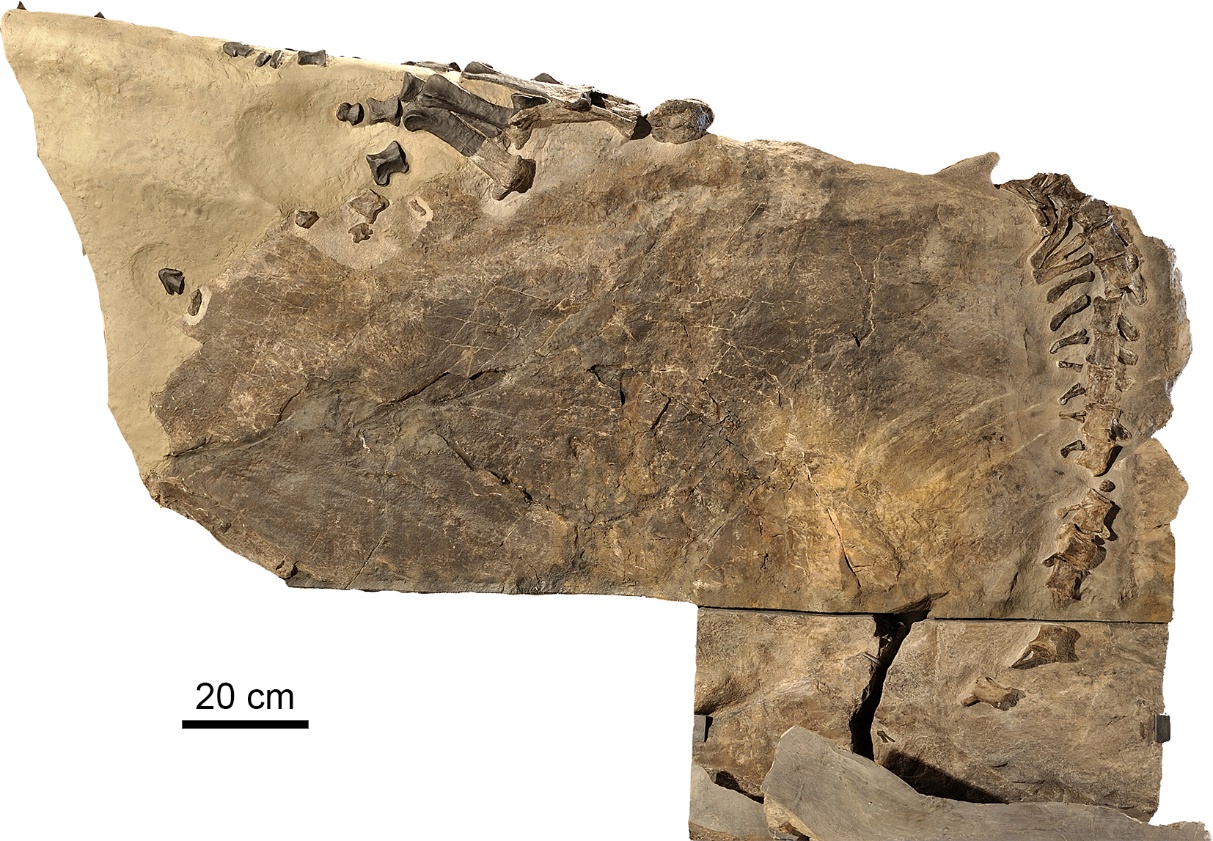


**Figure S16.** **Skeleton of SC 57247 exposed from the side showing the remnant preserved elements like the mediodistal portion of the tail and some distal elements of the posterior limb.**


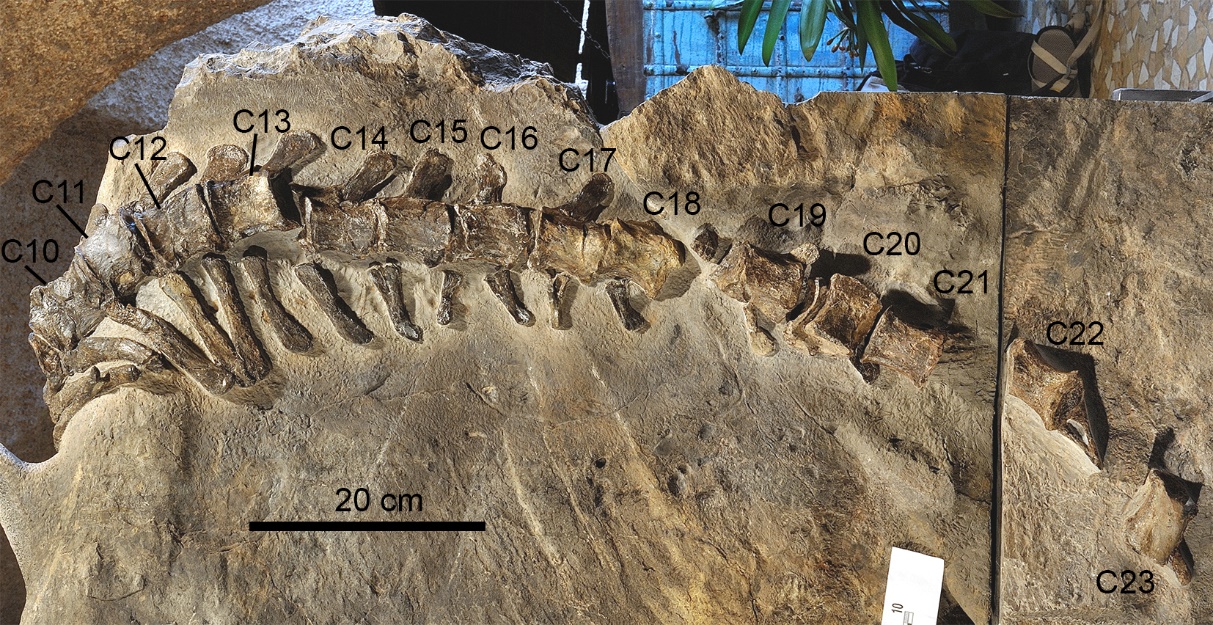


**Figure S17.** **Mid caudal series of SC 57247. Abbreviations: C, caudal vertebra.**


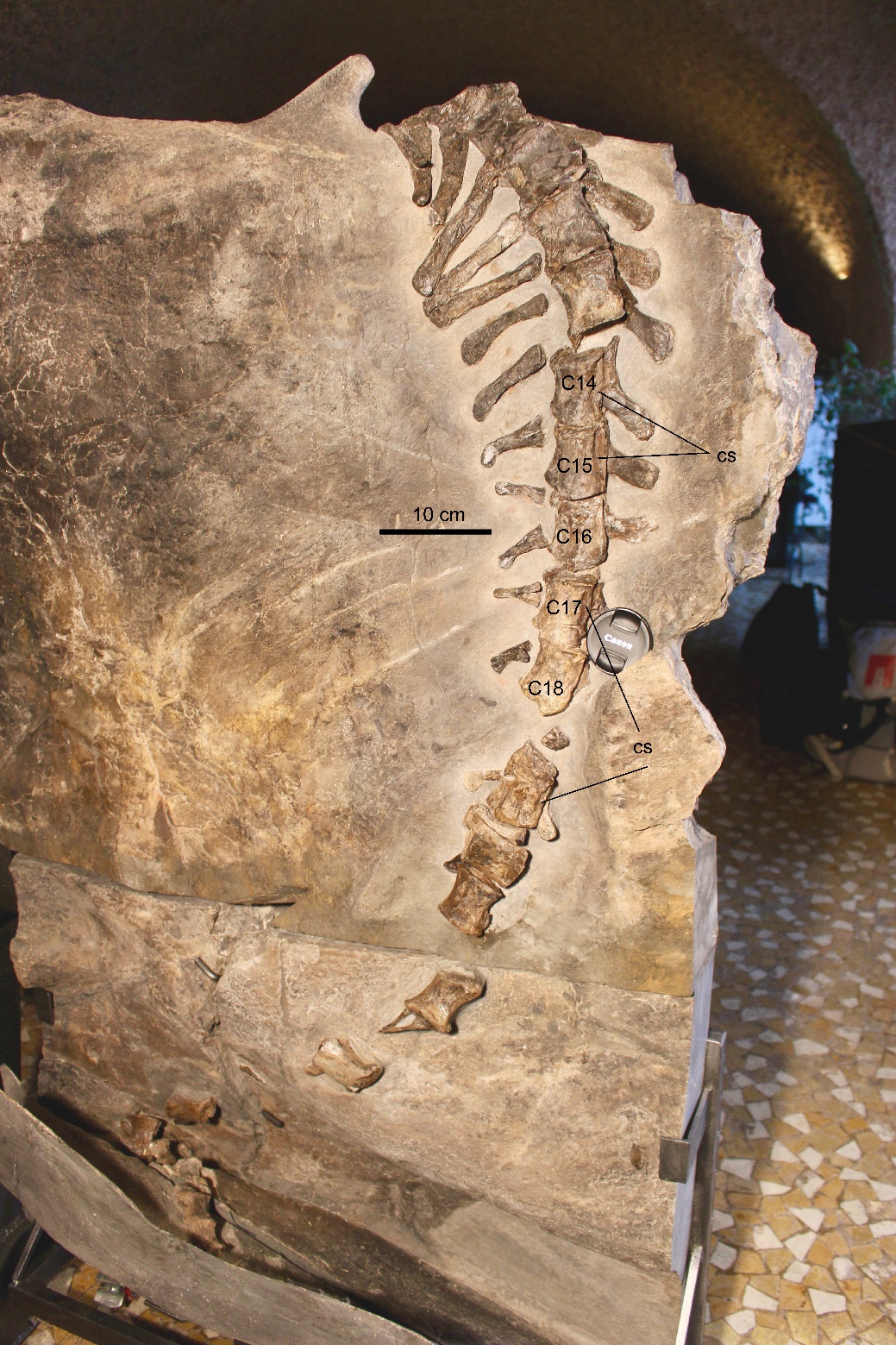


**Figure S18.** **Medio-distal caudal series of SC 57247. Anatomical abbreviations: C, caudal vertebra; cs, caudal sulcus.**


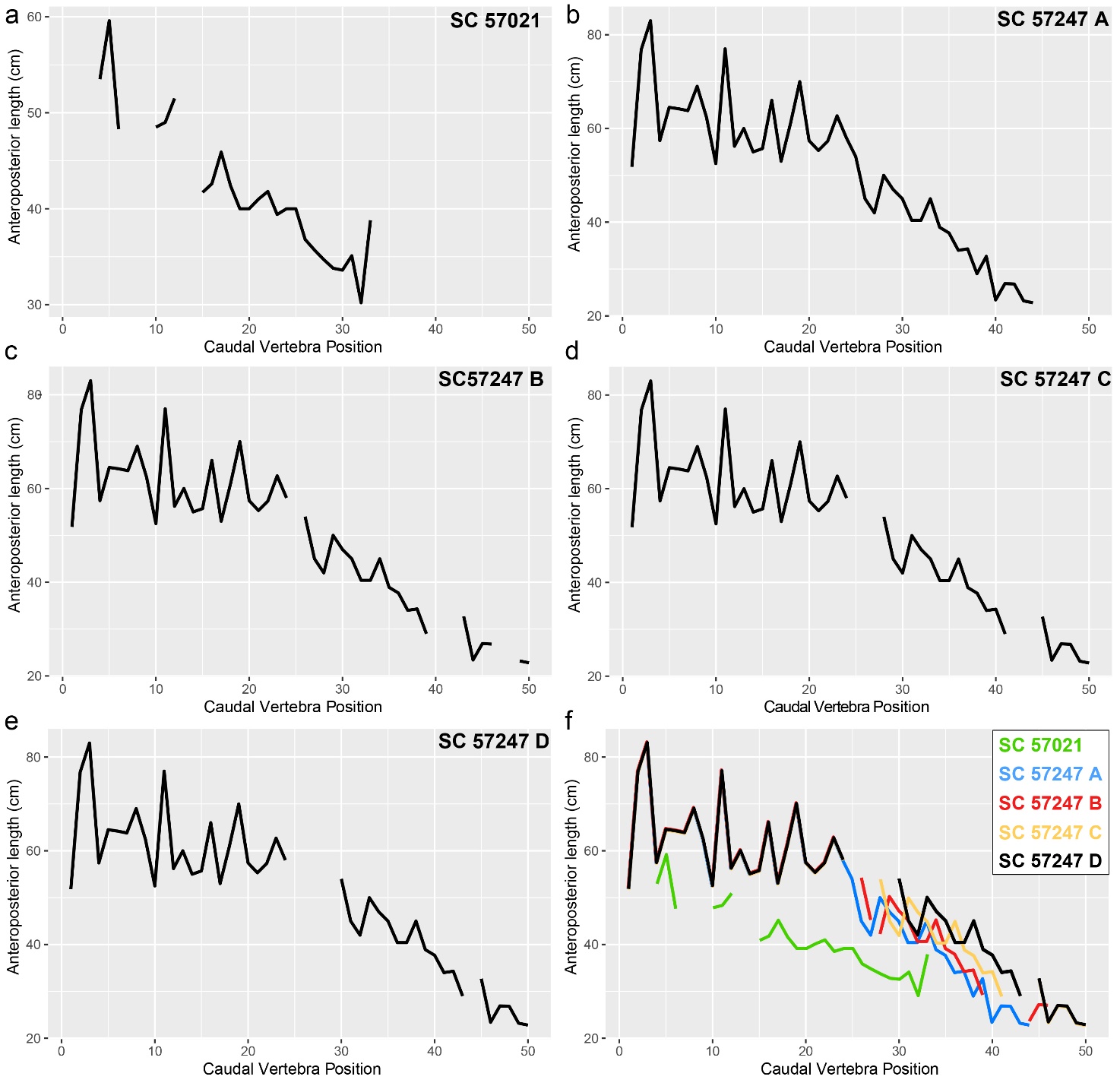
**Figure S19.** **Plot showing the anteroposterior length of the caudal centra in *Tethyshadros insularis* comparing the series in both SC 57021 and SC 57247. The most distal half of the tail in SC 57247 supports different interpretations depending on how the gaps in the series are filled. The interpretation in the series named A is the one followed in this study (see relevant anatomical description section here).**


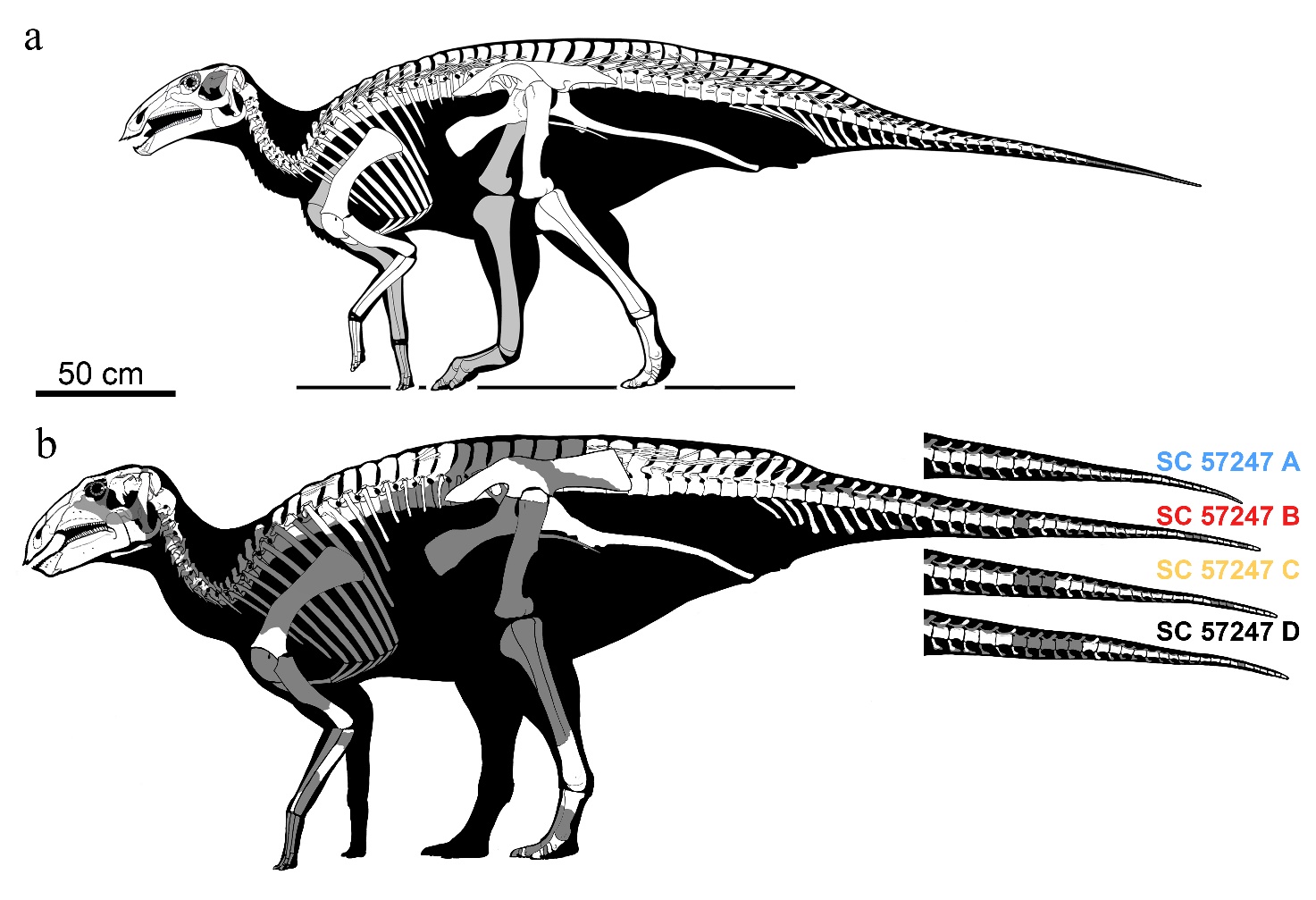
**Figure S20.** **Skeletal drawings restoring the anatomical interpretations for SC 57021 (a) and SC 57247 (b) and different scenarios of tail length as discussed in the text.**


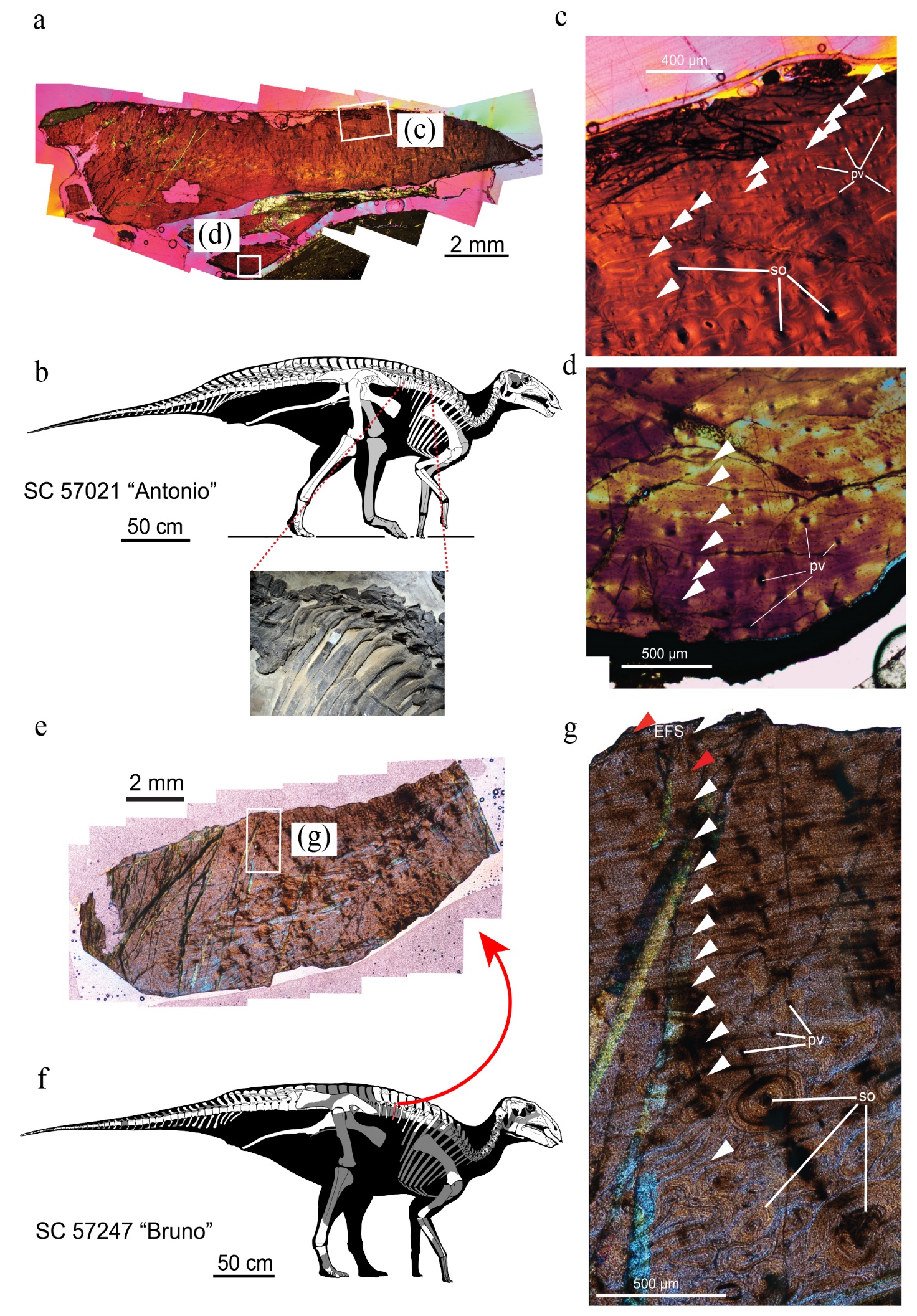


**Figure S21.** **Osteohistological sampling of SC 57021 (a–d) and SC 57247 (e-g). Higher details in the thin sections are reported in Fig. S22. Red fillings, arrows and dashed lines indicate the provenance of the thin sections. Abbreviations: pv, primary vascularity; so, secondary osteons; EFS, external fundamental system.**


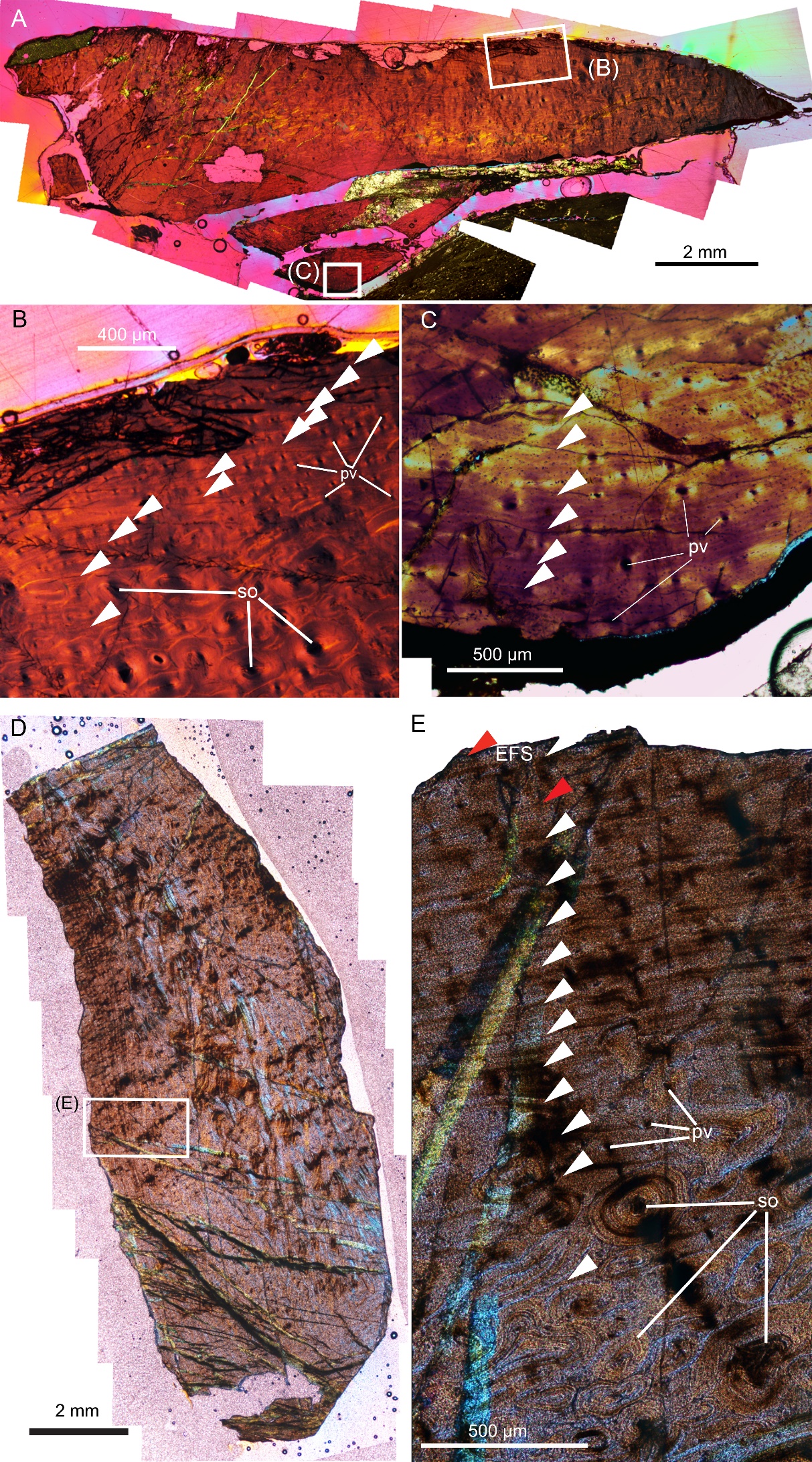


**Figure S22.** **Osteohistological thin sections of SC 57021 (A–C) and SC 57247 (D-E). Abbreviations: pv, primary vascularity; so, secondary osteons; EFS, external fundamental system.**


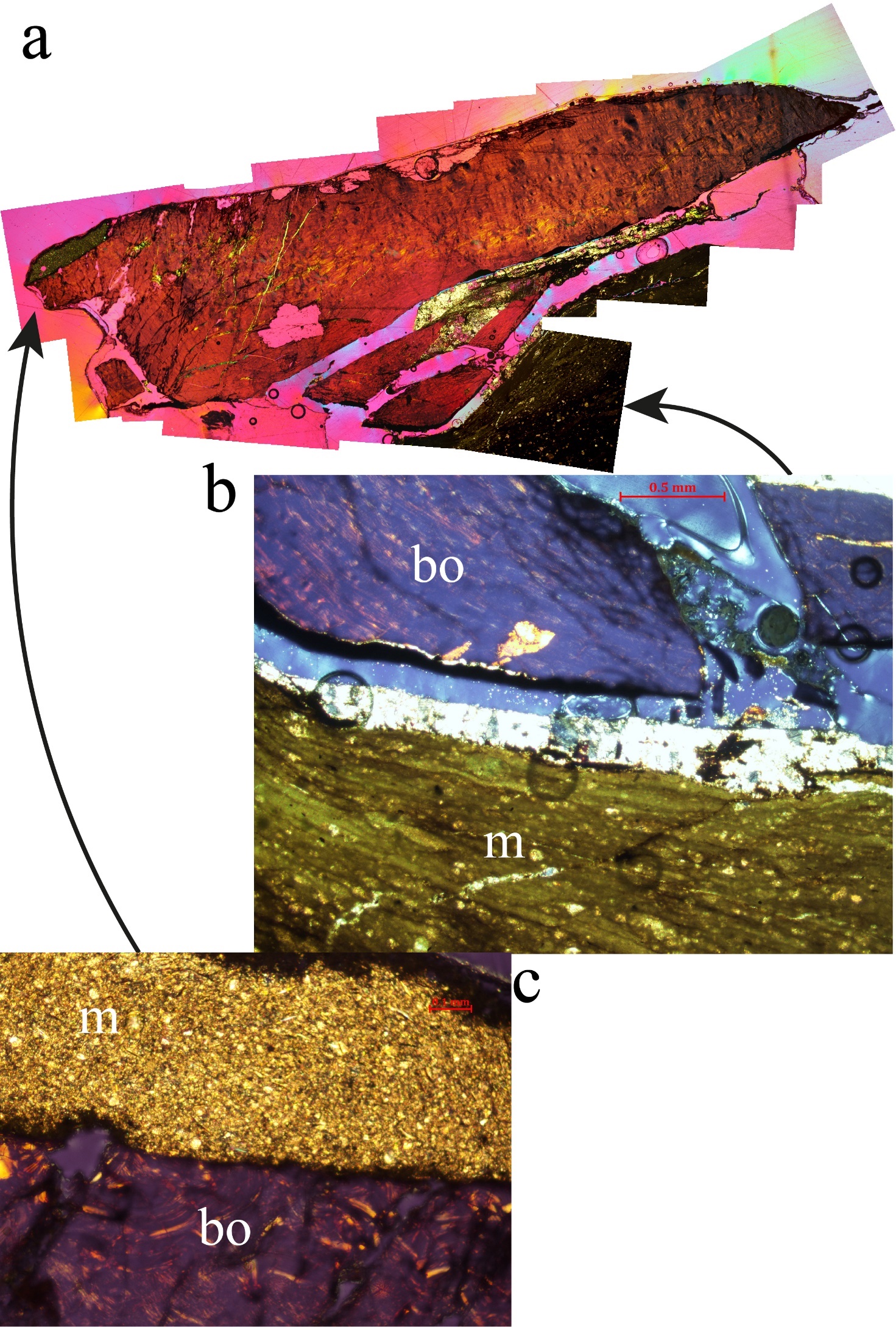


**Figure S23. Entire rib section of SC 57021 (a) emphasizing the contact between the bone tissue (bo) and the matrix (m - rithmytes in b, carbonatic in c), highlighting the preserved peripheral cortex in the holotypic specimen. Abbreviations: bo, bone; m, matrix.**


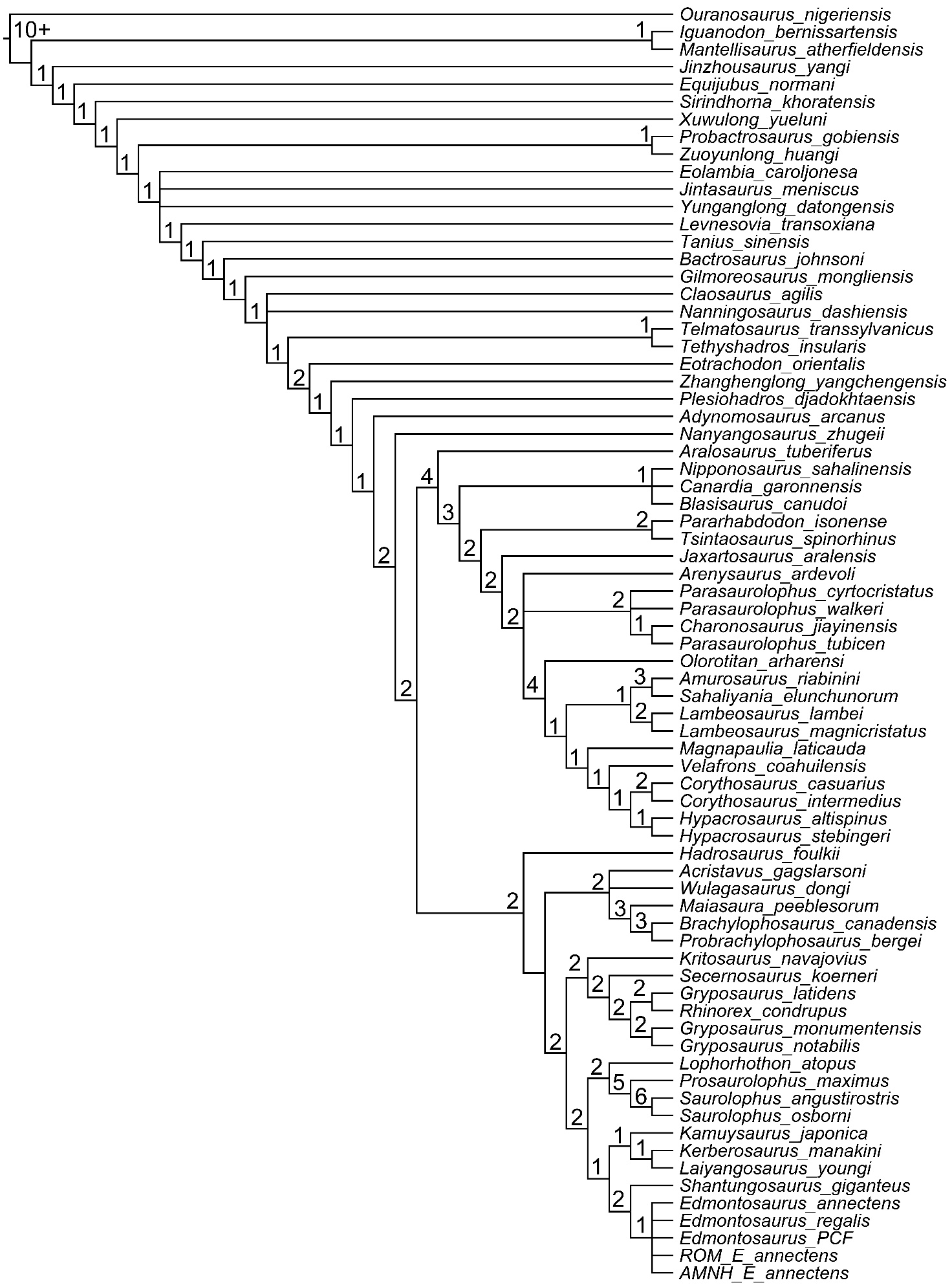
**Figure S24.** **Strict consensus of the taxon-based phylogenetic analysis showing the relative position of *Tethyshadros insularis* in Hadrosauriformes.**


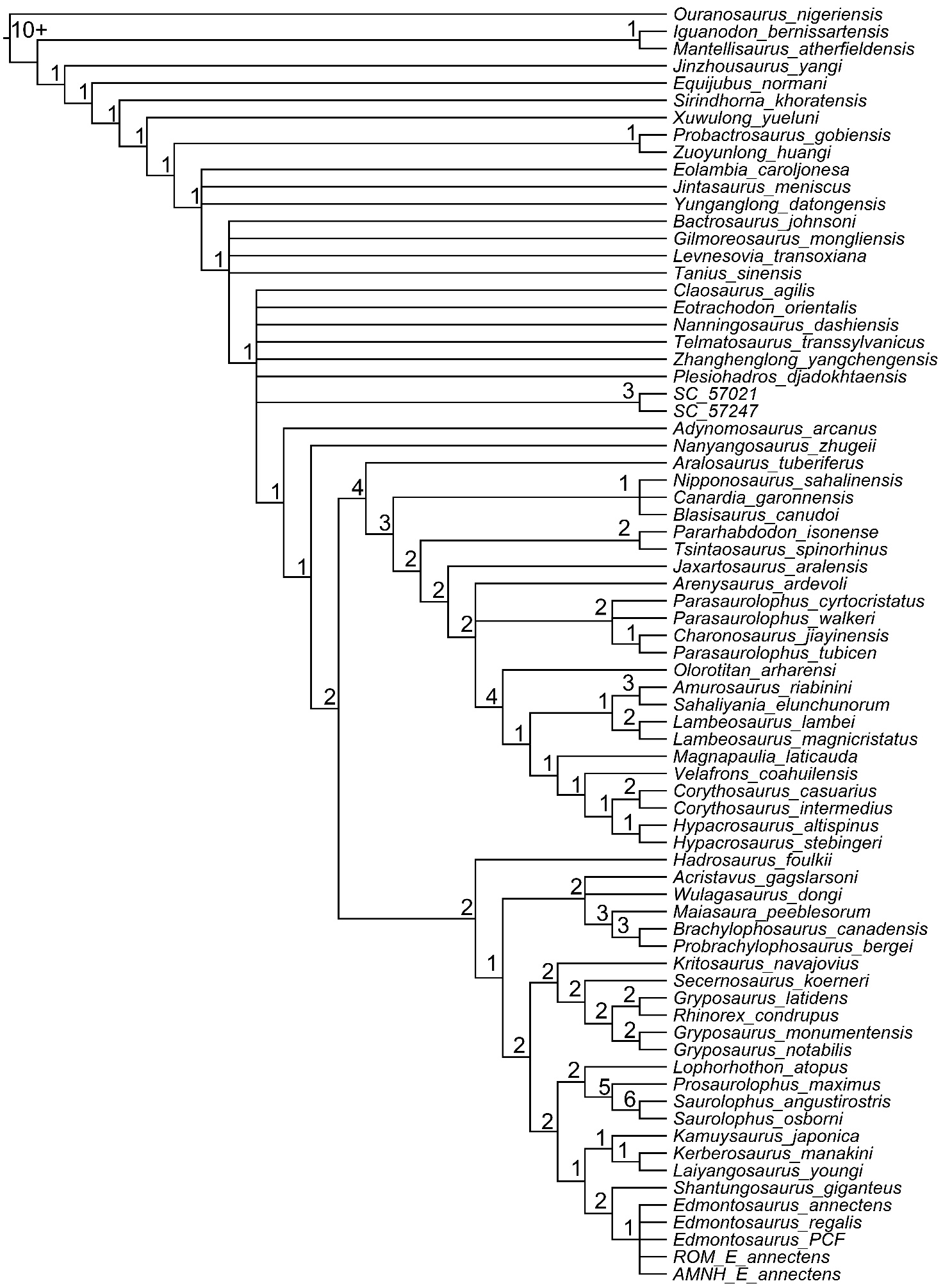
**Figure S25.** **Strict consensus of the individual-based phylogenetic analysis showing the relative position of both individuals of *Tethyshadros insularis* between each other and in Hadrosauriformes.**


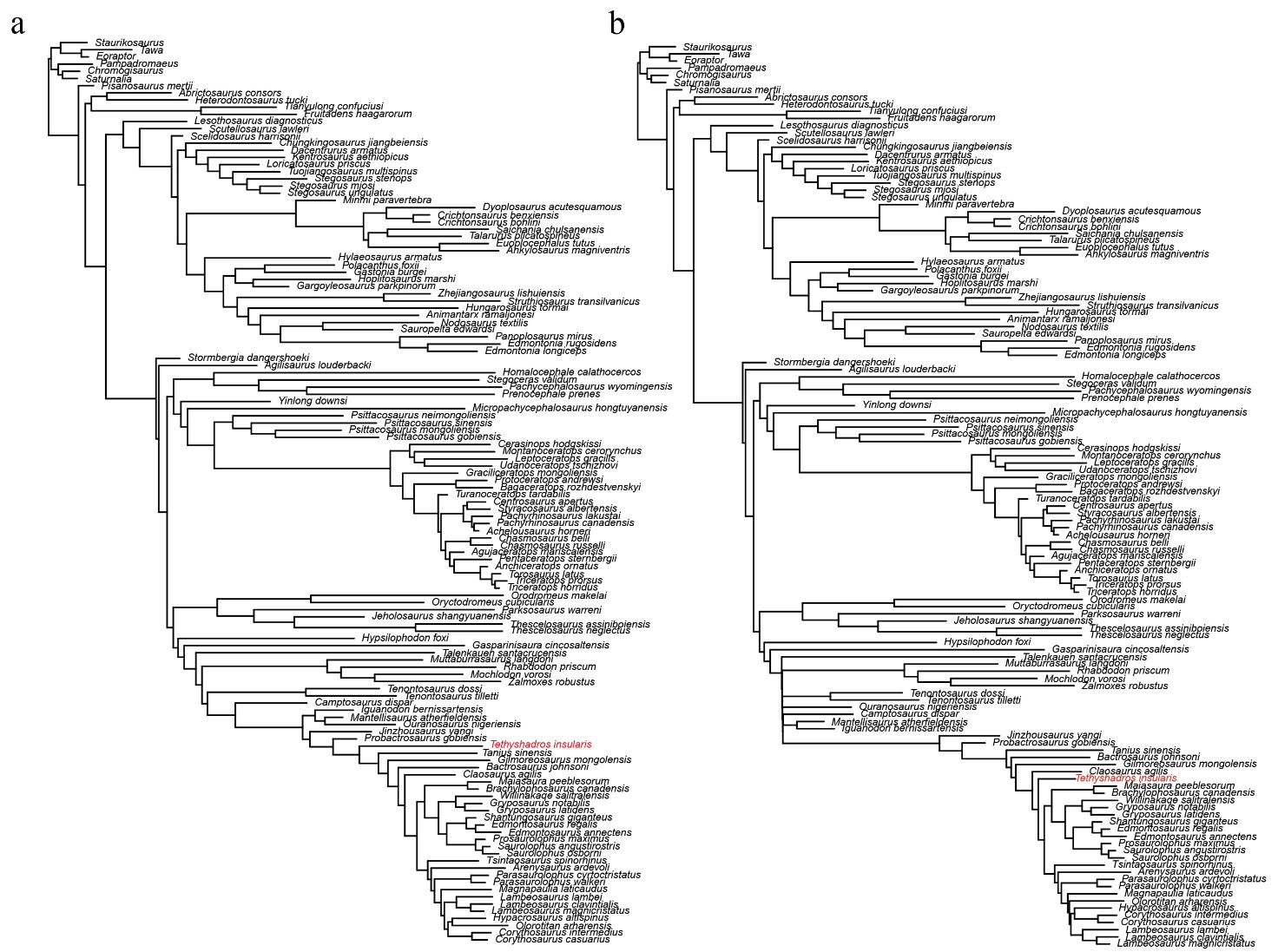
**Figure S26.** **Extended consensus tree of Ornithischia based on the one used by Benson et al. 2018 (a) and updated according to our recovered topology for Hadrosauriformes (b)**


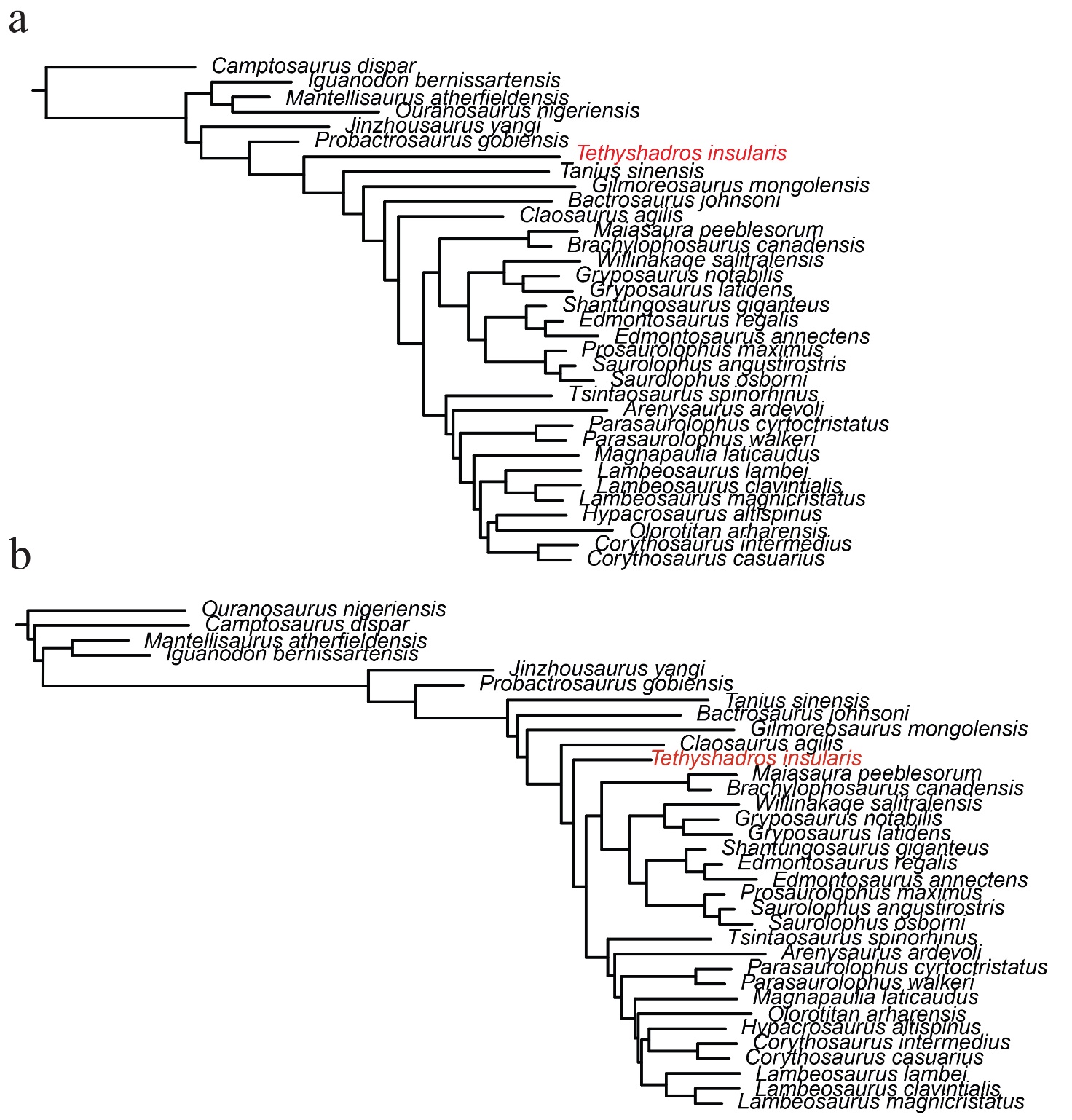
**Figure S27.** **Details of the consensus tree of Hadrosauriformes based on the one used by Benson et al. 2018 (a) and updated according to our maximum parsimony analysis (b).**


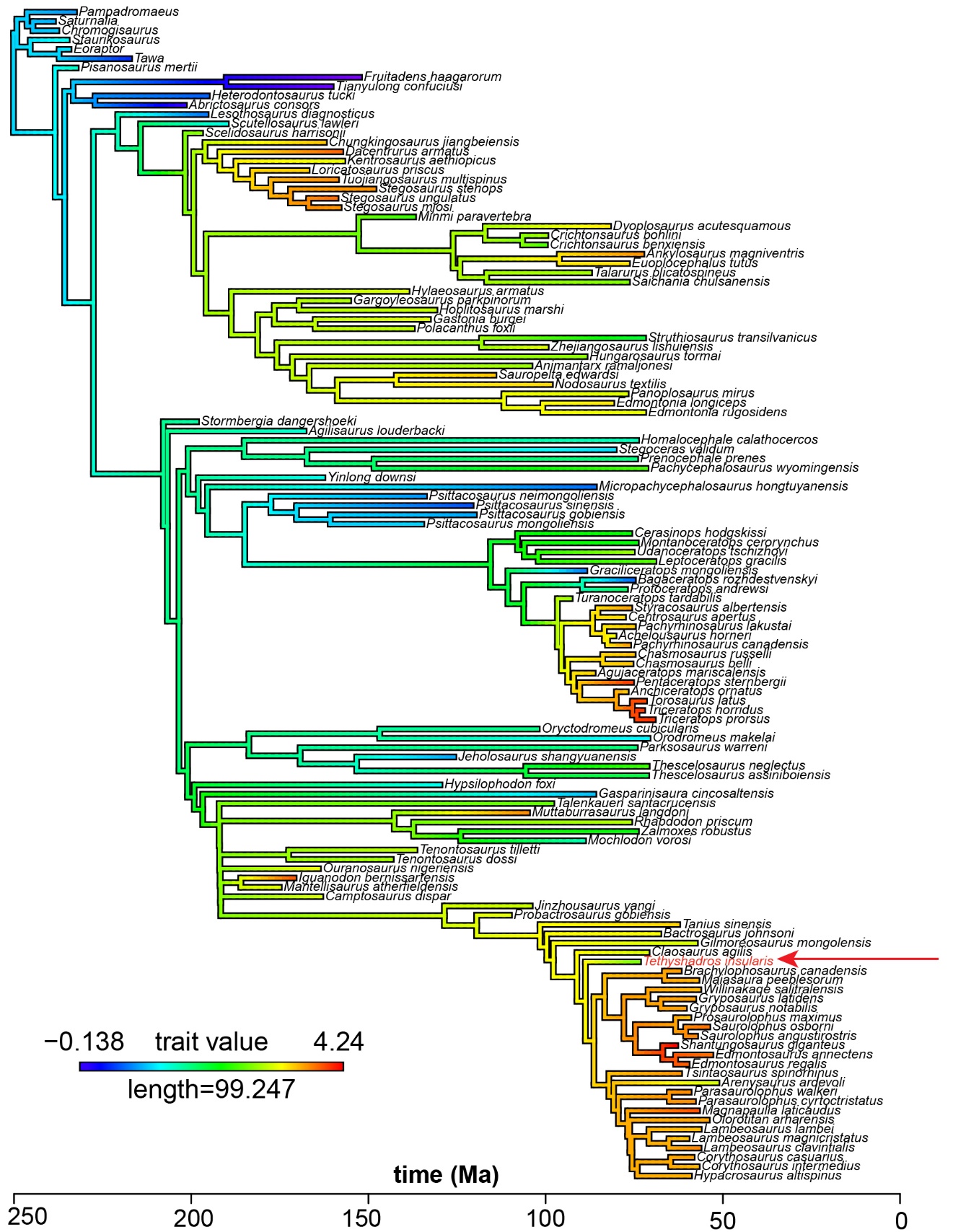


**Figure S28.** **Body mass ancestral state reconstruction for Ornithischia used in our OU-evolutionary modelling.**


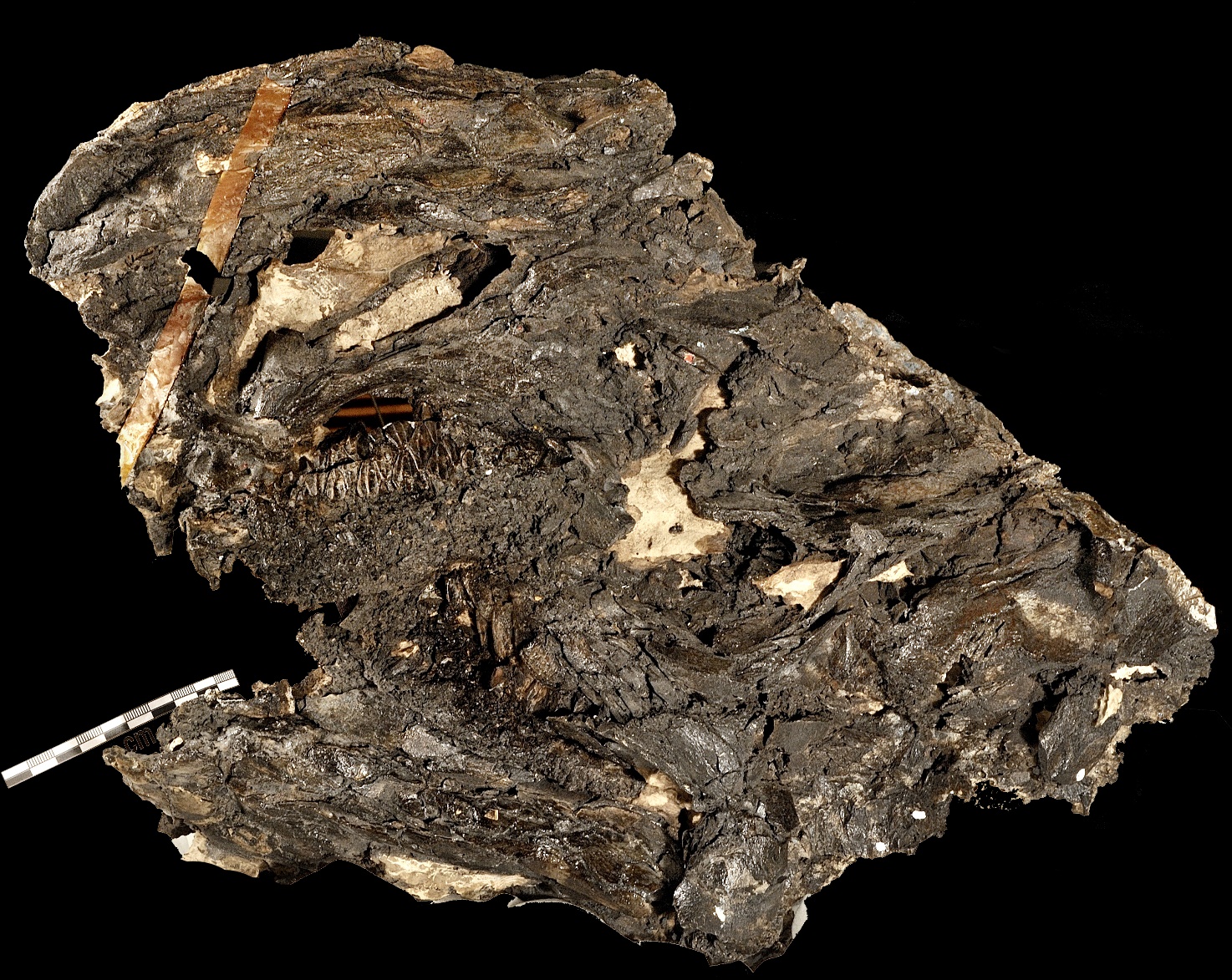
**Figure S29.** **Additional skull of *Tethysahdros insularis* (SC 57026)*.***


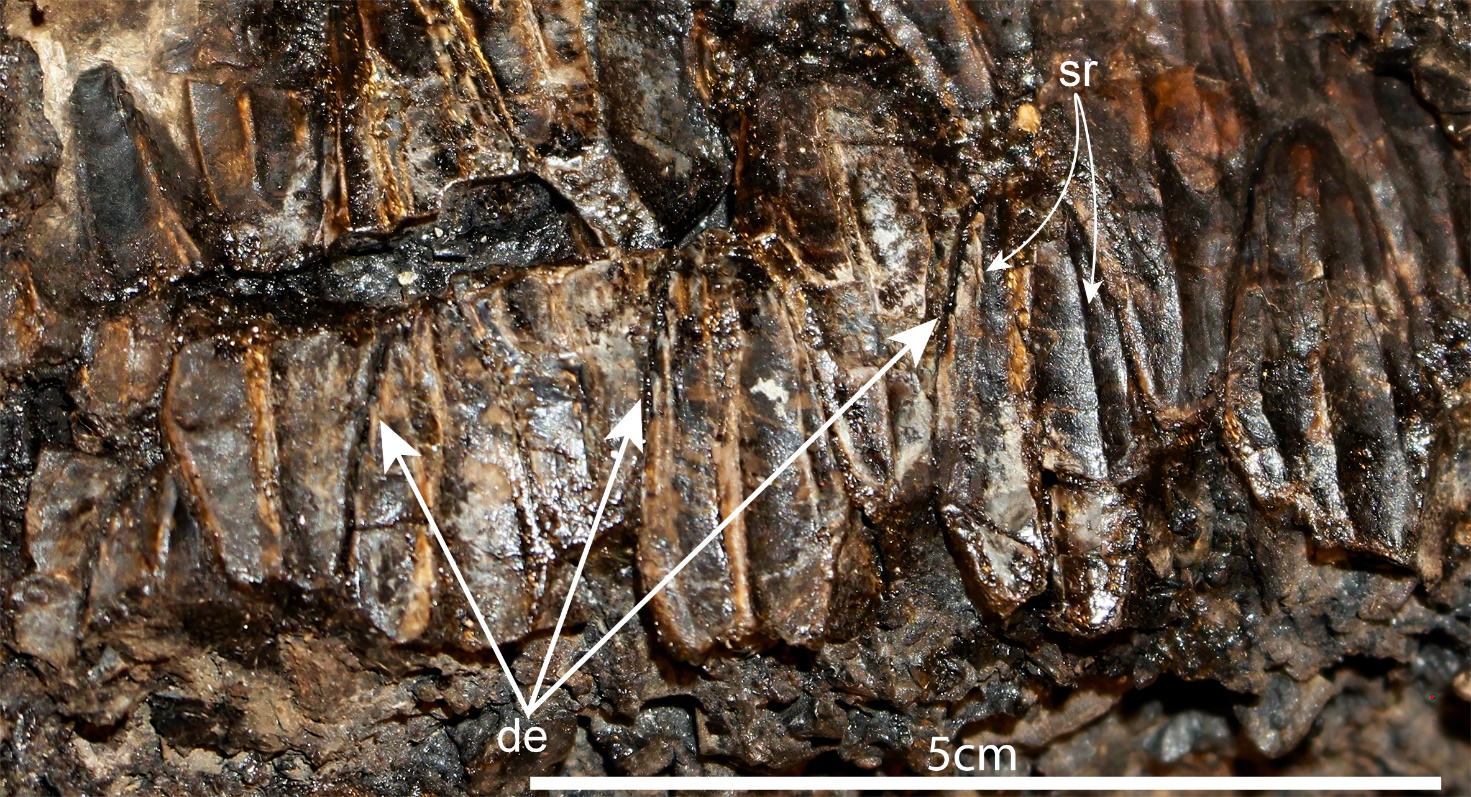
**Figure S30.** **Details of the dentary teeth in SC 57026. Abbreviations: de, marginal denticles; sr, secondary ridge.**


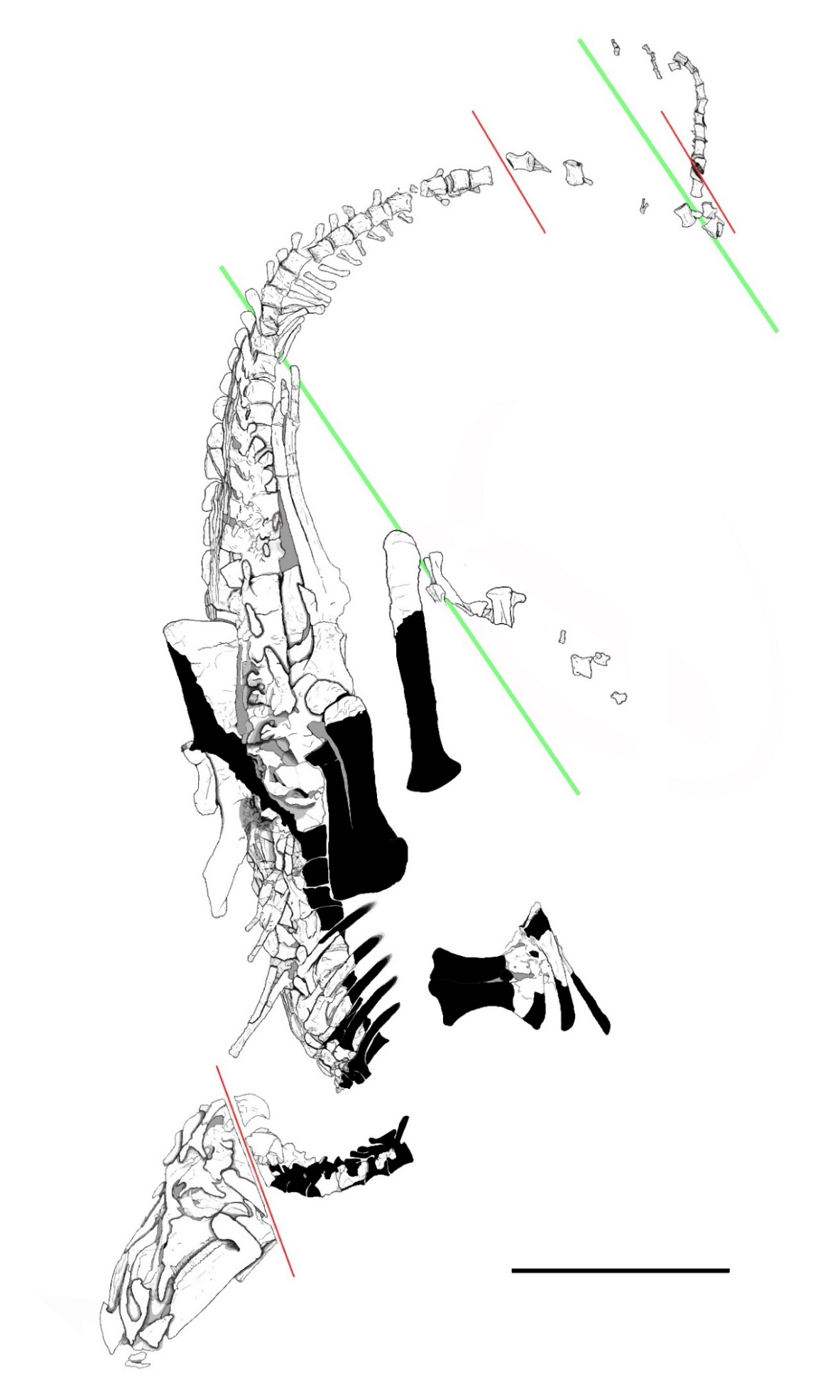


**Figure S31.** **Complete interpretative drawing of SC 57247 skeleton as used in Fig. 2c. Scale bar = 50 cm. Red lines represent artificial cuts made during the extraction while the green lines are indicative of folding planes.**

**Data S1. Anteroposterior length of caudal centra in *Tethyshadros insularis***

**Data S2. Chronostratigraphic ranges of Hadrosauriformes taxa used to calibrate strict consensus tree reported here.**

**Data S3. Nexus file for phylogenetic analysis including the old scoring of *Tethyshadros* as incorporated in the original matrix by Takasaki et al. 2020, the individual-based scoring of SC 57021 and SC 57247 and the comprehensive scoring including ontogenetically-relevant character for this taxon.**

**Data S4. Primary photographic material for *Tethyshadros* osteohistology using petrographic microscopy available on FigShare under CC BY 4.0 licence (DOI: 10.6084/m9.figshare.16918672).**

**References**

1. Vlahović, I., Tišljar, J., Velić, I. & Matičec, D. Evolution of the Adriatic Carbonate Platform: Palaeogeography, main events and depositional dynamics. *Palaeogeography, Palaeoclimatology, Palaeoecology* **220**, 333–360 (2005).

2. Zarcone, G. *et al.* A possible bridge between Adria and Africa: New palaeobiogeographic and stratigraphic constraints on the Mesozoic palaeogeography of the Central Mediterranean area. *Earth-Science Reviews* **103**, 154–162 (2010).

3. Dercourt, J., Gaetani, M., Vrielynck, B., & Commission de la carte géologique du monde. Atlas peri-tethys palaeogeographical maps. (2000).

4. Picotti, V. *et al.* Change from rimmed to ramp platform forced by regional and global events in the Cretaceous of the Friuli-Adriatic Platform (Southern Alps, Italy). *Cretaceous Research* **104**, 104177 (2019).

5. Jurkovšek, B. *et al.* Geology of the Classical Karst Region (SW Slovenia–NE Italy). *Journal of Maps* **12**, 352–362 (2016).

6. Chiocchini, M., Pampaloni, M.L., Pichezzi, R.M. Microfacies and microfossils of the Mesozoic carbonate successions of Latium and Abruzzi (Central Italy). 269 (2012).

7. Frijia, G., Parente, M., Di Lucia, M. & Mutti, M. Carbon and strontium isotope stratigraphy of the Upper Cretaceous (Cenomanian-Campanian) shallow-water carbonates of southern Italy: Chronostratigraphic calibration of larger foraminifera biostratigraphy. *Cretaceous Research* **53**, 110–139 (2015).

8. Steuber, T., Korbar, T., Jelaska, V. & Gušić, I. Strontium-isotope stratigraphy of Upper Cretaceous platform carbonates of the island of Brač (Adriatic Sea, Croatia): implications for global correlation of platform evolution and biostratigraphy. *Cretaceous Research* **26**, 741–756 (2005).

9. Cestari, R. & Sartorio, D. *Rudists and facies of the Periadriatic Domain, PERSONAL COPY*. (1995).

10. Velić, I. Stratigraphy and Palaeobiogeography of Mesozoic Benthic Foraminifera of the Karst Dinarides (SE Europe) - PART 1. *Geologia Croatica* **60**, 1–60 (2007).

11. Otoničar, B. Upper Cretaceous to Paleogene Forbulge Unconformity Associated With Foreland Basin Evolution (Kras, Matarsko Podolje and Istria; SW Slovenia and NW Croatia). *AC* **36**, (2007).

12. Sandro Venturini. Episodi continentali e dulcicoli ed eventi biostratigrafici nella sezione Campaniano-Maastrichtiana di Cotici (M.te San Michele, Gorizia). *Natura Nascosta n.36* (2008).

13. Serra-Kiel, J. *et al.* Larger foraminiferal biostratigraphy of the Tethyan Paleocene and Eocene. *Bulletin de la Société Géologique de France* **169**, 281–299 (1998).

14. Pignatti, J. & Papazzoni, C. A. Oppelzones and their heritage in current larger foraminiferal biostratigraphy. *Lethaia* **50**, 369–380 (2017).

15. Caus, E., Bernaus, J., Boix, C., Calonge, A. & Perez, R. *Upper Cretaceous Shallow Benthic Biozones (KSBZ): a Preliminary Report*. (2003).

16. Caus, E., Parente, M. & Hottinger, L. A biozonation (KSBZ) based on shallow benthic, mainly larger foraminifera from the Upper Cretaceous of the Pyrenees. *Forams 2010, Universität Bonn, abstract book* 70–71 (2010).

17. Caus, E. *et al.* *Biostratigraphy of Late Cretaceous carbonate platforms based on larger foraminifera: the Late Cretaceous shallow benthic zones (LKSBZ)*. (2014).

18. Fleury, J.-J. Rhapydioninidés du Campanien-Maastrichtien en région méditerranéenne : Les genres Murciella, Sigalveolina n. gen. et Cyclopseudedomia. *Carnets de géologie (Notebooks on geology)* **18**, 233–280 (2018).

19. Chiocchini, M., Farinacci, A., Mancinelli, A., Molinari, V. & Potetti, M. Biostratigrafia a foraminiferi, dasicladali e calpionelle delle successioni carbonatiche mesozoiche dell’Appennino Centrale (Italia). (1994).

20. Fleury, J.-J. Cuvillierinella salentina (Foraminifera, Rhapydioninidae) and its kinship in the Western Mediterranean area during the Campanian–Maastrichtian. *Revue de Micropaléontologie* **59**, 200–224 (2016).

21. Fleury, J.-J. & Özkan, R. Metacuvillierinella sireli n. sp., a Campanian Rhapydioninidae (Foraminifera), from southeast Turkey. New considerations on the endoskeleton and particularities of the family, with a specialized lexicon. *Carnets de géologie (Notebooks on geology)* **2020**, 165–212 (2020).

22. Schlüter, M., Steuber, T. & Parente, M. Chronostratigraphy of Campanian–Maastrichtian platform carbonates and rudist associations of Salento (Apulia, Italy). *Cretaceous Research* **29**, 100–114 (2008).

23. Consorti, L., Frijia, G. & Caus, E. Rotaloidean foraminifera from the Upper Cretaceous carbonates of Central and Southern Italy and their chronostratigraphic age. *Cretaceous Research* **70**, 226–243 (2017).

24. Vicedo, V., Frijia, G., Parente, M. & Caus, E. The Late Cretaceous Genera Cuvillierinella, Cyclopseudedomia, and Rhapydionina (Rhapydioninidae, Foraminiferida) in Shallow-Water Carbonates of Pylos (Peloponnese, Greece). *Journal of Foraminiferal Research* **41**, 167–181 (2011).

25. Alessandro Palci. Ricostruzione Paleoambientale del Sito Fossilifero Senoniano del Villaggio del Pescatore (Trieste). (Università degli Studi di Trieste, 2003).

26. Tarlao, A., Tentor, M., Tunis, G., Venturini, S. Evidence of a tectonic phase in the Lower Senonian of the Villaggio del Pescatore area. *Gortania Atti Museo Friulano Storia Naturale* 135–142 (1993).

27. Schlagintweit, F. Agglutinated conical foraminifera (Orbitolinidae, Coskinolinidae) from the Upper Cretaceous (Campanian) of Greece, with description of Paracoskinolina klokovaensis n. sp. *Acta Palaeontologica Romaniae* **17**, 83–94 (2021).

28. Moro, A., Velić, I., Mikuž, V. & Horvat, A. Microfacies characteristics of carbonate cobble from Campanian of Slovenj Grandec(Slovenia): Implications for determining the Fleyrana Adriatica de Castro, Drobne and Gusić paleoniche and extending the biostratigraphic range in the Tethyan realm. *Rudarsko-geološko-naftni zbornik* **33**, 1–12 (2018).

29. Dalla Vecchia, F. M. *Tethyshadros insularis* , a new hadrosauroid dinosaur (Ornithischia) from the Upper Cretaceous of Italy. *Journal of Vertebrate Paleontology* **29**, 1100–1116 (2009).

30. Dalla Vecchia, F. Telmatosaurus and the other hadrosaurids of the Cretaceous European Archipelago. An update. *Natura Nascosta* **39**, 1–18 (2009).

31. Dalla Vecchia, F. Cretaceous dinosaurs in the Adriatic-Dinaric Carbonate Platform (Italy and Croatia): paleoenvironmental implications and paleogeographical hypotheses. *Memorie della Società Geologica Italiana* **57**, 89–100 (2002).

32. Fleury, J.-J. Le genre Murciella (Foraminifère, Rhapydionininae), dans le Crétacé supérieur de Grèce (zone de Gavrovo-Tripolitza). *Geobios* **12**, 149–185 (1979).

33. Bignot, G. *Recherches stratigraphiques sur les calcaires du crétacé supérieur et de l’éocène d’Istrie et des régions voisines: essai de révision du liburnien*. (Université de Paris VI, U.E.R. 63, Sciences de la terre, 1972).

34. Fourcade, E. Murciella cuvillieri n. gen. n. sp. nouveau foraminifère du Sénonien supérieur du sud-est de l’Espagne. *Revue de Micropaléontologie* 147–155 (1966).

35. Vicedo, V. Morfoestructura de los Géneros Cretácicos de los Rapydioninidae (Foraminifera). (Universitat Autónoma de Barcelona, 2008).

36. Parente, M. A revised stratigraphy of the Upper Cretaceous to Oligocene units from southeastern Salento (Apulia, southern Italy). (1994).

37. Fabio M Dalla Vecchia. I dinosauri del Villaggio del Pescatore (Trieste): qualche aggiornamento. 21 (2008).

38. Dalla Vecchia, F. A wing metacarpal from Italy and its implications for latest Cretaceous pterosaur diversity. *Geological Society, London, Special Publications* **455**, SP455.1 (2017).

39. Tentor, M. & Venturini, S. Schema stratigrafico e tettonico del Carso isontino.

40. Venturini, S. & Tentor, M. EPISO DI CONTINENTALI E DULCICO LI ED EVENTI BIOSTRATIGRAFICI NELLA SEZIO NE CAMPANIANO - MAASTRICHTIANA DI CO TICI (M.TE SAN MICHELE, GO RIZIA). 18 (2008).

41. Arbulla D., Cotza F., Cucchi F., Dalla Vecchia F.M., De Giusto A., Flora O., Masetti D., Palci A., Pittau P., Pugliese N., Stenni B., Tarlao A., Tunis G. & Zini L. La successione Santoniano–Campaniana del Villaggio del Pescatore (Carso Triestino) nel quale sono stati rinvenuti i resti di dinosauro. in *Guida alle escursioni/excursions guide, Società Paleontologica Italiana* 20–27 (EUT Edizioni Università di Trieste, 2006).

42. Brazzati and Calligaris. Studio preliminare reperti ossei Villaggio del Pescatore. *Atti Museo di Scienze Naturali di Trieste* (1995).

43. Tarlao, A., Tentor, M., Tunis, G., Venturini, S.,. Stop 4: Villaggio del Pescatore. *Atti Museo Geologico Paleontologico Monfalcone* 135–142 (1995).

44. Attura, M. Aspetti paleontologici e geochimico-isotopici di una successione stratigrafica Santoniana-Camapaniana del Villaggio del Pescatore (Ts). (Università degli Studi di Trieste, 1999).

45. Delfino, M., Martin, J. E. & Buffetaut, E. A new species of Acynodon (Crocodylia) from the upper cretaceous (Santonian–Campanian) of Villaggio del Pescatore, Italy. *Palaeontology* **51**, 1091–1106 (2008).

46. Fabio Marco Dalla Vecchia. The unusual tail of Tethyshadros insularis (Dinosauria, Hadrosauroidea) from the Adriatic Island of the European Archipelago. 46 (2020).

47. Dalla Vecchia, F.M. Relazione scientifica finale, scavo paleontologico del Villaggio del Pescatore 1998-1999 & elencazione, identificazione e determinazione preliminare dei reperti. (1999).

48. Muscioni, M.,. Morfometria e variabilità nella coda di Tethyshadros insularis. Undergraduate Thesis. (Alma Mater Studiorum – Università di Bologna, 2021).

49. Xing, H., Mallon, J. C. & Currie, M. L. Supplementary cranial description of the types of Edmontosaurus regalis (Ornithischia: Hadrosauridae), with comments on the phylogenetics and biogeography of Hadrosaurinae. *PLoS ONE* **12**, e0175253 (2017).

50. Sues, H.-D. & Averianov, A. A new basal hadrosauroid dinosaur from the Late Cretaceous of Uzbekistan and the early radiation of duck-billed dinosaurs. *Proc. R. Soc. B* **276**, 2549–2555 (2009).

51. Shibata, M., Jintasakul, P., Azuma, Y. & You, H.-L. A New Basal Hadrosauroid Dinosaur from the Lower Cretaceous Khok Kruat Formation in Nakhon Ratchasima Province, Northeastern Thailand. *PLOS ONE* **10**, e0145904 (2015).

52. McDonald, A. T., Bird, J., Kirkland, J. I. & Dodson, P. Osteology of the Basal Hadrosauroid Eolambia caroljonesa (Dinosauria: Ornithopoda) from the Cedar Mountain Formation of Utah. *PLoS ONE* **7**, e45712 (2012).

53. Gates, T., Horner, J., Hanna, R. & Nelson, R. New Unadorned Hadrosaurine Hadrosaurid (Dinosauria, Ornithopoda) from the Campanian of North America. *Journal of Vertebrate Paleontology* **31**, 798–811 (2011).

54. Prieto-Marquez, A. New information on the cranium of Brachylophosaurus canadensis (Dinosauria, Hadrosauridae), with a revision of its phylogenetic position. *Journal of Vertebrate Paleontology* **25**, 144–156 (2005).

55. Gates, T. A. & Lamb, J. Redescription of Lophorhothon atopus (Ornithopoda: Dinosauria) from the Late Cretaceous of Alabama based on new material. *Can. J. Earth Sci.* (2021) doi:10.1139/cjes-2020-0173.

56. Prieto-Márquez, A., Erickson, G. M. & Ebersole, J. A. Anatomy and osteohistology of the basal hadrosaurid dinosaur *Eotrachodon* from the uppermost Santonian (Cretaceous) of southern Appalachia. *PeerJ* **4**, e1872 (2016).

57. You, H.-L. & Li, D.-Q. A new basal hadrosauriform dinosaur (Ornithischia: Iguanodontia) from the Early Cretaceous of northwestern China. *Can. J. Earth Sci.* **46**, 949–957 (2009).

58. Fowler, E. A. F. & Horner, J. R. A New Brachylophosaurin Hadrosaur (Dinosauria: Ornithischia) with an Intermediate Nasal Crest from the Campanian Judith River Formation of Northcentral Montana. *PLOS ONE* **10**, e0141304 (2015).

59. Wang, X. & Xu, X. A new iguanodontid (Jinzhousaurus yangi gen. et sp. nov.) from the Yixian Formation of western Liaoning, China. *Chin.Sci.Bull.* **46**, 1669–1672 (2001).

60. Forster. The postcranial skeleton of the ornithopod dinosaur Tenontosaurus tilletti: Journal of Vertebrate Paleontology. https://www.tandfonline.com/doi/abs/10.1080/02724634.1990.10011815 (1990).

61. Philipe Taquet. Geologie et paleontologie du gisement de Gadoufaoua (Aptien du Niger). *Cahier Paleontologie* 1–191 (1976).

62. Weishampel, D., Norman, D. & Grigorescu, D. Telmatosaurus transsylvanicus from the Late Cretaceous of Romania: the most basal hadrosaurid dinosaur. *Palaeontology* **36**, 361–385 (1993).

63. Nopsca F. Sexual differences in ornithopodous dinosaurs. 187–200 (1929).

64. Verdú, F. J., Royo-Torres, R., Cobos, A. & Alcalá, L. New systematic and phylogenetic data about the early Barremian Iguanodon galvensis (Ornithopoda: Iguanodontoidea) from Spain. *Historical Biology* **30**, 437–474 (2018).

65. Worthington R., D., Wake, D., B. Patterns of regional variation in the vertebral column of terrestrial salamanders. *ournal of Morphology* (1972).

66. Vaglia, J. L., Babcock, S. K. & Harris, R. N. Tail development and regeneration throughout the life cycle of the four-toed salamander Hemidactylium scutatum. *Journal of Morphology* **233**, 15–29 (1997).

67. Buchholtz, E. A. Vertebral osteology and swimming style in living and fossil whales (Order: Cetacea). *Journal of Zoology* **253**, 175–190 (2001).

68. Buchholtz, E. A. & Schur, S. A. Vertebral osteology in Delphinidae (Cetacea). *Zoological Journal of the Linnean Society* **140**, 383–401 (2004).

69. Felice, R. N. & Angielczyk, K. D. Was Ophiacodon (Synapsida, Eupelycosauria) a Swimmer? A Test Using Vertebral Dimensions. in *Early Evolutionary History of the Synapsida* (eds. Kammerer, C. F., Angielczyk, K. D. & Fröbisch, J.) 25–51 (Springer Netherlands, 2014). doi:10.1007/978-94-007-6841-3_3.

70. David W.E. Hone, W. Scott Persons, Steven C. Le Comber. New data on tail lengths and variation along the caudal series in the non-avialan dinosaurs [PeerJ].

71. Pittman, M., Gatesy, S. M., Upchurch, P., Goswami, A. & Hutchinson, J. R. Shake a Tail Feather: The Evolution of the Theropod Tail into a Stiff Aerodynamic Surface. *PLOS ONE* **8**, e63115 (2013).

72. Norman, D. On the history, osteology, and systematic position of the Wealden (Hastings Group) dinosaur Hypselospinus fittoni (Iguanodontia: Styracosterna). *Zoological Journal of the Linnean Society* **173**, (2014).

73. Horner, J., Weishampel, D. & Forster, C. Hadrosauridae. in *Hadrosauridae* 438–463 (2004). doi:10.1525/california/9780520242098.003.0023.

74. Lull, R. S. & Wright, N. E. Hadrosaurian Dinosaurs of North America. in *Hadrosaurian Dinosaurs of North America* (eds. Lull, R. S. & Wright, N. E.) vol. 40 0 (Geological Society of America, 1942).

75. Benson, R. B. J., Hunt, G., Carrano, M. T. & Campione, N. Cope’s rule and the adaptive landscape of dinosaur body size evolution. *Palaeontology* **61**, 13–48 (2018).

76. Campione, N. E. & Evans, D. C. A universal scaling relationship between body mass and proximal limb bone dimensions in quadrupedal terrestrial tetrapods. *BMC Biology* **10**, 60 (2012).

77. Campione, N. E. & Evans, D. C. The accuracy and precision of body mass estimation in non-avian dinosaurs. *Biological Reviews* **95**, 1759–1797 (2020).

78. Campione, N. E., Evans, D. C., Brown, C. M. & Carrano, M. T. Body mass estimation in non-avian bipeds using a theoretical conversion to quadruped stylopodial proportions. *Methods in Ecology and Evolution* **5**, 913–923 (2014).

79. Takasaki R., et al. Re-examination of the cranial osteology of the Arctic Alaskan hadrosaurine with implications for its taxonomic status. https://journals.plos.org/plosone/article?id=10.1371/journal.pone.0232410 (2020).

80. Xing, H., Prieto-Marquez, A., Wei, G., Ting-Xiang, Y. & Palasiatica, V. Re-evaluation and phylogenetic analysis of Wulgasaurus dongi, a hadrosaurine dinosaur from the Maastrichtian of Northeast China. *Vertebrata Palasiatica* (2012).

81. Prieto-Marquez, A. & Wagner, J. R. Pararhabdodon isonensis and Tsintaosaurus spinorhinus: a new clade of lambeosaurine hadrosaurids from Eurasia. *Cretaceous Research* **30**, 1238–1246 (2009).
